# Modulating Protein-Protein Interactions of the Mitotic Polo-like Kinases to Target Mutant KRAS

**DOI:** 10.1016/j.chembiol.2017.07.009

**Published:** 2017-08-17

**Authors:** Ana J. Narvaez, Suzan Ber, Alex Crooks, Amy Emery, Bryn Hardwick, Estrella Guarino Almeida, David J. Huggins, David Perera, Meredith Roberts-Thomson, Roberta Azzarelli, Fiona E. Hood, Ian A. Prior, David W. Walker, Richard Boyce, Robert G. Boyle, Samuel P. Barker, Christopher J. Torrance, Grahame J. McKenzie, Ashok R. Venkitaraman

**Affiliations:** 1 Medical Research Council Cancer Unit, University of Cambridge, Hutchison/MRC Research Centre, Hills Road, Cambridge CB2 0XZ, UK; 2 Department of Chemistry, University of Cambridge, Lensfield Road, Cambridge CB2 1EW, UK; 3 University of Cambridge, Theory of Condensed Matter Group, Cavendish Laboratory, 19 J J Thomson Avenue, Cambridge CB3 0HE, UK; 4 Department of Oncology, University of Cambridge, Hutchison/MRC Research Centre, Hills Road, Cambridge CB2 0XZ, UK; 5 Wellcome Trust-Medical Research Council Cambridge Stem Cell Institute, University of Cambridge, Tennis Court Road, Cambridge CB2 1QR, UK; 6 Division of Cellular and Molecular Physiology, Crown Street, University of Liverpool, Liverpool L69 3BX, UK; 7 Sentinel Oncology Ltd., Cambridge Science Park, Milton Road, Cambridge CB4 0EY, UK; 8 PhoreMost Ltd., Babraham Research Campus, Cambridge CB22 3AT, UK

**Keywords:** protein-protein interactions, PPI inhibitor, Polo-like kinase, Polo-box domain, PLK1, PLK4, mutant KRAS, c-MET, cancer therapy

## Abstract

Mutations activating KRAS underlie many forms of cancer, but are refractory to therapeutic targeting. Here, we develop Poloppin, an inhibitor of protein-protein interactions via the Polo-box domain (PBD) of the mitotic Polo-like kinases (PLKs), in monotherapeutic and combination strategies to target mutant KRAS. Poloppin engages its targets in biochemical and cellular assays, triggering mitotic arrest with defective chromosome congression. Poloppin kills cells expressing mutant KRAS, selectively enhancing death in mitosis. PLK1 or PLK4 depletion recapitulates these cellular effects, as does PBD overexpression, corroborating Poloppin's mechanism of action. An optimized analog with favorable pharmacokinetics, Poloppin-II, is effective against KRAS-expressing cancer xenografts. Poloppin resistance develops less readily than to an ATP-competitive PLK1 inhibitor; moreover, cross-sensitivity persists. Poloppin sensitizes mutant KRAS-expressing cells to clinical inhibitors of c-MET, opening opportunities for combination therapy. Our findings exemplify the utility of small molecules modulating the protein-protein interactions of PLKs to therapeutically target mutant KRAS-expressing cancers.

## Highlights

• Poloppins target the PBD of human PLKs causing mitotic death in KRAS-mutant cells
• Depletion of PLK1 or PLK4 recapitulates the cellular effects of Poloppins
• Poloppins reduce drug resistance *in vitro*, and act against KRAS-mutant xenografts
• Poloppins sensitize KRAS-mutant cells to clinically used c-MET inhibitors

## Graphical Abstract

**Figure:**
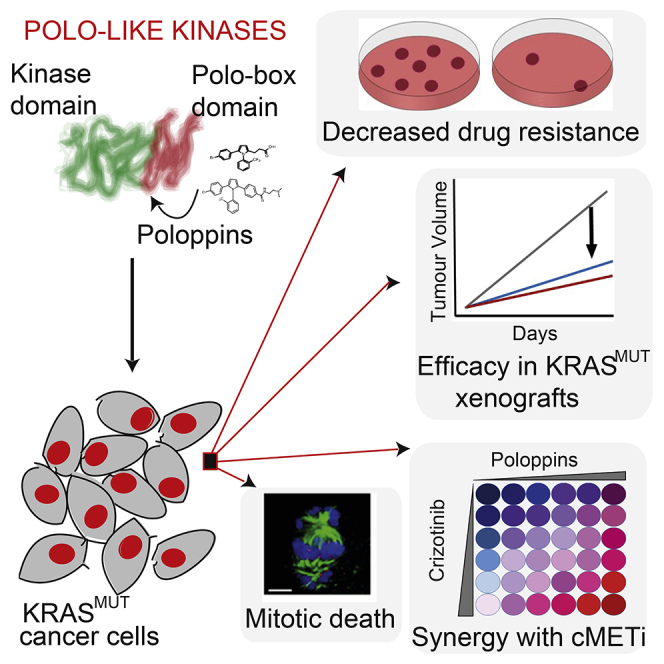

## Teaser

Narvaez et al. report the development of Poloppins, compounds that target the protein-protein interactions of human Polo-like kinases via their Polo-box domain, in monotherapeutic or combination strategies to treat KRAS-mutant cancers.

## Introduction

Human Polo-like kinase 1 (PLK1) regulates multiple events that lead to accurate chromosome segregation during cell division (reviewed in Bruinsma et al., 2012). It comprises an N-terminal kinase catalytic domain, and a C-terminal segment with tandem motifs that form the Polo-box domain (PBD). The PBD engages pSer/pThr phosphopeptide substrates to recruit PLK1 to mitotic structures (Elia et al., 2003a), direct PLK1 kinase activity (Kang et al., 2006), and mediate reaction-diffusion mechanisms for centrosome assembly (Mahen et al., 2011). Homologous PBDs, in which key residues implicated in phosphopeptide substrate recognition are conserved, also occur in the related human Polo-like kinases PLK2, PLK3, and PLK4 (reviewed in Archambault and Glover, 2009).

PLK1's essential role in mitosis, and its dysregulation in several different forms of human cancer, has prompted efforts to create small-molecule inhibitors (reviewed in Liu, 2015). Potent ATP-competitive small-molecule inhibitors of PLK1 kinase activity are in clinical development (reviewed in Gjertsen and Schoffski, 2015). Moreover, compounds that inhibit PBD binding to phosphopeptide substrates are reported to induce mitotic arrest and apoptosis in cancer cell lines at micromolar concentrations (Reindl et al., 2008, Scharow et al., 2015, Watanabe et al., 2009, Yuan et al., 2011); but several have recently been identified as non-specific protein alkylators (Archambault and Normandin, 2017).

Mutations activating the small GTP-binding protein KRAS, which occur frequently in human cancers, have proven largely refractory to therapeutic targeting (reviewed in McCormick, 2015). Inhibitors unique to the G12C KRAS mutant have been described (Ostrem et al., 2013), as have inhibitors of an essential interaction between KRAS and the prenyl-binding protein, PDEδ (Chandra et al., 2012, Zimmermann et al., 2013). An RNAi screen for factors whose depletion inhibits the growth of mutant KRAS G12D-expressing cancer cell lines identified PLK1 and components of the APC/C ubiquitin-conjugating holoenzyme (Luo et al., 2009). Genetic modulation of the pathway linking PLK1 to APC/C activation curtailed the proliferation of mutant KRAS G12D-expressing cancer cells, while ATP-competitive PLK1 inhibitors suppressed their growth as xenografts.

Here, we identify compounds that modulate protein-protein interactions of the structurally related PLK kinases via the PBD domain, and demonstrate their utility in strategies to target mutant KRAS. Poloppin (**Polo p**rotein-**p**rotein interaction **in**hibitor) kills cells expressing mutant KRAS in two-dimensional or organoid cultures. An optimized analog (Poloppin-II) is effective against KRAS-expressing cancer xenografts after systemic oral administration. Notably, Poloppin sensitivity persists in cancer cells resistant to an ATP-competitive PLK1 inhibitor, and Poloppin sensitizes mutant KRAS-expressing cells to clinically used inhibitors of the MET tyrosine kinase, opening opportunities for combination therapy.

## Results

### A Drug-like Inhibitor of Phosphopeptide Binding by PLK1 PBD

**Figure 1:**
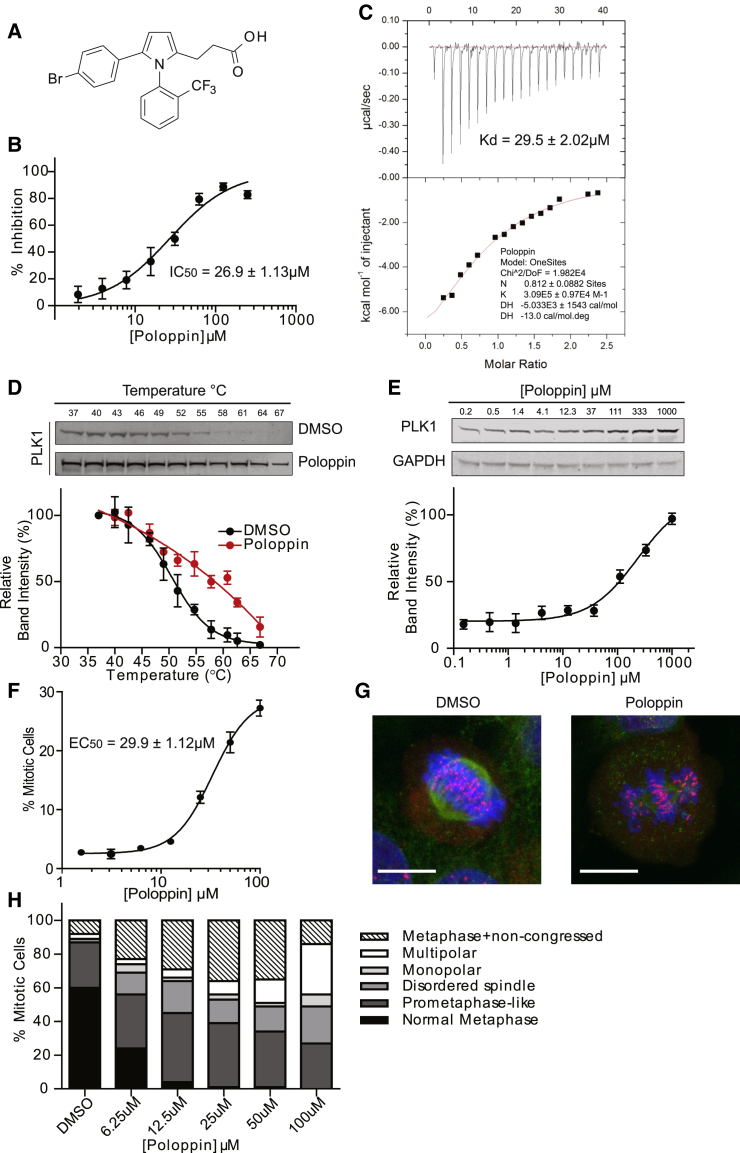
Poloppin Is a Drug-like Inhibitor of Phosphopeptide Binding by PLK1 PBD (A) Structure of Poloppin. (B) Poloppin competitively inhibits the binding of a TAMRA-labeled substrate peptide to the PLK1 PBD in an FP assay. (C) Isothermal titration calorimetry of Poloppin binding to the PBD domain of PLK1. (D) Poloppin stabilizes PLK1 in a cellular thermal shift assay (CeTSA) in HeLa cell lysates. (E) ITDRF_CETSA_ at a constant temperature of 67°C in HeLa lysates treated with Poloppin. (F) Poloppin induces dose-dependent mitotic arrest. (G) Representative images of U2OS cells treated with Poloppin. Green, tubulin; blue, DNA; red, kinetochores. Scale bars, 15 μm. (H) Quantification of Poloppin-induced mitotic phenotypes in U2OS cells. The frequency of observed mitotic phenotypes is displayed. Data represent the mean of three independent experiments ± SEM. See also Figure S1.

We screened a diversity library of 120,000 drug-like molecules (Huggins et al., 2011), using a fluorescence polarization (FP) assay (Figures S1A–S1C), to identify compounds that inhibit the binding of the human PLK1 PBD with a tetramethyl-6-carboxy-rhodamine (TAMRA)-labeled PLK1 PBD consensus binding phosphopeptide. Close analogs of the primary hits were selected by Tanimoto similarity using extended-connectivity fingerprints (Xia et al., 2004), before experimental validation. Poloppin (Figure 1A), a 438 Da compound identified in this way, is lipophilic but soluble at up to 1 mM in 5% DMSO, contains no obvious chemical toxicophores or reactive groups, and therefore was regarded as a suitable starting point for development. Poloppin exhibits a half maximal inhibitory concentration (IC_50_) of 26.9 μM by FP (Figure 1B), while isothermal titration calorimetry reveals that it directly binds to the PLK1 PBD with a K_d_ of 29.5 μM (Figure 1C).

### Poloppin Engages PLK1 to Trigger Mitotic Arrest with Non-congressed Chromosomes

We tested whether Poloppin engages cellular PLK1 using the recently described cellular thermal shift assay (CeTSA). CeTSA detects cellular target engagement via changes in the thermal stability of the drug-bound target, but does not track directly with the potency of compounds in biochemical or phenotypic assays (Jafari et al., 2014, Martinez Molina et al., 2013, Martinez Molina and Nordlund, 2016). PLK1 protein exhibits increased thermal stability following treatment with a fixed dose of 1 mM Poloppin compared with control (Figure 1D). Moreover, exposure of cell lysates at a constant 67°C to increasing doses of Poloppin, using isothermal dose-response fingerprint (ITDRF)-CeTSA, demonstrates that cellular PLK1 is stabilized at >40 μM (Figure 1E). Together, these results provide evidence that Poloppin engages PLK1 in the cellular milieu.

Further evidence comes from the cellular phenotypes induced by Poloppin. Poloppin triggers dose-dependent mitotic arrest in human cell lines (Figure 1F), inducing multiple anomalies in mitosis. At a 12.5 μM concentration, <5% of cells exhibit normal metaphase chromosome alignment, instead arresting with bipolar or disordered spindles and non-congressed chromosomes (Figures 1H and 1G). Poloppin-induced mitotic arrest with non-congressed chromosomes is dissimilar from that induced by ATP-competitive PLK1 inhibitors, which typically suppress bipolar spindle formation (Gilmartin et al., 2009, Hanisch et al., 2006, Lenart et al., 2007).

Collectively, these findings suggest that an early requirement for PLK1 kinase activity during mitotic progression is distinct from the role of substrate engagement by the PBD (Hanisch et al., 2006). Indeed, Poloppin elicits mitotic phenotypes at relatively low concentrations compared with its apparent activity in biochemical assays, consistent with the notion that the compound competitively inhibits PBD binding to functionally relevant substrates present at low cellular abundance, rather than acting as an ATP-competitive catalytic inhibitor.

### Poloppin Kills Cells Expressing Mutant KRAS in Two-Dimensional and Organoid Cultures

**Figure 2:**
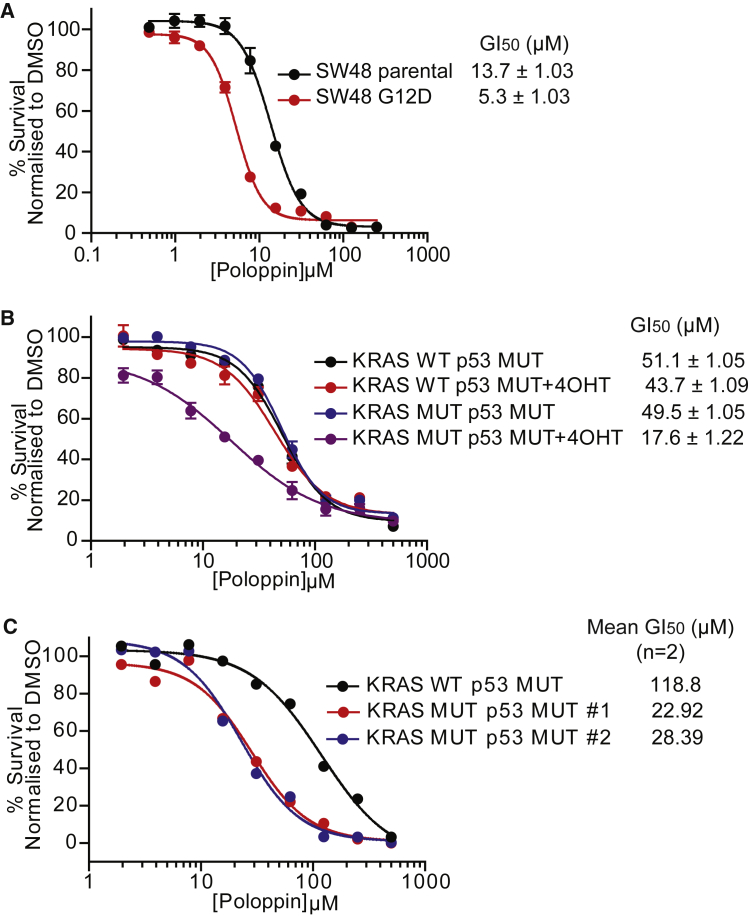
Poloppin Preferentially Kills Cells Expressing Mutant KRAS in Two-Dimensional and Organoid Cultures (A) Cell viability in SW48 isogenic parental or KRAS^G12D^ cell lines. (B) Cell viability in MEFs from either a KRAS wild-type, p53^R172H^ (KRAS WT p53 MUT), or a mutant KRAS^G12D^, p53^R172H^ (KRAS MUT p53 MUT) strain. (C) Viability in murine pancreatic organoids from either a KRAS wild-type, p53^R172H^ (KRAS WT p53 MUT) or mutant KRAS^G12D^, p53^R172H^ (KRAS MUT p53 MUT 1 and 2) strain. Data represent the mean of three independent experiments ± SEM unless otherwise stated.

Mutant KRAS expression sensitizes cells to genetic or pharmacologic modulation of PLK1 (Luo et al., 2009) by ∼2-fold over controls. Similarly, SW48 cells expressing KRAS G12D exhibit ∼2-fold greater sensitivity to Poloppin than isogenic controls (Figure 2A), as do primary murine embryonic fibroblasts (MEFs) genetically engineered to acutely express mutant KRAS G12D when treated with 4-hydroxy tamoxifen (Figure 2B). Poloppin has a greater (∼4- to 5-fold; average, 4.7-fold) inhibitory effect on the growth of pancreatic organoids formed by pancreatic epithelial cells derived from mice genetically engineered to express mutant KRAS G12D in the pancreas (KRAS MUT p53 MUT 1 and 2) compared with KRAS wild-type (KRAS WT p53 MUT) controls (Figure 2C).

### Poloppin Exerts its Cellular Effects via the PBD Domains of PLKs

Poloppin does not bind (Figure S1D) to the kinase catalytic domains of either PLK1 or its homologs, PLK2, PLK3, or PLK4, using the DiscoverX KinomeScan assay (Fabian et al., 2005, Karaman et al., 2008). No significant binding was observed to 51 other protein kinases bearing structural or functional similarity to the Polo-like kinases. However, Poloppin inhibits phosphopeptide substrate engagement to the PBD of PLK1 (Figure 1B; IC_50_ = 29 μM), and with 3-fold less activity (Figure S1E; IC_50_ = 88 μM), to the closely related PBD domain of human PLK2 when tested by FP. Since PLK1–4 all harbor homologous PBD domains (van de Weerdt et al., 2008), we tested whether Poloppin might exert its cellular effects via the inhibition of PLK2, PLK3, or PLK4, besides PLK1.

**Figure 3:**
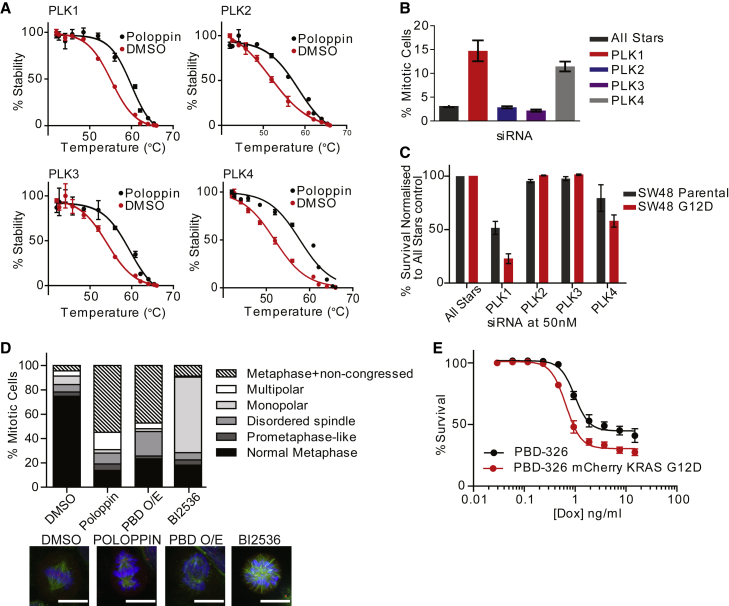
Poloppin Exerts Its Cellular Effects via the PBD Domains of PLKs (A) Thermal stability assay for PLK1–4 using NanoLuc fusion proteins. (B) Mitotic index assay in HeLa Flp-In T-REx cells. (C) Cell viability in the SW48 isogenic parental or KRAS^G12D^ cell lines following depletion of PLKs using indicated siRNAs. (D) Quantitation of mitotic phenotypes. HeLa cells engineered to overexpress PLK1-PBD upon addition of doxycycline were treated with compounds at EC_50_ concentrations or with doxycycline (PBD O/E) for 16 hr. Mitotic phenotypes were quantified by immunofluorescence imaging. Green, tubulin; blue, DNA; red, kinetochores. Scale bars, 15 μm. (E) Cell viability of HeLa cells expressing doxycycline-inducible PLK1-PBD, with or without constitutively overexpressed mCherry-KRAS G12D. Data represent the mean of three independent experiments ± SEM. See also Figure S2.

In cells transiently expressing fusions between PLK1–4 and the reporter Nanoluciferase (NanoLuc) (Hall et al., 2012, Masser et al., 2016), Poloppin at 100 μM increases the thermal stability of all four NanoLuc-PLK fusion proteins (Figure 3A), but not an irrelevant NanoLuc-p38α MAPK fusion protein (Figure S2). These results suggest that Poloppin engages PLK1–4.

We therefore tested whether the selective depletion of PLK1–4 using RNAi could recapitulate Poloppin-induced phenotypes. Depletion of PLK1 or PLK4 but not PLK2 or PLK3 triggers mitotic arrest (Figure 3B). Moreover, PLK1 or PLK4 depletion preferentially decreased the survival of SW48 cells expressing KRAS G12D over that of isogenic control cells (Figure 3C), whereas PLK2 or PLK3 depletion had no such effect. Thus, our findings suggest that PLK1 and PLK4 inhibition underlies the activity of Poloppin in triggering mitotic arrest, and in preferentially killing cancer cells that express mutant oncogenic *KRAS*.

Next, we tested whether inducible overexpression of the PLK1 PBD in cells, which is expected to mimic the effects of Poloppin by competitively inhibiting substrate engagement to the PBD domains, could recapitulate these phenotypic effects. Indeed, cells induced to overexpress the PBD by treatment with doxycycline (Figure 3D) undergo a characteristic metaphase arrest with non-congressed chromosomes, which is typical of the effect induced by Poloppin (Figures 1G and 1H). Inducible PBD expression also preferentially reduces in a dose-dependent manner the viability of cells co-expressing mutant KRAS, over that of the parental cells (Figure 3E), by ∼2-fold, as expected. Thus, collectively, our findings provide genetic evidence to corroborate that the cellular effects of Poloppin are mediated via the PBD domain.

### Poloppin Sensitizes Mutant KRAS-Expressing Cells to Death in Mitosis

**Figure 4:**
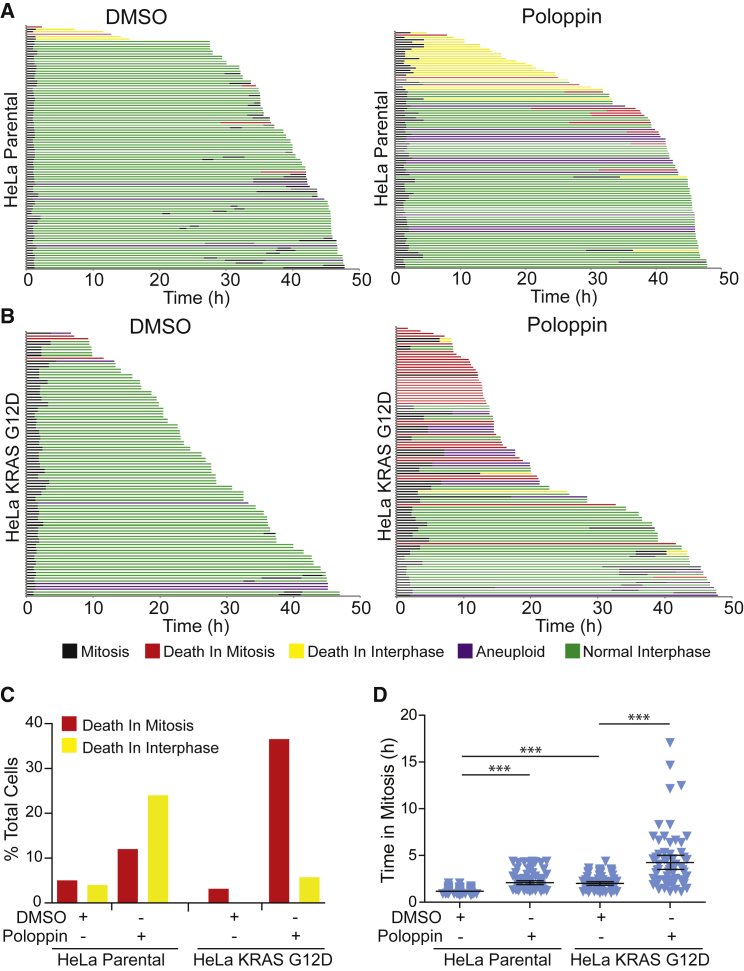
Poloppin Sensitizes Cells Expressing Mutant KRAS to Death in Mitosis (A and B) Live-cell imaging of (A) parental HeLa cells and (B) HeLa cells expressing KRAS^G12D^ treated with DMSO or with Poloppin at their GI_50_ dose. Each horizontal bar represents the fate of a single cell visualized by serial time-lapse imaging. Colors indicate the different cell fates. (C) Quantitation of the percentage and type of cell death. (D) Graph showing time spent in mitosis. ***p < 0.001. Error bars represent the 95% confidence interval from the mean.

Using live-cell imaging to track the fate of single wild-type HeLa cells exposed to Poloppin (Figure 4), we find that Poloppin exposure prolongs mitosis from an average of 60 to 100 min (compare black segments in Figures 4A and 4B; tabulated in Figure 4D), and increases the frequency of aneuploid divisions to 38% (purple segments and yellow segments), compared with 4% after DMSO treatment. Typically, Poloppin-treated wild-type cells exit mitosis to form binucleate or anucleate daughters, which undergo death during the subsequent interphase (yellow segments).

By contrast, Poloppin treatment sensitizes mutant KRAS-expressing HeLa cells to death in mitosis. Induction of a chromosomally integrated, doxycycline-inducible KRAS G12D transgene increases the time taken for mitosis from 100 to 200 min (compare black segments in Figure 4A versus 4B; tabulated in Figure 4D), consistent with the notion that mutant KRAS dysregulates mitotic progression as recently reported (Perera and Venkitaraman, 2016). However, when mutant KRAS-expressing HeLa cells are exposed to Poloppin (Figure 4B), there is also a sharp increase in the frequency of death in mitosis (red segments) when compared with DMSO-exposed controls (Figure 4C, 3% versus 36%). Collectively, these results suggest that Poloppin preferentially sensitizes mutant KRAS-expressing cells, but not controls, to death in mitosis.

### An Optimized Poloppin Analog Is Effective *In Vivo* against Mutant KRAS-Expressing Xenografts

**Figure 5:**
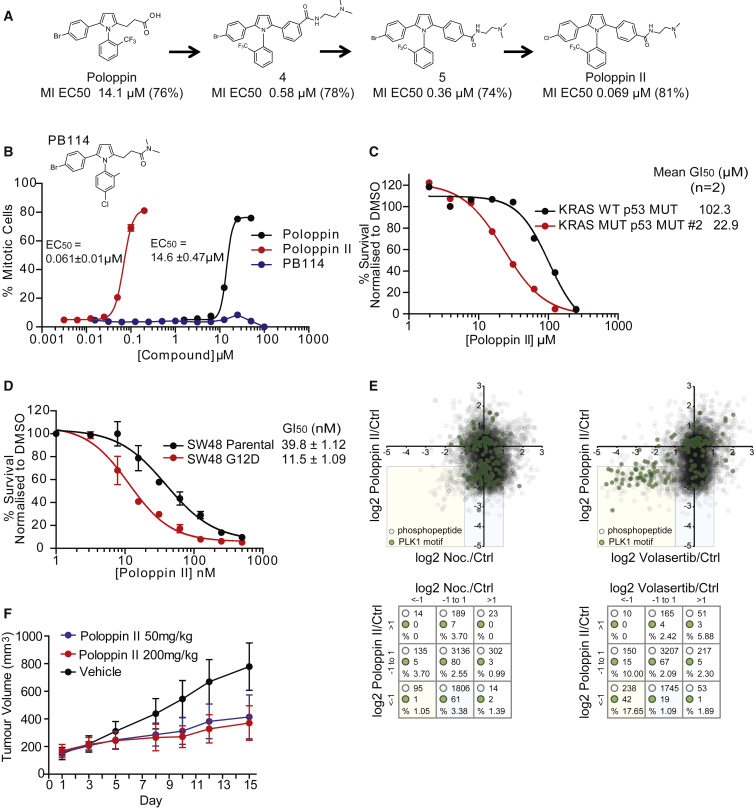
The Optimized Analog Poloppin-II Is Effective by Systemic Oral Administration *In Vivo* Against Mutant KRAS-Expressing Xenografts (A) Synthetic chemistry route from Poloppin to Poloppin-II. The EC_50_ value of each analog in a cellular assay for mitotic arrest is given below its designation, with the maximum percentage of mitotic cells in brackets. (B) Mitotic index assay in HeLa cells treated for 16 hr with Poloppin, Poloppin-II, or the structurally related analog, PB114. (C) Cell viability in KRAS wild-type murine pancreatic organoids (KRAS WT p53 MUT), or organoids expressing KRAS G12D (KRAS MUT p53 MUT). (D) Cell viability in SW48 parental and KRAS G12D isogenic cell lines at 72 hr. Data represent the mean of three independent experiments ± SEM. (E) Mass spectrometric analysis of changes in phosphopeptide abundance induced by Poloppin-II versus Nocodazole or the ATP-competitive PLK1 inhibitor, Volasertib. Pairwise comparisons of the relative abundance of phosphopeptides detected in this analysis are plotted logarithmically to the base 2 (top panels). Green dots indicate phosphopeptides that contain the PLK1 phosphorylation consensus motifs. The boxed, yellow-shaded area in the bottom left-hand quadrant marks phosphopeptides that exhibit a ≥2-fold reduction in abundance in both conditions. The tables below each dot plot show the total number of phosphopeptides, the number of PLK1 motif-containing phosphopeptides, and the percentage of PLK1 motif-containing phosphopeptides in nine different bins defined by (log2) abundance values. (F) Tumor progression in a xenograft model of HCT116 cells expressing KRAS^G13D^ after systemic treatment via oral administration with Poloppin-II. Error bars indicate mean ± SD. See also Figure S3.

An optimized synthetic analog, Poloppin-II (Figure 5A), is soluble at up to ∼100 μM in 5% DMSO, and exhibits no binding at 5 μM to the kinase catalytic domains of PLK1–4, or to 51 other related kinases using the DiscoverX KinomeScreen assay (Figure S3A). It induces mitotic arrest with non-congressed chromosomes similar to that induced by Poloppin (Figure 5B). Poloppin-II exhibits a half maximal effective concentration of 61 nM in a cellular assay for mitotic arrest compared with 14.6 μM for Poloppin, whereas a structurally related analog of Poloppin-II (PB114) is inactive (Figure 5B). Poloppin-II engages PLK1 and PLK4, as detected using NanoLuc fusion proteins, whereas PB114 is less active (Figure S3C). Poloppin-II sensitizes cells expressing mutant KRAS in two-dimensional or organoid cultures by approximately 5-fold (Figures 5C and 5D).

Despite its potency in cellular assays, Poloppin-II competitively inhibits substrate binding to the PLK1 PBD with an apparent IC_50_ of only 41 μM using an FP assay, less than that of Poloppin, and is also active against PLK2 PDB with an IC_50_ of 105 μM (Figure S3D). Although the hydrophobicity of the compounds has precluded validation of their binding modes using X-ray crystallography, two possible explanations may account for the disconnect between their apparent potencies in biochemical versus cellular assays. First, switching from an acid (Poloppin) to an amine (Poloppin-II) may alter cell permeability or retention. Second, recent data (Zhu et al., 2016) suggest that the PBD domain assumes ordered dimeric conformations in the cellular milieu to regulate PLK1 activity, raising the possibility that the relevant target conformer in cells is distinct from the recombinant PBD proteins used in the FP assay. Nevertheless, we cannot exclude entirely the possibility that Poloppin-II acts via targets additional to the PLK PBD.

To further corroborate Poloppin-II's cellular mechanism of action, we used stable isotope labeling using amino acids in culture coupled to mass spectrometry (see STAR Methods) to compare the patterns of changes induced in the human phosphoproteome after mitotic arrest triggered by Poloppin-II with the spindle poison, Nocodazole, or with the ATP-competitive PLK1 inhibitor, Volasertib (Figure 5E). The abundance of 95 phosphopeptides is decreased ≥2-fold after both Poloppin-II and Nocodazole exposure (yellow box, left-hand plot), of which only one (1.05%) contains the PLK1 phosphorylation consensus motifs (D/E)-X-(S/T)-(Φ), (Φ)-(D/E)-X-(S/T)-(Φ), and (Φ)-X-(D/E)-X-(S/T)-(Φ), where Φ is a hydrophobic residue (Oppermann et al., 2012). By contrast, 238 phosphopeptides decrease by ≥2-fold after both Poloppin-II and Volasertib exposure (yellow box, right-hand plot), of which 42 (17.65%) contain consensus PLK1 motifs. These findings suggest that Volasertib and Poloppin-II, but not Nocodazole, preferentially inhibit the phosphorylation of a common set of cellular proteins containing consensus motifs for PLK1-dependent phosphorylation. Since phosphopeptide engagement via the PBD is a critical step that directs PLK kinase activity to its substrates (Elia et al., 2003a, Elia et al., 2003b), these data strengthen the evidence supporting Poloppin-II's mechanism of action in cells.

Poloppin-II is inactive against the hERG ion channel, and is stable in human (CL_int_ = 10 μL/min/million cells; T_1/2_ = 136 min) and mouse (CL_int_ = 18 μL/min/million cells; T_1/2_ = 77 min) hepatocytes. Upon oral administration in mice (Figure S3E), Poloppin-II maintains an *in vivo* plasma concentration >100 nM for >24 hr following a single dose of 10 mg/kg. Oral bioavailability exceeds 90%, with a half-life of 15 hr, calculated clearance of 17 mL/min/kg and a volume of distribution of 23 L/kg. These favorable attributes prompted us to test the efficacy of Poloppin-II in an *in vivo* xenograft model using the mutant KRAS G13D-expressing colorectal cancer cell line, HCT116 (Figure 5F). Poloppin-II at 50 mg/kg (Figure S3F) was well tolerated during the study, with no adverse health observations or recorded weight loss (Figure S3G). At this dose, Poloppin-II reduced the growth of xenografted tumors in a statistically significant manner (Figures 5F and S3H). Xenografts exposed to Poloppin-II exhibit increased levels of phospho-Ser10 histone H3 expression within 24 hr compared with vehicle-treated controls (Figure S3I), providing a pharmacodynamic marker of xenograft exposure, and linking reduced tumor growth to mitotic arrest.

### Poloppin Resistance Develops Less Readily than to an ATP-Competitive PLK1 Inhibitor

**Figure 6:**
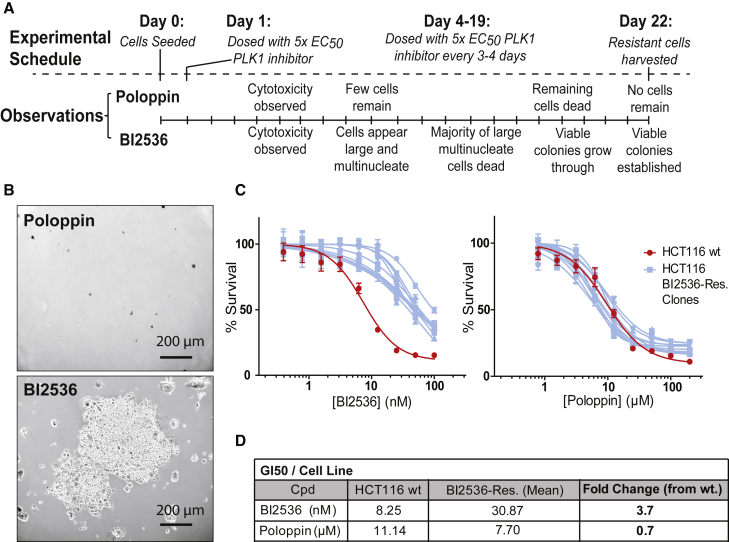
Poloppin Resistance Develops Less Readily than to an ATP-Competitive PLK1 Inhibitor (A) Experimental schedule employed to generate drug-resistant HCT116 KRAS G13D cells; qualitative observations for BI2536 and Poloppin are noted. (B) Representative bright-field images are shown of cultures after continuous BI2536 or Poloppin treatment. (C) Viability of drug-sensitive parental HCT116 cells (HCT116 WT) and BI 2536-resistant clones (HCT116 BI 2536-Res. Clones) treated with BI2536 or Poloppin. (D) The GI_50_ dose for each compound is shown for each cell line. Data represent the mean of three independent experiments ± SEM. See also Figure S4.

Mutant KRAS-expressing HCT116 colorectal carcinoma cells have previously been used to investigate resistance to PLK1 inhibition by the ATP-competitive PLK1 inhibitor, BI2536 (Wacker et al., 2012). When treated with 5 times the half maximal growth inhibition (GI_50_) concentrations of either Poloppin or BI2536 (Figure 6A), BI2536-resistant, but not Poloppin-resistant, colonies are readily observed (Figure 6B). BI2536-resistant cells harbor the known resistance mutation, PLK1 R136G (Wacker et al., 2012) (Figure S4A). Mutations affecting PLK2 or PLK3 were not detected. Interestingly, BI2536-resistant cells retain sensitivity to Poloppin (Figure 6C).

### Poloppin Sensitizes Mutant KRAS-Expressing Cells to Clinically Used Inhibitors of the c-MET Tyrosine Kinase

**Figure 7:**
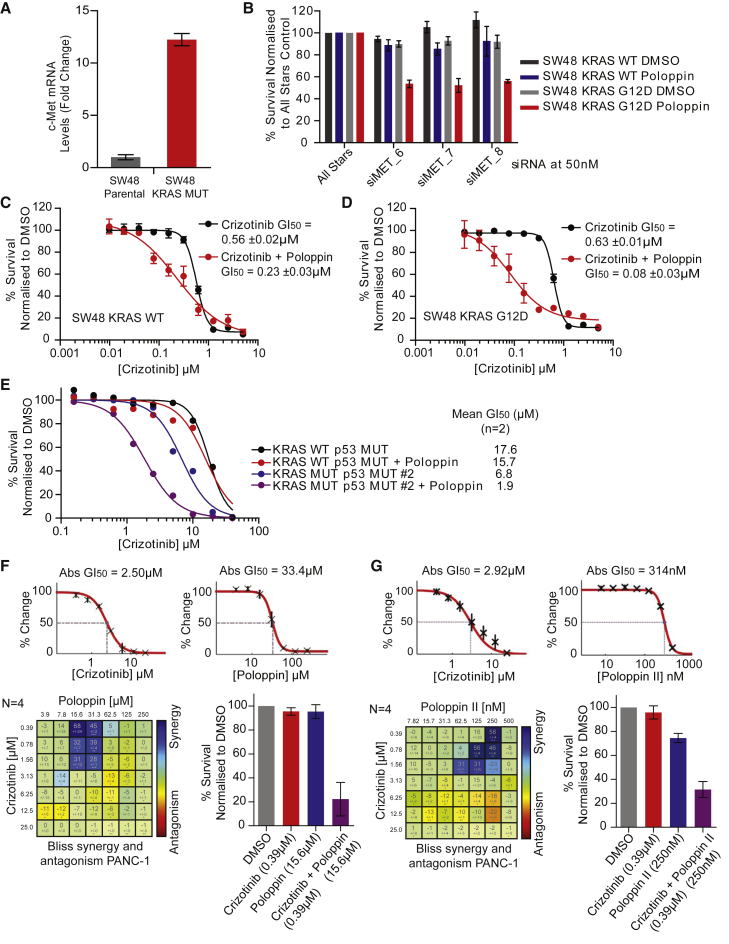
Poloppins Sensitize Mutant KRAS-Expressing Cells to Clinically Used Inhibitors of the c-MET Tyrosine Kinase via a Synergistic Mechanism (A) c-MET transcript is upregulated ∼12-fold in SW48 KRAS G12D cells compared with their isogenic parental counterparts. (B) Viability measured after c-MET was depleted by siRNA in SW48 KRAS wild-type or KRAS-G12D expressing cells and treated with DMSO or Poloppin at GI_25_. Depletion was confirmed by western blots (see Figure S5). (C and D) SW48 KRAS wild-type (C) and SW48 KRAS G12D (D) cells were treated with the c-MET inhibitor, Crizotinib, in combination with either DMSO or Poloppin at GI_25_. Cell viability was measured in an SRB assay after 72 hr treatment. (E) Viability of murine pancreatic organoids treated with Crizotinib plus DMSO (black and blue) or Crizotinib plus Poloppin at GI_25_ (gray and red). (F and G) An analysis of synergy between Crizotinib in combination with Poloppin (F) or Poloppin-II (G) in the Panc-1 cell line expressing mutant KRAS G12D. Line graphs show the percentage change in cell viability after treatment with the individual compounds after 72 hr. The BLISS matrix shows an analysis of a checkerboard titration indicating dose ranges for synergy (indigo) or antagonism (red). Bar graphs show cell viability after 72 hr exposure to optimal synergistic doses of Crizotinib with Poloppin (F) or Crizotinib with Poloppin-II (G), compared with single compound treatment. Results are typical of at least three independent experiments. Data represent the mean of three independent experiments ± SEM unless otherwise stated. See also Figure S5 and Table S1.

The proto-oncogene c-MET, a *bona fide* therapeutic target in human malignancies, including pancreatic cancer (reviewed in Cecchi et al., 2012), is upregulated in KRAS-mutant SW48 cells versus isogenic controls (Figure 7A). c-MET depletion using small interfering RNA (siRNA) (Figure S5A) selectively increased sensitization to Poloppin at the GI_25_ dose in mutant KRAS-expressing SW48 cells but not their parental counterparts (Figure 7B; charcoal and red bars). This effect does not occur with Poloppin alone (black and blue bars). In addition, several different cancer cell lines bearing mutant oncogenic KRAS were sensitized by 2- to 4-fold to Poloppin after depletion of c-MET using siRNA (Figure S5B).

We tested several c-MET inhibitors, including Crizotinib (Xalkori), an FDA-approved compound (Rodig and Shapiro, 2010), for late-stage non-small-cell lung cancers that express the abnormal anaplastic lymphoma kinase. SW48 KRAS-G12D cells are approximately 7-fold more sensitive to Crizotinib in the presence of Poloppin compared with SW48 parental cells (Figures 7C and 7D). A similar but greater effect is also observed in murine pancreatic organoids expressing mutant oncogenic Kras compared with controls (Figure 7E). In addition, several different KRAS-mutant cell lines were sensitized to Crizotinib in the presence of Poloppin (Table S1). Similar effects were apparent using two alternative MET inhibitors, Foretinib (Qian et al., 2009) or Tivatinib (Yap et al., 2011), currently in clinical development (Figure S5C). RNAi depletion of PLK1, but not other PLKs, sensitized mutant *KRAS*-expressing cells to Foretinib, whereas depletion of PLK1–4 induced sensitization to Crizotinib, supporting the proposed mechanism of action (Figure S5D).

Poloppin and Crizotinib are synergistic at doses below both their respective GI_50_ values for cell killing in Panc-1 cells, using the BLISS independence method, which assumes that the agents may act via independent mechanisms (Figure 7F). In a similar manner, Poloppin-II also sensitizes Panc-1 cells to Crizotinib at doses below their respective GI_50_ for cell killing, acting in a synergistic manner (Figure 7G). These results indicate that combining the c-MET inhibitor, Crizotinib, with either Poloppin or Poloppin-II arrests the growth of mutant KRAS-bearing cancer cells with greater efficacy than when the compounds are used independently, and suggest that c-MET inhibition acts synergistically with inhibition of the PLK PBD.

## Discussion

Here, we report the development of Poloppin and Poloppin-II as chemical leads that inhibit phosphopeptide binding by the PBD motifs conserved in the human Polo-like kinases, PLK1–4. Neither compound shares chemical features with several previously reported PBD inhibitors recently reported to be non-specific protein alkylators (Archambault and Normandin, 2017). Moreover, both compounds lack reactive groups likely to induce irreversible inhibition, and do not detectably interact with a panel of 51 structurally or functionally related protein kinases.

Notably, Poloppin and Poloppin-II trigger mitotic arrest with non-congressed chromosomes consistent with blockade of the essential functions of PLK1 PBD during cell division, and distinct from the monopolar spindle phenotype induced by ATP-competitive PLK1 inhibitors. Both compounds also kill cancer cells expressing mutant oncogenic KRAS. Several lines of evidence speak to the mechanism by which Poloppin and Poloppin-II elicit these cellular effects.

Both compounds inhibit phosphopeptide substrate binding to the homologous PBD domains of PLK1 and PLK2 in biochemical assays, and engage PLK1–4 in the cellular milieu when tested by cellular thermal shift. Depletion using RNAi of PLK1 or PLK4 but not PLK2 or PLK3 can recapitulate mitotic arrest and the killing of mutant KRAS-expressing cells. These findings collectively suggest that the Poloppins selectively engage the PBD1–4 target domains, and, furthermore, that suppression of PLK1 and PLK4 underlies their key cellular effects in triggering mitotic arrest, and in killing cancer cells that express mutant oncogenic KRAS. Overexpression of the PLK1 PBD elicits similar cellular effects, further supporting the proposed mechanism of action.

Whereas Poloppin-II is highly potent in inducing these cellular phenotypes, it is perplexingly less active in biochemical assays, raising the possibility that the compound acts via cellular targets additional to the Polo-like kinases. However, like its parental compound Poloppin, Poloppin-II does not bind detectably to a DiscoverX KinomeScan panel, comprising 51 human protein kinases that exhibit structural or functional similarity with PLK1. Moreover, mass spectrometric analyses indicate that Volasertib (an ATP-competitive PLK1 inhibitor) and Poloppin-II, but not Nocodazole (a mitotic spindle poison), preferentially decrease the phosphorylation of cellular proteins containing consensus motifs for PLK1-dependent phosphorylation. These observations discount the likelihood that Poloppin-II exerts its observed cellular effects via additional targets.

Mutant KRAS expression is proposed to trigger abnormalities in progression through mitosis (“mitotic stress”) that render cancer cells sensitive to PLK1 inhibition (Luo et al., 2009). Consistent with this proposal, induction of mutant KRAS G12D expression in HeLa cells suffices to delay the completion of mitosis (Perera and Venkitaraman, 2016), although it does not by itself significantly increase cell death. Similarly, Poloppin treatment of both wild-type and mutant KRAS G12D-expressing cells also delays mitosis; however, Poloppin treatment preferentially sensitizes mutant KRAS but not wild-type cells to death in mitosis. Thus, our results suggest that the inhibition of substrate recognition by the PBD of PLKs cooperates with mutant oncogenic KRAS expression to trigger death in mitosis.

The *in vivo* bioavailability and favorable pharmacokinetic properties of Poloppin-II, when coupled to its low nanomolar potency in cellular assays, enable for the first time an analysis of tolerability and efficacy by systemic oral administration in an *in vivo* xenograft model, representing an advance over previously reported PBD inhibitors suitable only for intra-tumor injection (Yuan et al., 2011). Poloppin-II has a modest, ∼4- to 5-fold, selectivity for the killing of cells expressing mutant KRAS over wild-type cells. Nevertheless, the compound is relatively well tolerated in mice, and exhibits *in vivo* activity against mutant KRAS-expressing xenografts, with concomitant mitotic arrest within the tumor, suggesting that its therapeutic index for the treatment of mutant KRAS-expressing cancers may exceed that observed in cellular assays.

Cancer therapy using ATP-competitive kinase inhibitors frequently triggers drug resistance via point mutations in the active site (Van den Bossche et al., 2016, Wacker et al., 2012). Interestingly, resistance to Poloppin is undetectable in mutant KRAS-expressing HCT116 cells, which, however, readily acquire resistance to an ATP-competitive PLK1 inhibitor, BI2536, through their mutator phenotype. This finding may reflect that Poloppin inhibits both PLK1 and PLK4 to kill mutant KRAS-expressing cells. BI2536-resistant cells remain sensitive to Poloppin, suggesting the potential utility of PBD-inhibitory compounds in overcoming drug resistance to ATP-competitive PLK1 inhibitors.

Notably, Poloppin and Poloppin-II sensitize mutant KRAS-expressing cancer cells to inhibition of the tyrosine kinase receptor c-MET. Since c-MET inhibitors are already in clinical development for the treatment of cancers of the lung, pancreas, or other organs in which KRAS mutations are also frequent, our findings suggest further interesting opportunities for combination therapy.

## Significance

**Mutations activating the KRAS oncogene drive human cancers affecting the pancreas, lung, colon, and other tissues. We report here that Poloppin and Poloppin-II, compounds that target the protein-protein interactions of human Polo-like kinases, suppress the growth of KRAS-mutant cancer cells as single agents, or in combination with c-MET inhibitors. The cellular phenotypes evoked by Poloppins speak to a mechanism of action that exploits mitotic vulnerabilities triggered by mutant KRAS expression, via the interruption of phosphopeptide substrate recognition by the Polo-box domains of PLK1 or PLK4. Structure-activity relationships defined by the features of Poloppin-II, a potent analog with favorable pharmacokinetics active *in vivo* against KRAS-mutant xenografts, indicate avenues for further development. Poloppin is less susceptible to *in vitro* drug resistance compared with an ATP-competitive PLK1 inhibitor, and retains activity against cells resistant to the ATP-competitive compound, suggesting methods to combat drug resistance. Poloppin and Poloppin-II act synergistically with Crizotinib, a clinically used inhibitor of the c-MET receptor, against mutant KRAS-expressing cancer cells, revealing an approach for combination therapy. Thus, we implicate the protein-protein interactions of PLK1 and PLK4 in the maintenance of mutant KRAS-expressing cancers, and illustrate the use of compounds that block these interactions in single-agent or combination therapies. Our findings endorse the future potential of strategies to target substrate recognition by human protein kinases for drug development.**

## STAR★Methods

### Key Resources Table

**Table:** 

REAGENT or RESOURCE	SOURCE	IDENTIFIER
**Antibodies**		

anti- PLK1	Invitrogen	33-1700
anti-phospho-histone H3 (Ser10)	Abcam	ab5176
Hoechst 33342	Invitrogen	H3570
anti-GAPDH	Santa Cruz	sc137179
anti-alpha tubulin	Santa Cruz	sc32293
anti-CREST	Europa	90C-CS1058
anti-c-MET	Cell Signalling Technology	8198

**Chemicals, Peptides, and Recombinant Proteins**		

Poloppin	this study	
Poloppin II	this study	
PLK1-PBD_345-603_	this study	
PLK2-PBD_356-681_	this study	
TAMRA-MAGPMQTS(pThreonine)PKNGAKK	Designer Bioscience	
BI 2536	SelleckChem	S1109
Nocodazole	Sigma	M1404
Superscript III	Life Technologies	18080044
Rnase H digest	New England Biolabs	M0297S
Amplitaq Gold Polymerase	Applied Biosystems	N8080241
Acetic acid	VWR	20104.334
Sulforhodamine B sodium salt	Sigma	S1402-5G
Trizma base	Sigma	T6066
Trichloroacetic acid solution	Sigma	T0699-100ml

**Critical Commercial Assays**		

Rneasy Mini Kit	Qiagen	74104
Nextera XT Kit	Cambridge Genomic Services	
CellTitre Glo 3D assay	Promega	

**Experimental Models: Cell Lines**		

HeLa Parental (Flp-In T-Rex)	Tighe et al., 2004	
HeLa KRAS G12D	Perera and Venkitaraman, 2016	
HeLa PLK1-PBD_326-603_	this study	
HeLa	ATCC	
U2OS	ATCC	
Panc-1	ATCC	
HPAF II	ATCC	
DLD-1	ATCC	
SW1116	ATCC	
HT29	ATCC	
Panc 02.03	ATCC	
Panc 05.04	ATCC	
SW48 Parental (KRAS WT)	Horizon Discovery	
SW48 KRAS G12D	Horizon Discovery	

**Experimental Models: Organisms/Strains**		

Mouse: LSL-Trp53^R172H/+^	Hingorani et al., 2005, Boj et al., 2015	
Mouse: LSL-Kras^G12D/+^	Hingorani et al., 2005, Boj et al., 2015	
Mouse: Pdx-1-Cre	Hingorani et al., 2005, Boj et al., 2015	
Athymic nude mice, male	Harlan (UK)	

**Oligonucleotides**		

Primer: PLK1F 5'-TTGTAACGTTCCCAGCGC		
Primer: PLK1R 5'-GGCACACTGCAGACATGGC		
Primer: PLK2F 5'-TGCTAGTCGGCACCAGAGG		
Primer: PLK2R 5'-TTCGTACCACCACATGTCCA		
Primer: PLK3F 5'-CGCAGCGTAGCAAATCCAG		
Primer: PLK3R 5'-AAAGCTGGTCCCTGATTCCC		
HS_Met_SG QuantiTect Primer Assay	Qiagen	QT00023408
HS_PLK1_SG QuantiTect Primer Assay	Qiagen	QT00049749
HS_PLK2_SG QuantiTect Primer Assay	Qiagen	QT00049406
HS_PLK3_SG QuantiTect Primer Assay	Qiagen	QT00025207
HS_PLK4_SG QuantiTect Primer Assay	Qiagen	QT00029967
HS_Actin_SG QuantiTect Primer Assay	Qiagen	QT01680476
siRNA: c-Met-AACACCCATCCAGAATGTCAT	Qiagen	
siRNA: c-Met-AAGCCAATTTATCAGGAGGTG	Qiagen	
siRNA: c-Met-AAGTATCAGCTTCCCAACTTC	Qiagen	
siRNA: Plk1-CAACGGCAGCGTGCAGATCAA	Qiagen	
siRNA: Plk2-SMARTpool	Dharmacon	
siRNA: Plk3-SMARTpool	Dharmacon	
siRNA: Plk4-AAGGACTTGGTCTTACAACTA	Qiagen	

### Contact for Reagent and Resource Sharing

Further information and requests for resources and reagents should be directed to the Lead Contact, Ashok R. Venkitaraman (arv22@mrc-cu.cam.ac.uk).

### Experimental Model and Subject Details

#### Cell Lines

Doxycycline (DOX)-inducible HeLa KRAS G12D and PLK1-PBD_326-603_ cells were generated using the Flp-In T-REx system (Life Technologies) (Tighe et al., 2004). The human KrasG12D or PLK1-PBD_326-603_ transgene was subcloned into a modified pcDNA5/FRT/TO vector containing a single amino-terminal Myc tag, followed by co-transfection with a plasmid encoding Flp recombinase (pOG44) into HeLa FRT/TO cells (a kind gift from Stephen Taylor, University of Manchester). Stable integrants were then selected with 200 ug/ml hygromycin (Roche) and 4 ug/ml blasticidin (Invivogen). Transgene expression was induced by treatment with 100 ng/ml doxycycline (Sigma). HeLa, U2OS, Panc-1, HPAF II, DLD-1, SW1116, HT29, Panc 02.03 and Panc 05.04, cells were obtained from the American Type Culture Collection (ATCC). HeLa, U2OS and Panc-1 were cultured in DMEM containing 10% (v/v) fetal bovine serum (FBS). Panc 02.03 and Panc 05.04 were cultured in RPMI medium containing 15% FBS and 10 units/ml Insulin. DLD-1, SW1116, HT29 were cultured in RPMI medium containing 10% FBS. The SW48 isogenic cell lines (KRAS wild-type and KRAS^G12D^) were purchased from Horizon Discovery and cultured in RPMI medium containing 10% FBS. All cells were maintained at 37°C with 5% CO_2_.

#### Primary Cell Cultures

KrasWT mouse embryonic fibroblasts (MEFs) were derived from conditional *LSL-Trp53*^*R172H/+*^ embryos. *LSL-Trp53*^*R172H/+*^, *LSL-Kras*^*G12D/+*^ (Hingorani et al., 2005) strains were interbred to obtain *LSL-Kras*^*G12D/+*^*, LSL-Trp53*^*R172H/+*^ Kras^G12D^ embryos for MEF preparation. Briefly, MEFs were derived from E13.5 embryos after decapitation and evisceration. Finely minced tissue was passed through a 16-G needle two or three times before dissociated cells were cultured in DMEM with 10% FBS. Cre-mediated recombination to activate the Kras^G12D^ allele was induced via addition of 500nM 4-OH Tamoxifen to the culture media overnight.

Organoid cultures were prepared from the pancreas of *LSL-Trp53*^*R172H/+*^*, Pdx-1-Cre* (for Kras WT organoids) or *LSL-Trp53*^*R172H/+*^, *LSL-Kras*^*G12D/+*^, *Pdx-1-Cre* (for Kras^G12D^ organoids KPC3760 and KPC26684) strains (Hingorani et al., 2005). Pancreatic ducts were manually picked after tissue mincing and overnight enzymatic digestion with 0.012% (w/v) collagenase XI (Sigma) and 0.012% (w/v) dispase (GIBCO) in DMEM media containing 1% FBS (GIBCO), before seeding in growth-factor-reduced (GFR) Matrigel (BD) (Boj et al., 2015).

#### In Vivo Animal Studies

Male athymic mice were purchased from Harlan (UK) and acclimatised for 7 days prior to study commencement. Animals were housed in IVC cages (5 per cage) with individual mice identified by tail marking. All animals were allowed free access to a standard certified commercial diet and sanitised water during the study. The holding room was maintained under standard conditions: 20-24°C, 40-70% humidity and a 12h light/dark cycle. All experiments were performed under approved ethical and procedural guidelines.

### Method Details

#### Engineering of PLK1-PBD and PLK2-PBD Fusions

A region of the human PLK1 cDNA sequence (residues 345–603) or human PLK2 cDNA sequence (residues 356-681) was amplified by PCR and cloned into a pGEX-6P1 vector (Invitrogen, Carlsbad, CA) to generate recombinant protein in C41 strain *E. coli* (Garcia-Alvarez et al., 2006). Bacteria were disrupted using an Emulsiflex c5 homogenizer (Avestin) and lysates were passed onto a glutathione S-transferase (GST)-Sepharose column in the presence of 50mM Hepes pH 7.5, 200mM NaCl, 1 mM EDTA, 1mM EGTA and 1mM DTT. Bound fractions were cleaved on column at 4°C overnight with PreScission protease (GE healthcare). Purified fractions were polished by gel chromatography using a Sephadex G25 (GE healthcare) column, collected and concentrated. Fractions were tested by Western blotting using PLK1 antibody (Invitrogen 33-1700) to validate protein purification.

#### Fluorescence Polarisation Assay

Final concentrations of assay components used in binding assays were as follows: TAMRA-labelled consensus PLK1 PBD binding phosphopeptide, 5-TAMRA-MAGPMQS(pThreonine)PLMGAKK-acid 10nM; PLK1 PBD (aa345-603) 84nM (2.5ng/μl). Assays were carried out in PBS (pH 7.4) plus 0.03% tween. DMSO controls were run alongside all experimental compounds and % inhibition normalised to these controls. Compounds were titrated 2 fold from a top concentration of 250μM giving a maximum final concentration of DMSO in the assay of 0.25%; however the assay can tolerate DMSO up to 5%. Compound and PBD protein were incubated together at 22°C for 1 hour prior to addition of the TAMRA labelled binding phosphopeptide. The total assay volume per well was 45μl. All experiments were performed in NBS black 384-well microtiter plates (Corning). Fluorescence Polarisation (FP) was read using a BMG PheraStar plate reader with a 540/590/590nm FP module and unbound 10nm TAMRA-labelled peptide set to a FP value of 35mP.

For FP assays using PLK2 PBD (aa356-681), the same procedure was applied except that TAMRA-MAGPMQTS(pThreonine)PKNGAKK was used as the consensus binding phosphopeptide.

For PLK1 PBD FP high-throughput screen the assay components were as above, compounds were screened at an approximate final concentration of 125μM and final DMSO concentration of 1%. Appropriate volumes of TAMRA-labelled phosphopeptide and Plk1 PBD at 3X final concentration were made up in PBS + 0.1% tween and PBS + 1mM DTT respectively and placed in reservoirs on the Beckman Coulter Biomek FX^p^ Automated Workstation deck in the positions indicated by the scheduling software. Compound dilution plates, black 384 well NBS assay plates and tip racks were loaded into the Cytomat as indicated by the scheduling software. The appropriate Fluorescence Polarisation program was initiated on the BMG PheraStar plate reader and was manually set using an assay plate and 10nm TAMRA-labelled peptide to a FP value of 35mP. All labware was checked to ensure that it is correctly positioned before the scheduling software was enabled and the HTS run was initialised. Test compounds were diluted to 3X final concentration in the dilution plates. 15μl of diluted compound was added into each well of the assay plate followed by 15μl of PBD protein and 15μl of TAMRA-phosphopeptide. Plates were incubated at room temperature for between 20 and 40 min on the robot’s deck or in the Cytomat before the Fluorescence Polarisation was read using the BMG PheraStar plate reader. Test compounds were assayed in triplicate. Mean % inhibition normalised to DMSO control (1%) was calculated for each compound.

#### Isothermal Titration Calorimetry

In all titrations, PBDaa345-603 protein was used at 25 μM and buffered in HEPES (50 mM, pH 7.4), NaCl (200 mM), and DTT (1mM). Protein was diluted into buffer with DMSO at 5% (v/v). Poloppin was prepared by diluting from DMSO stock into the same buffer containing DMSO at 5% (v/v). Great care was taken to match the concentration of DMSO in the ligand and protein samples as closely as possible. In a typical experiment, protein (25 μM) was loaded in the sample cell, and a total of 20 injections of 8μL were made at 2 min intervals from a 200 μL syringe rotating at 1000 rpm and loaded with ligand solution (0.5 mM). In all titrations, an initial injection of 2 μL ligand was made, and these data were discarded during data analysis. The thermodynamic parameters were obtained by fitting the data to a single-site binding model with a stoichiometry of 1. ITC experiments were performed at 20 °C with a MicroCal ITC200 instrument, and all data were analysed with the software implemented in Origin (version 7) (Scott et al., 2015).

#### Mitotic Index Assay

Cells were plated at 10 000/well in 96-well plates and incubated overnight. The following day compound stocks in DMSO were diluted in medium then added to cells with a maximum final DMSO concentration on cells of 0.2%. Cells were incubated with compound for 24 h then fixed in 3.7% formaldeyde. Cells were permeabilised with 0.1% Triton X-100 then incubated with anti-phospho-histone H3 (Ser10) antibody (Abcam ab5176). The cells were washed with PBS then incubated with AlexaFluor 488 labelled goat anti-rabbit IgG (Invitrogen A11034) in the presence of 4ug/ml Hoechst 33342 (Invitrogen H3570). Cells were washed in PBS then imaged on an Arrayscan VTi HCS instrument (Thermo Fisher) using the Target Activation V4 Bioapplication. A user-defined threshold was applied to identify mitotic cells based on the intensity of phospho-histone H3 staining.

#### Cellular Thermal Shift Assay (CeTSA)

Two million HeLa cells were assessed for viability using Trypan blue and aliquoted into PCR tubes for lysate preparation and CeTSA. Aggregation temperature (Tagg) was determined for PLK1 PBD with a Biorad Tetrad gradient maker, using a final concentration of 1mM or 0.5mM for Poloppin or Poloppin-II, respectively, in 5% DMSO (Jafari et al., 2014). Samples were assessed using PLK1 antibodies (Invitrogen 33-1700) and GAPDH (Santa Cruz sc137179) and Li-Cor antibody (926-32252). Quantification was done with Odyssey Li-Cor technology.

#### NanoLuc® Thermal Shift Assay

HeLa cells were transfected with plasmids encoding NanoLuc fused to the N-termini of full-length human PLK1, PLK2, PLK3, PLK4 or p38α MAPK. Twenty-four h after transfection, the thermal stability of the fusion proteins was tested using a modified version of the assay protocol supplied by Promega. Briefly, cells were harvested by tyrpsinisation and centrifugation, counted, and resuspended to 200,000 cells/ml in 3mls of serum-free OptiMEM media. Cells were then permeabilised using with 50ug/ml digitonin in DMSO for 5 min and incubated with test compounds at 100uM, plus protease inhibitor cocktail (Promega G6521), in a final concentration of 5% DMSO for 1 h at 37°C. After incubation, 100 μl/well of cells were aliquoted into a 96-well, thin-walled PCR plate and then heated for 3 minutes using a pre-warmed MJ Tetrad DNA Engine set to run a temperature gradient of 42 to 66°C. The plates were then cooled to room temperature for 10 min, and then 80μl of each sample was transferred to a white-walled 96-well Nunc plate before NanoBRET™ NanoGlo Substrate diluted in Opti-MEM was added. NanoLuc enzyme activity was read out within 10 min using a BMG Clariostar plate reader.

#### DiscoverX KINOMEscan Assay

A panel of 55 proteins (comprising 51 human kinases structurally or functionally related to the PLKs, plus the kinase catalytic domains of PLK1, PLK2, PLK3 and PLK4) were selected for testing by DiscoverX using the KINOMEscan assay platform. Compounds were supplied as 40x DMSO stocks and were tested at a final concentration of 50μM for Poloppin and 5μM for Poloppin-II. Binding interactions were reported as percentage of control where lower numbers indicate stronger binding. A cut-off of <35% was applied to report significant binding.

#### Immunofluorescence

Cells were plated on glass coverslips and incubated overnight. Cells were treated with compound diluted in medium for 24hrs. Medium was then removed and cells fixed in 20mM PIPES pH8, 20mM HEPES pH8, 5mM EGTA, 2mM MgCl, 0.2% Triton X-100 and 3.7% formaldehyde. Cells were washed in PBS containing 0.1% Tween-20 then incubated with anti-alpha tubulin antibody (Santa Cruz sc-32293) and anti-CREST antibody (Europa 90C-CS1058). Cells were washed then incubated with Alexa Fluor 568 labelled goat anti-mouse IgG (Invitrogen A11031) or Alexa Fluor 633 labelled goat anti-mouse IgG (Invitrogen A21052) and Alexa Fluor 488 goat anti-human IgG (Invitrogen A11013). Cells were washed and then mounted on glass slides in Vectashield mounting medium containing DAPI.

#### Western Blotting

Total protein was isolated by directly lysing the cells in non-denaturing lysis buffer (20 mM Tris HCl pH 8, 137 mM NaCl, 10% glycerol, 1% Nonidet P-40 (NP-40), 2 mM EDTA). Protein lysates were resolved on SDS-PAGE, transferred onto a Immobilon-P, PVDF membrane (Millipore), and probed with: Plk1 antibody (Invitrogen 33-1700), c-MET antibody (Cell Signalling Technology 8198). Secondary HRP-conjugated antibodies were used and the signal was detected using an Amersham enhanced chemiluminescence system (ECL, GE Healthcare).

#### Viability Assays

SW48 Parental, SW48 G12D, Panc-1, HPAF-II, Panc 02.03 and Panc 05.04 cell lines were seeded overnight at 5000 cells per well in a 96 well plate in recommended media (ATCC). Compounds were added the following day and viability was measured at 72h using the sulforhodamine B (SRB) colorimetric assay as follows. Cells were fixed by replacing media with 1% v/v Trichloroacetic Acid (TCA) (Sigma-Aldrich) at 4°C and incubated for 1h also at 4°C. Wells were washed thoroughly with copious amounts of deionised water before staining with Sulforhodamine B (SRB) (Sigma-Aldrich) solution (0.057% w/v in 1% v/v Acetic Acid (VWR)) for 30 min. Stain was aspirated and wells washed with several changes of 1% v/v Acetic Acid until unbound stain was removed, before air-drying. Bound stain was solubilized with 100μl / well 10mM Tris (pH 8.0) (Trizma Base (121.1) Sigma-Aldrich) with agitation for 10 min. Fluorescence intensity was recorded at Ex540nm / Em590nm using a BMG Pherastar Plus plate reader (Vichai and Kirtikara, 2006).

Pancreatic organoids were extracted and cultured as detailed above (Boj et al., 2015). In brief, pancreatic organoids were seeded at 600 cells per well in a 96 well plate and allowed to grow for 7 days with media replenishment on the 3^rd^ day. Full-grown organoids were then treated with compounds for 72h and viability determined using CellTitre Glo 3D assay (Promega). For viability assays with c-MET siRNA, cells were reverse transfected in a 6 cm dish using Dharmafect 1 (GE Dharmacon) per manufacturer’s instructions. On the following day, cells were trypsinised and plated onto 96 well plates or 6 well plates. The 96 well plates were treated with a dose range of compounds or DMSO, and viability was determined via SRB colorimetric assay. Cells in the 6 well plates were collected at the end of experiment to corroborate knockdown of c-MET by Western blotting.

#### PBD Overexpression in KRAS^G12D^ Cells

HeLa cells engineered to inducibly overexpress Plk1-PBD (HeLaPBD-326) were transfected with mCherry tagged KRAS G12D (HeLaPBD-326 mCherry KRAS G12D). After selection in Puromycin (0.3ug/ml) for one month, the mCherry positive population was sorted by FACS and alongside the parental cells subjected to a viability assay. HeLaPBD-326 and HeLaPBD-326 mCherry KRAS G12D cells were seeded at 2.5x10^3^ cells/well in 96-well plates. The following day cells were treated with a titration of doxycycline. At the end of 72h incubation period SRB colorimetric assay was performed to assess viability.

#### Live Cell Imaging

Five x10^4^ cells were seeded on each well of 4-well coverglass Lab-Tek chambers in a volume of 800 μl. HeLa-G12D cells were induced with Doxycycline (Dox) 24h before seeding and kept in Dox for the rest of the experiment. The day after seeding, DMSO or Poloppin (at the respective GI_50_ for cell killing: 10μM (HeLa) or 5μM (HeLa-KRAS G12D) was added to the wells and live cell imaging was performed using a fully motorised Leica DMI6000 B inverted microscope equipped with Evolve® 512 EMCCD Camera with an exposure time of 98ms. Bright field images were taken with a HCX PL APO 40x/0.85 CORR CS DRY objective every 5 min for duration of 48h.

#### Pharmacokinetic Study

Mice were dosed orally with 10mg/kg Poloppin-II, and blood samples were taken from 3 mice per time point, and frozen until all samples were available. Plasma was prepared from each sample and Poloppin-II levels analysed by a pre-established methodology (UHPLC-TOF MS using electrospray ionisation).

#### Xenograft Study

Tumours were established by sub-cutaneous implantation of 2 x 10^6^ HCT116 cells suspended in 50% Matrigel into the flanks of a cohort of mice. When a sufficient number of mice had developed tumours of approximately 100mm3, they were randomly assigned to three groups: Vehicle, 50mg/kg Poloppin-II or 200mg/kg Poloppin-II, with 10 mice per group. Mice were treated orally according to their treatment group once every three days. Tumour volume was measured three times per week with calipers. Body weight was measured three times per week. In addition to mice assigned to tumour volume efficacy arm of the study, 16 mice with established HCT116 xenografts were assigned to either Vehicle or 200mg/kg oral dosing groups, with 8 mice per group. 24 hours after a single dose, mice were euthanised, tumours removed, snap frozen and 30-50microgram of lysates were analysed by Western blotting on 4-12% Bis-Tris acrylamide gels, and transferred using the Invitrogen iBlot dry system. Phospho-Histone H3 was detected using Abcam antibody ab5176, beta-actin with Sigma antibody a5316. Secondary detection was achieved with the appropriate HRP-conjugated antibodies from Cell Signalling Technology. HRP was visualised with ECL-Plus (GE Healthcare).

*SILAC and mass spectrometry* Samples were processed for phosphoproteomic analysis as follows. In order to generate light, medium and heavy stable isotope-labelled HeLa S3 cells, arginine and lysine free DMEM medium (Dundee Cell Products) was supplemented with 10% dialysed FBS (GIBCO), 200 mg/L L-proline and either L-lysine (Lys0) together with L-arginine (Arg0), L-lysine-^2^H_4_ (Lys4) with L-arginine-U-^13^C_6_ (Arg6) or L-lysine-U-^13^C_6_-^15^N_2_ (Lys8) with L-arginine-U-^13^C_6_-^15^N_4_ (Arg10) at final concentrations of 28 mg/L for the arginine and 146 mg/L for the lysine until fully metabolically labelled. For SILAC experiments, 2 x 15 cm dishes per condition were treated with PLK inhibitors or DMSO and mitotic cells isolated by shake-off. To increase the yield for DMSO treated control cells, media was replaced after initial shake-off and plates returned to the incubator. A second round of shake off was performed after a further 1 hour incubation. Cells were washed in cold PBS, lysed in lysis buffer (100 mM Tris pH 7.6, 2% SDS, 50 mM NaF, 1 x PhosStop [Roche], 1 x mammalian protease inhibitor [Sigma]), quantified and 1.67 mg of each lysate mixed 1:1:1 (Hammond et al., 2015, van den Biggelaar et al., 2014).

Filter-aided sample preparation (FASP) was used to trypsin digest and isolate peptides (Hammond et al., 2015, van den Biggelaar et al., 2014). For proteome analysis, 5% of the material was removed after FASP. Phosphopeptides were fractionated from the remaining 95% using strong cation exchange (Resource S, GE Healthcare), and enriched using TiO_2_ (van den Biggelaar et al., 2014). All samples were cleaned up using C18 stage tips before analysis by LC-MS. Samples were analysed by the University of Warwick Proteomics Research Technology Platform. Samples were fractionated by reversed phase chromatography using an Ultimate 3000 RSLCnano system (Dionex) coupled to a Thermo Orbitrap Fusion (Thermo Scientific). Samples were loaded onto an Acclaim PepMap μ-precolumn cartridge 300 μm i.d. x 5 mm, 5 μm, 100 Å for 8 min at 10 μL/min in 2% acetonitrile, 0.1% trifluoroacetic acid. For proteome the peptides were then eluted onto an Acclaim PepMap RSLC 75 μm i.d. x 50 cm, 2 μm, 100 Å (Thermo Scientific) at 250 nL/min,using a 110 minute gradient of 8-25% acetonitrile in 0.1% formic acid followed by a 22 minute gradient from 25-35% acetonitrile in 0.1% formic acid. For phosphopeptides sample was eluted from the precolumn at 300 nL/min onto an Acclaim PepMap RSLC 75 μm x 25 cm, 2 μm, 100 Å (Thermo Scientific) using a 37 min gradient from 8-25% acetonitrile in 0.1% formic acid, then an 8 min gradient from 25-35% acetonitrile in 0.1% formic acid. Eluting peptides were converted to gas-phase ions by means of electrospray ionization and analysed on a Thermo Orbitrap Fusion (Thermo Scientific). Survey scans of peptide precursors from 375 to 1500 m/z were performed at 240K resolution (at 200 m/z) with a 2x10^5^ or 4x10^5^ ion count target for proteomes or phosphoproteomes, respectively. The maximum injection time was set to 150 ms. Tandem MS was performed by isolation at 1.2 Th using the quadrupole, HCD fragmentation with normalized collision energy of 30, and rapid scan MS analysis in the ion trap. The MS^2^ ion count target was set to 3x10^3^ or 10^4^ for proteomes or phosphoproteomes, respectively. The maximum injection time was 200 ms. Precursors with charge state 2–6 were selected and sampled for MS^2^. Dynamic exclusion duration was set to 60 s for proteomes or 50 s for phosphoproteomes, with a 10 ppm tolerance around the selected precursor and its isotopes. Monoisotopic precursor selection was turned on. The instrument was run in top speed mode.

#### Resistance to PLK1 Inhibitors

HCT116 wild-type cells (Horizon Discovery Ltd.) cultured in McCoy's 5A (Life Technologies #26600) medium supplemented with 10% Foetal Calf Serum (Life Technologies #10270) were treated on three occasions with titrations of the PLK1 ATP-competitive inhibitor BI 2536 (Selleckchem #S1109) or Poloppin in order to determine the 2.5x GI_50_, 3x GI_50_ and 5x GI_50_ values for each compound required to initiate the development of resistance. Cells were subsequently treated with 2.5x GI_50_, 3x GI_50_ and 5x GI_50_ concentrations of either compound, changing media and compound every three to four days until resistant colonies emerged or no viable cells remained. Single cell clones were isolated from cells resistant to 5x GI_50_ BI 2536. Equivalent experiments using 3x GI_50_ or 2.5x GI_50_ doses of Poloppin or BI2536 yielded identical results (data not shown). BI 2536-resistant clones (HCT116 BI 2536-Res. Clones) were treated with BI2536 or Poloppin, alongside drug-sensitive parental HCT116 cells (HCT116 wt), and viability was assessed after 72 h. Viability is represented as percentage survival normalised to DMSO control. For PLK1 mutation analysis resistant clones were enriched for mitotic cells by treatment for 16h with Nocodazole (Sigma M1404) prior to harvesting by scraping and RNA extraction using Qiagen RNeasy Mini Kits (Qiagen: 74104). cDNA was synthesized using Life Technologies First Strand Synthesis protocols with Superscript III (Life Technologies 18080044), RNase H digested (New England Biolabs M0297S) and subject to PCR for the PLK1 following amplicon. Primers were designed to accommodate additional UTR cDNA in order for Nextera XT kits (Cambridge Genomic Services, University Of Cambridge) to append tagging information without subsequent loss of sequence integrity, as follows: PLK1F 5'-TTGTAACGTTCCCAGCGC, PLK1R 5'-GGCACACTGCAGACATGGC, PLK2F 5'-TGCTAGTCGGCACCAGAGG, PLK2R 5'-TTCGTACCACCACATGTCCA, PLK3F 5'-CGCAGCGTAGCAAATCCAG, and PLK3R 5'-AAAGCTGGTCCCTGATTCCC PCR reactions were carried out using Amplitaq Gold polymerase (Applied Biosystems N8080241) with a 25-cycle programme. PCR products were gel-purified (Qiagen 28704) before being mixed in equimolar quantities in the case of each cell line and submitted for sequencing. Prepared samples were submitted for next generation sequencing using MiSeq Nano run with Nextera XT library preparation. Sequencing and subsequent bioinformatics were performed by Cambridge Genomic Services (University Of Cambridge).

#### RT-qPCR Assay

Total RNA from cultured cells was extracted using TRIzol reagent following the manufacturer instructions (Qiagen). Total RNA samples were submitted to Cambridge Genomic Services (CGS) for whole-transcript profiling on the Affymetrix Gene ST plate arrays and subsequent bioinformatics analysis. For relative quantification of gene expression levels and microarray validation experiments, equal amounts of cDNA were synthesized using SuperScript® III Reverse Transcriptase kit (Invitrogen). qPCR was performed using QuantiTect SYBR® Green PCR Kit and cycling conditions used were 94°C for 15 sec, 55°C for 30 sec, and 70°C for 30 sec for 40 cycles. The specificity of the reaction was verified by melt curve analysis. Experiments were performed using StepOne Real-Time PCR Systems (Applied Biosystems). Primers used in this assay are: c-Met (QT00023408), PLK1 (QT00049749), PLK2 (QT00049406), PLK3 (QT00025207), PLK4 (QT00029967) and beta-actin (QT01680476) from Qiagen.

#### siRNA Sensitization

Cells were reverse-transfected with 50nM (SW48 parental, SW48 KRAS G12D and Panc-1) or 25nM (HeLa Flp-In T-REx parental cells) siRNA using Dharmafect (GE Dharmacon) as manufacturer’s recommendations. Where transfected cells were subsequently treated with compounds, the cells were allowed to settle overnight and then treated with compound at the indicated concentration the following day. Viability was assessed after 72 hrs, with the SRB colorimetric assay. Knockdown efficiencies were determined by Western blot or RT-qPCR. Oligos against the following target sequences were used. c-MET (Qiagen): AACACCCATCCAGAATGTCAT, AAGCCAATTTATCAGGAGGTG, AAGTATCAGCTTCCCAACTTC. PLK1 (Qiagen): CAACGGCAGCGTGCAGATCAA. PLK2 (SMARTpool; Dharmacon): ACACAGAAGGAGAACGAUA, CAUCAAUGAGGAUAGGAUA, CUGCCUAGUGUUACUGAUA, ACAAAGUCUACGCCGCAAA. PLK3 (SMARTpool; Dharmacon): GCGAGAAGAUCCUAAAUGA, GCAAGUGGGUUGACUACUC, GCACAUCCGUUGGCCAUCA, GACCUCAAGUUGGGAAAUU. PLK4 (Qiagen): AAGGACTTGGTCTTACAACTA.

### Quantification and Statistical Analysis

For live cell imaging analysis image sequences were viewed using ImageJ software and cell fate was analysed manually. Time spent in mitosis for different groups was compared with a Kruskal-Wallis test and Dunn's multiple comparison correction.

For phosphoproteomics analysis raw MS peak lists were searched against the Uniprot database (downloaded July 2016) using the Andromeda search engine and processed with the MaxQuant software suite (version 1.5.5.1).

Nanoluciferase enzyme activity of PLK1- or PLK4-Nanoluc fusion protein was analysed using GraphPad Prism. Data were normalised as follows: (treatment A RLU value at X°C temp/treatment A RLU value at 42°C temp) x 100. Using the Boltzmann sigmoidal equation, a curve was then fitted to the normalised data. From this non-linear fit, a V_50_ value was generated, which in this context represents the aggregation onset temperature (Tagg), at which 50% of the PLK1- or PLK4-Nanoluc fusion protein has aggregated.

Bliss model independence synergy was performed as follows: 96-well plates were treated with a dilution series of each drug in an 8 × 8 checkerboard pattern of combinations (Lin et al., 2012). After SRB staining to obtain the growth inhibition data, we used Combenefit software to identify synergistic drug combinations, focusing on BLISS independence (Di Veroli et al., 2016). The single-agent inhibition values were used to calculate a drug combination surface under the assumption of an additive effect. Regions of synergy were then detected by comparing obtained data from a combination with the calculated additive effect (derived by subtracting the calculated additive inhibition values from the measured inhibition to obtain the final difference values). In the final synergy surface, positive values hence indicate synergy regions, whereas negative difference values identify antagonistic effects.

## Author Contributions

B.H. and M.R.T. screened a library designed by D.J.H. to identify Poloppin. A.J.N. assisted in screening and characterized Poloppin in biochemical assays. D.W.W., R.B., and R.G.B. conceived and supervised synthetic chemistry for Poloppin-II development. A.J.N. and S.B. characterized the cellular effects of Poloppin and Poloppin-II with A.E., A.C., and E.G.A. S.P.B. and G.J.M. performed NanoLuc thermal shift assays. G.J.M. and C.J.T. supervised xenograft experiments. A.C. and A.E. performed resistance assays. D.P. provided HeLa KRAS G12D cells and assisted in time-lapse imaging. R.A. assisted in organoid derivation. F.E.H. and I.A.P. performed phosphoproteomic analyses. A.J.N., S.B., A.E., B.H., A.C., G.J.M., and A.R.V. collated data, prepared figures, and wrote the manuscript. A.R.V. conceived the project, and supervised it with G.J.M.

## Supplemental Information

Document S1. Figures S1–S5 and Table S1

Document S2. Article plus Supplemental Information
