# Supplementary material for: Modulating Protein-Protein Interactions of the Mitotic Polo-like Kinases to Target Mutant KRAS
Source: Cell Chem Biol. 2017 Aug 17;24(8):1017–1028.e7. doi: 10.1016/j.chembiol.2017.07.009 (PMC5563081; doi:10.1016/j.chembiol.2017.07.009)
Supplement: Document S1. Figures S1–S5 and Table S1 [file mmc1.pdf]

Cell Chemical Biology, Volume *24*

Supplemental Information

Modulating Protein-Protein Interactions of the Mitotic Polo-like Kinases to Target Mutant KRAS

Ana J. Narvaez, Suzan Ber, Alex Crooks, Amy Emery, Bryn Hardwick, Estrella Guarino Almeida, David J. Huggins, David Perera, Meredith Roberts-Thomson, Roberta Azzarelli, Fiona E. Hood, Ian A. Prior, David W. Walker, Richard Boyce, Robert G. Boyle, Samuel P. Barker, Christopher J. Torrance, Grahame J. McKenzie, and Ashok R. Venkitaraman

SUPPLEMENTAL INFORMATION

Figure S1. Related to Figure 1. Poloppin is a drug-like inhibitor of phosphopeptide binding by PLK1 PBD. (A) Schematic representation of the fluorescence polarisation (FP) assay for inhibitors of substrate recognition by the PLK1 PBD. (B) Determination of the Z Factor. Fluorescence polarisation values for the TAMRA-labelled PLK1 PBD-binding Consensus phosphopeptide bound to PLK1 PBD ((wells 1–576) mean FP value = 128.5mP (SD = 5.2)) compared to unbound ((wells 577–1152) mean FP value = 32.4mP (SD = 2.0)) are shown. Z Factor was calculated using the equation Z Factor = 1 − (3xSD bound + 3xSD unbound)/(mP bound − mP unbound), where SD is the standard deviation from the mean, and mP is the mean fluorescence polarisation. The Z Factor value in this representative experiment was 0.774. (C) Validation of FP assay using phosphopeptide substrates. The ability of unlabelled peptide sequences representing either the phosphorylated (marked P-) or non-phosphorylated forms of known PLK1 PBD substrates (e.g. CDC25C, PBIP1) to compete for binding of a TAMRA-labelled consensus phosphopeptide (TAMRA-MAGPMQS(**pThr)**PLMGAKK) was compared. Unlabelled forms of the consensus peptide itself were also used as positive (P-Consensus) or negative (Consensus) controls. (D) DiscoverX KINOMEscan assay results for Poloppin (50µM). Binding interactions to a panel of 55 kinases were reported as percentage of control where a hit is identified as <35%. (E) Activity of Poloppin against PLK2 PBD in the FP assay. Poloppin was titrated against the TAMRA-labelled PLK2 PBD consensus phosphopeptide (TAMRA-MAGPMQTS(**pThr**)PKNGAKK) bound to PLK2 PBD. The compound shows approximately 3-fold selectivity for PLK1-PBD over PLK2-PBD. Data represent the mean of three independent experiments ± SEM.

FIGURE S1

A

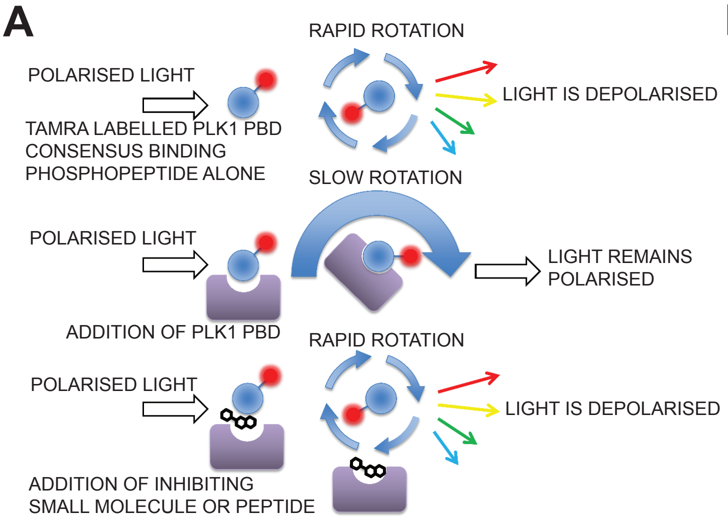

B

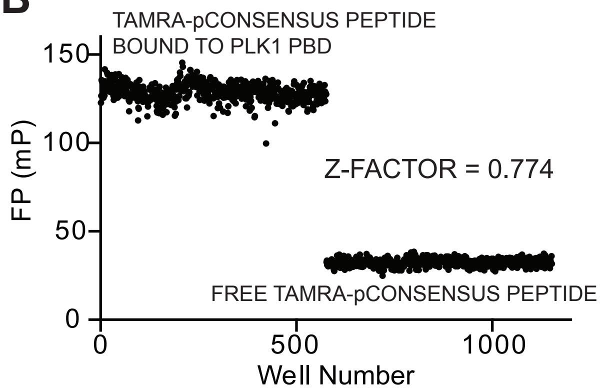

C

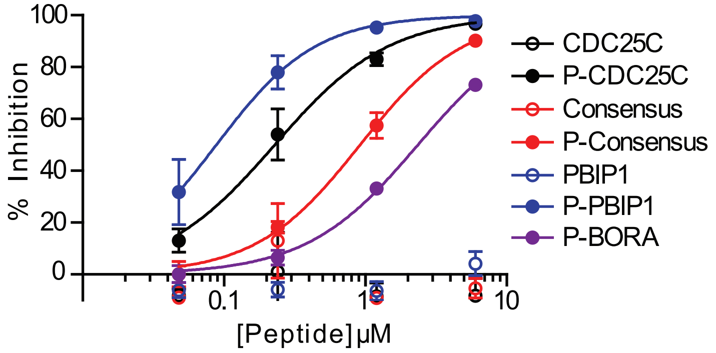

D

Cutoff= 35% Control

Gene Symbol	% Control	Gene Symbol	% Control
AAK1	92	NEK9	95
AURKA	81	PKMYT1	98
AURKB	87	PLK1	100
AURKC	81	PLK2	78
BMP2K	68	PLK3	83
BUB1	99	PLK4	88
CAMKK1	77	EIF2AK2	69
CAMKK2	82	DSTYK	49
CDK2	84	ROCK2	100
CSNK2A1	87	SBK1	61
CSNK2A2	84	SgK110	94
EIF2AK1	97	STK16	81
ERN1	76	STK35	100
GAK	100	STK36	91
EIF2AK4	100	TBK1	75
GSG2	100	TLK1	100
CHUK	100	TLK2	90
IKBKB	100	TTK	85
IKBKE	55	ULK1	84
NEK1	57	ULK2	100
NEK10	98	ULK3	94
NEK11	100	WEE1	99
NEK2	95	WEE2	86
NEK3	72	WNK1	100
NEK4	100	WNK2	100
NEK5	83	WNK3	100
NEK6	96	WNK4	100
NEK7	68		

E

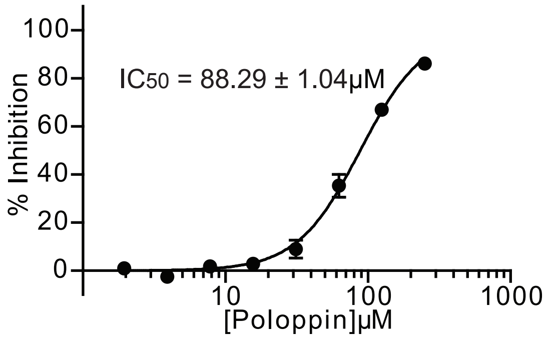

Figure S2. Related to Figure 3. Poloppin exerts its cellular effects via the PBD domains of PLKs.
NanoLuc thermal shift assay for NanoLuc-p38αMAPK fusion protein. Thermal stability curves are shown in the presence of DMSO, the p38α inhibitor AMG548 (at 100nM) and Poloppin or Poloppin-II (at 100μM)

FIGURE S2

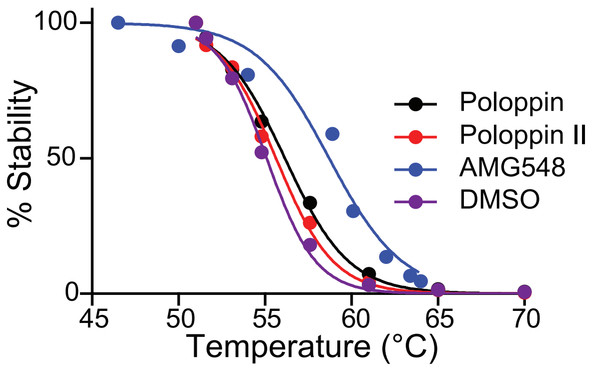

100
50
0
% Stability
45
50
55
60
65
70
Temperature (°C)
Poloppin
Poloppin II
AMG548
DMSO

Figure S3. Related to Figure 5. The optimised analog Poloppin-II is effective by systemic oral administration *in vivo* against mutant KRAS-expressing xenografts. (A) DiscoverX KINOMEscan assay results for Poloppin-II (5µM). Binding interactions to a panel of 55 kinases were reported as percentage of control where a hit is identified as <35%. (B) Quantitation of mitotic phenotypes in cells treated with an EC_{50} concentration of Poloppin-II. Cells were treated as in Figure 3D. (C) Nanoluciferase CeTSA for PLK1 and PLK4. Cells were treated with 100µM Poloppin-II or PB114 over the indicated temperature range. (D) Activity of Poloppin-II against PLK1-PBD and PLK2-PBD in the FP assay. (E) Pharmacokinetic analysis of Poloppin-II, following plasma levels after a single dose of 10mg/kg administered orally. Plasma was sampled at 10 time points (n=3 mice per time point, terminal sampling) over 24 h. Poloppin-II exhibits an oral bioavailability in excess of 90%, a per oral half-life of 15 h, and a volume of distribution of 23 L/kg. (F) Dosing schedule for Poloppin-II. Mice were dosed every three days either with 50mg/kg and 200mg/kg of Poloppin-II, or vehicle as control. Treatment with 200mg/kg Poloppin-II was suspended after seven days. (G) Body weight changes for mice at different doses. Change in body weight during dosing time represented as bodyweight change normalized to pre-treatment levels. (H) Statistical analysis of Poloppin-II treated animals (2 way ANOVA). Percentage of treatment to control (T/C) show shrinkage in tumour volume of 53.3% or 47.7% after 15 days of treatment with 50mg/kg and 200mg/kg respectively. (I) Intra-tumoral effect of Poloppin-II assessed in 8 individual mice by Western blot analysis for phospho-histone H3 phosphorylation on Ser10 in tumor lysates 24 h after treatment at the indicated dose. Where IC_{50}, EC_{50} or GI_{50} values are shown, values are the mean of three independent experiments ± SEM unless otherwise stated.

FIGURE S3

A

Cutoff Value = 35% Control

Gene Symbol	% Control	Gene Symbol	% Control
AAK1	100	NEK9	92
AURKA	83	PKMYT1	100
AURKB	95	PLK1	100
AURKC	85	PLK2	85
BMP2K	85	PLK3	74
BUB1	90	PLK4	90
CAMKK1	91	EIF2AK2	90
CAMKK2	97	DSTYK	75
CDK2	89	ROCK2	100
CSNK2A1	96	SBK1	61
CSNK2A2	92	SgK110	100
EIF2AK1	98	STK16	92
ERN1	74	STK35	71
GAK	100	STK36	91
EIF2AK4	97	TBK1	80
GSG2	77	TLK1	99
CHUK	100	TLK2	94
IKBKB	100	TTK	83
IKBKE	75	ULK1	84
NEK1	100	ULK2	100
NEK10	94	ULK3	88
NEK11	100	WEE1	98
NEK2	94	WEE2	92
NEK3	74	WNK1	97
NEK4	100	WNK2	100
NEK5	99	WNK3	100
NEK6	99	WNK4	91
NEK7	79		

B

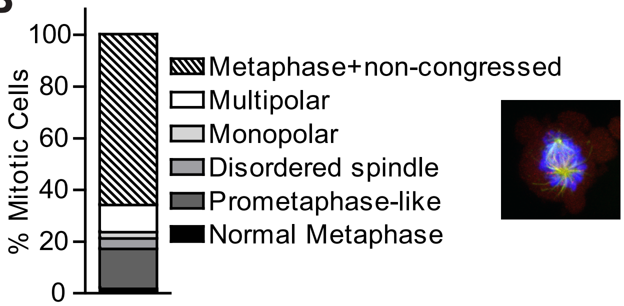

C

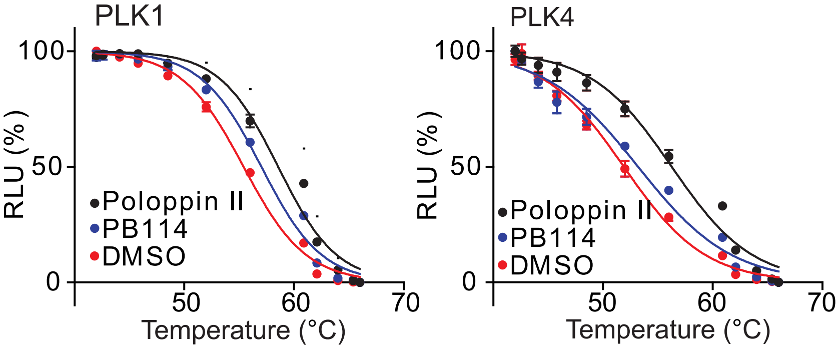

D

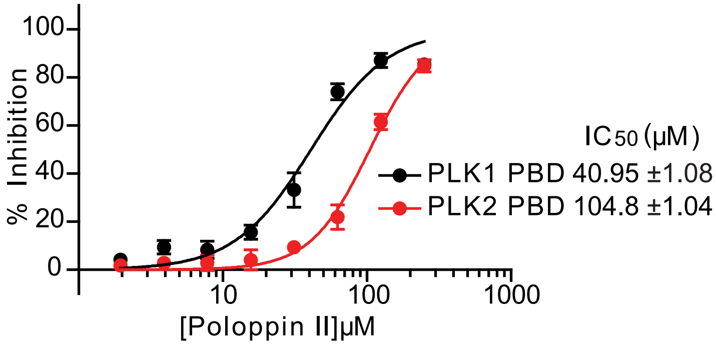

E

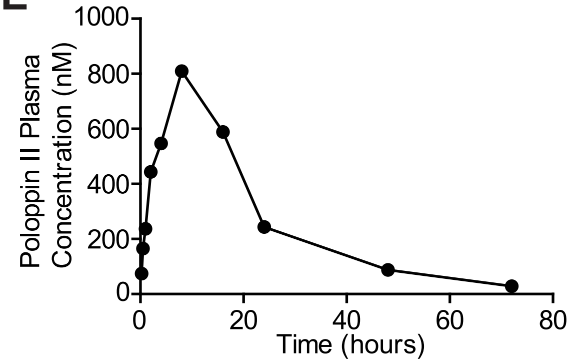

F

Treatment group	Day 1	Day 4	Day 7	Day 10	Day 13
Vehicle	✓	✓	✓	✓	✓
Poloppin II 50mg/kg	✓	✓	✓	✓	✓
Poloppin II 200mg/kg	✓	✓	✓		

G

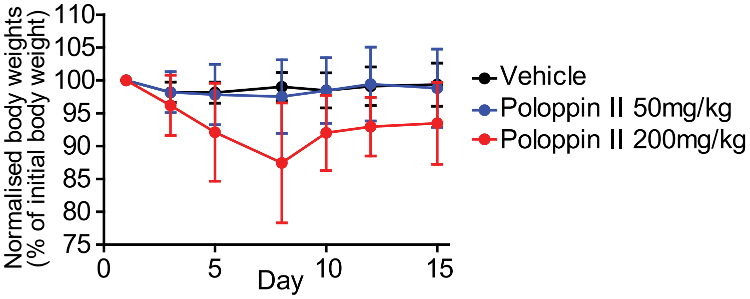

H

Treatment	Group Mean at Day 15	SD	Versus Vehicle (p-value)	Sig	%T/C value
Vehicle	778	172	-	-	-
Poloppin II 50mg/kg	415	159	<0.0001	****	53.3
Poloppin II 200mg/kg	371	125	<0.0001	****	47.7

I

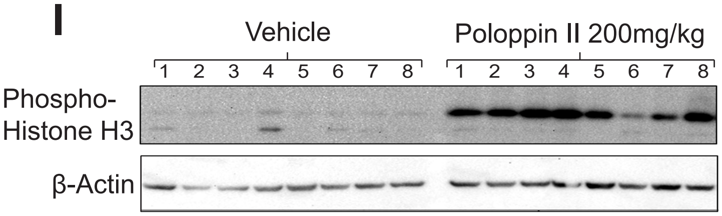

Figure S4. Related to Figure 6. Poloppin resistance develops less readily than to an ATP-competitive PLK1 inhibitor. cDNA extracted from drug-sensitive HCT116 wild-type or HCT116 BI 2536-resistant clones was amplified for PLK1, PLK2 and PLK3 sequences before next-generation sequencing (Illumina), before comparison against reference sequences. The D16G mutation found in wild-type HCT116 cells appears to be a germline mutation, whereas the R136G mutation in BI 2536-resistant clones conforms with a previously observed 'gatekeeper mutation' at the ATP-binding site that confers PLK1-inhibitor resistance (Wacker et al., 2012).

FIGURE S4

Summary Of Mutations In PLK1i-Resistant Cell Lines Analysed By NGS

	Significant Base Variants								Corresponding Coding Muta ons							
	PLK1		PLK2				PLK3		PLK1		PLK2				PLK3	
HCT116wt	A158G		T640C	T1168C	G1201A	T1849C	A1373G	G1595C	D16G		-	-	-	-	-	-
BI 2536-Resistant #1	A158G	A517G	T640C	T1168C	G1201A	T1849C	A1373G	G1595C	D16G	R136G	-	-	-	-	-	-
BI 2536-Resistant #2	A158G	A517G	T640C	T1168C	G1201A	T1849C	A1373G	G1595C	D16G	R136G	-	-	-	-	-	-
BI 2536-Resistant #3	A158G	A517G	T640C	T1168C	G1201A	T1849C	A1373G	G1595C	D16G	R136G	-	-	-	-	-	-
BI 2536-Resistant #4	A158G	A517G	T640C	T1168C	G1201A	T1849C	A1373G	G1595C	D16G	R136G	-	-	-	-	-	-
BI 2536-Resistant #5	A158G	A517G	T640C	T1168C	G1201A	T1849C	A1373G	G1595C	D16G	R136G	-	-	-	-	-	-
BI 2536-Resistant #6	A158G	A517G	T640C	T1168C	G1201A	T1849C	A1373G	G1595C	D16G	R136G	-	-	-	-	-	-
BI 2536-Resistant #7	A158G	A517G	T640C	T1168C	G1201A	T1849C	A1373G	G1595C	D16G	R136G	-	-	-	-	-	-
BI 2536-Resistant #8	A158G	A517G	T640C	T1168C	G1201A	T1849C	A1373G	G1595C	D16G	R136G	-	-	-	-	-	-
BI 2536-Resistant #9	A158G	A517G	T640C	T1168C	G1201A	T1849C	A1373G	G1595C	D16G	R136G	-	-	-	-	-	-

Figure S5. Related to Figure 7. Poloppins sensitize mutant KRAS-expressing cells to clinically used inhibitors of the c-MET tyrosine kinase via a synergistic mechanism. (A) Efficiency of c-MET depletion by siRNA after 96 h assessed by Western blot in SW48 Parental and SW48 KRAS G12D cell lines. (B) Increased cell death induced by Poloppin in human pancreatic cancer cell lines expressing KRAS G12D (Panc-1, HPAF-II, Panc 02.03 and Panc 05.04) after depletion of c-MET and 72 h compound exposure. Non-targeting All Stars siRNA control (black) is compared to c-MET depletion (red). Efficiency of c-MET depletion using siRNA is assessed by Western blot after 96 h downregulation. (C) Cell viability using combinations of different c-MET inhibitors (Crizotinib, Tivatinib and Foretinib) with Poloppin at GI_{25} in Panc-1 cancer cells that express mutant KRAS G12D. Viability was measured by SRB assay after 72 h exposure. (D) Cell viability in Panc-1 cells following depletion of PLKs by siRNA and 72 h exposure to c-MET inhibitors, Crizotinib and Tivatinib. Data represent the mean of three independent experiments ± SEM.

FIGURE S5

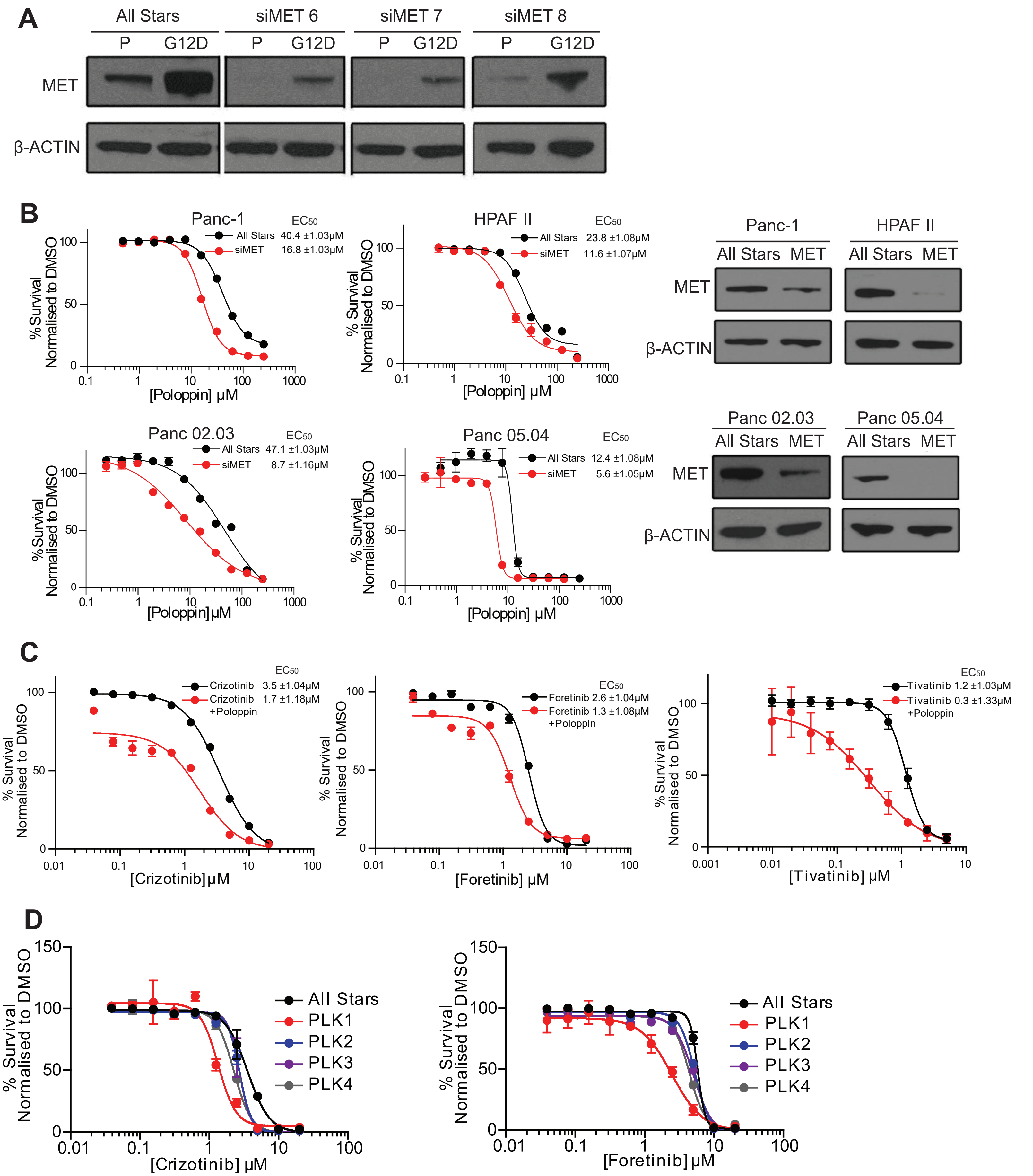

A
All Stars
siMET 6
siMET 7
siMET 8
P
G12D
MET
β-ACTIN
B
Panc-1
EC50
All Stars 40.4 ±1.03μM
siMET 16.8 ±1.03μM
% Survival
Normalised to DMSO
[Poloppin] μM
HPAF II
All Stars 23.8 ±1.08μM
siMET 11.6 ±1.07μM
Panc 02.03
All Stars 47.1 ±1.03μM
siMET 8.7 ±1.16μM
Panc 05.04
All Stars 12.4 ±1.08μM
siMET 5.6 ±1.05μM
All Stars
MET
C
Crizotinib 3.5 ±1.04μM
Crizotinib 1.7 ±1.18μM
+Poloppin
[Crizotinib] μM
Foretinib 2.6 ±1.04μM
Foretinib 1.3 ±1.08μM
+Poloppin
[Foretinib] μM
Tivatinib 1.2 ±1.03μM
Tivatinib 0.3 ±1.33μM
+Poloppin
[Tivatinib] μM
D
All Stars
PLK1
PLK2
PLK3
PLK4

Table S1. Related to Figure 7. Poloppins sensitize mutant KRAS expressing cells to clinically used inhibitors of the c-MET tyrosine kinase via a synergistic mechanism.

Poloppin sensitizes mutant KRAS-expressing human cancer cell lines to c-MET inhibition with Crizotinib

Cell lines	Crizotinib GI_{50} (μM)	Crizotinib GI_{50} (μM) with Poloppin @ GI_{25}
Panc-1	7.24	0.28
HPAFII	N.D.	0.37
Panc 02.03	0.96	0.081
Panc 04.05	0.091	0.037
DLD-1	1.62	0.86
SW116	4.73	2.26
HT29	3.85	2.17

N.D., not determined
