# Supplementary material for: Modulating Protein-Protein Interactions of the Mitotic Polo-like Kinases to Target Mutant KRAS
Source: Cell Chem Biol. 2017 Aug 17;24(8):1017–1028.e7. doi: 10.1016/j.chembiol.2017.07.009 (PMC5563081; doi:10.1016/j.chembiol.2017.07.009)
Supplement: Document S2. Article plus Supplemental Information [file mmc2.pdf]

Article

Cell Chemical Biology

Modulating Protein-Protein Interactions of the Mitotic Polo-like Kinases to Target Mutant KRAS

Graphical Abstract

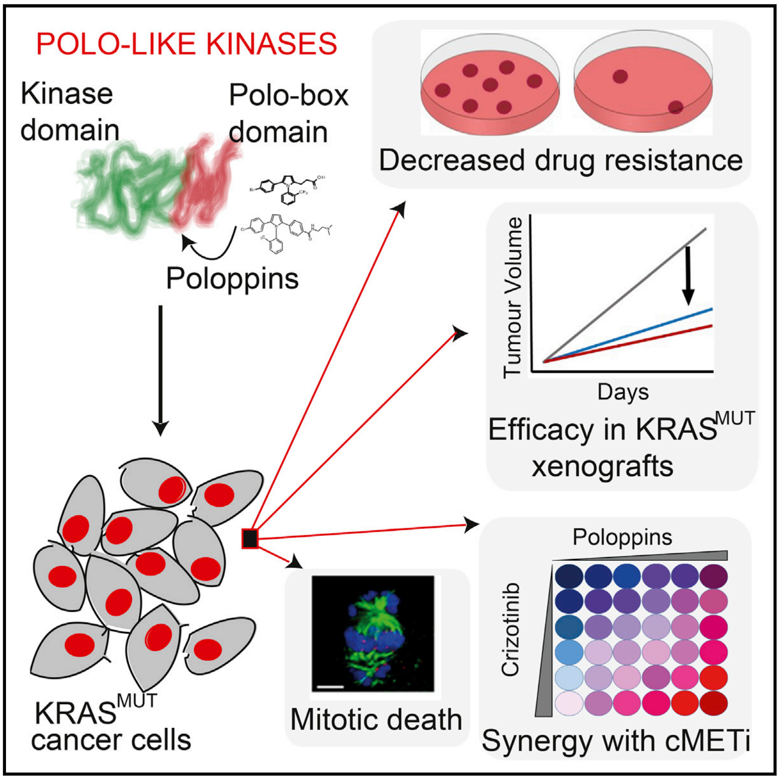

Authors

Ana J. Narvaez, Suzan Ber, Alex Crooks, ..., Christopher J. Torrance, Grahame J. McKenzie, Ashok R. Venkitaraman

Correspondence

arv22@mrc-cu.cam.ac.uk

In Brief

Narvaez et al. report the development of Poloppins, compounds that target the protein-protein interactions of human Polo-like kinases via their Polo-box domain, in monotherapeutic or combination strategies to treat KRAS-mutant cancers.

Highlights

- Poloppins target the PBD of human PLKs causing mitotic death in KRAS-mutant cells
- Depletion of PLK1 or PLK4 recapitulates the cellular effects of Poloppins
- Poloppins reduce drug resistance *in vitro*, and act against KRAS-mutant xenografts
- Poloppins sensitize KRAS-mutant cells to clinically used c-MET inhibitors

Narvaez et al., 2017, Cell Chemical Biology *24*, 1017–1028
August 17, 2017 © 2017 The Authors. Published by Elsevier Ltd.
http://dx.doi.org/10.1016/j.chembiol.2017.07.009

CellPress

Modulating Protein-Protein Interactions of the Mitotic Polo-like Kinases to Target Mutant KRAS

Ana J. Narvaez,[1,9] Suzan Ber,[1] Alex Crooks,[1] Amy Emery,[1] Bryn Hardwick,[1] Estrella Guarino Almeida,[1] David J. Huggins,[1,2,3] David Perera,[1] Meredith Roberts-Thomson,[1] Roberta Azzarelli,[4,5] Fiona E. Hood,[6] Ian A. Prior,[6] David W. Walker,[7] Richard Boyce,[7] Robert G. Boyle,[7] Samuel P. Barker,[8] Christopher J. Torrance,[8] Grahame J. McKenzie,[1,8] and Ashok R. Venkitaraman[1,10,*]

[1]Medical Research Council Cancer Unit, University of Cambridge, Hutchison/MRC Research Centre, Hills Road, Cambridge CB2 0XZ, UK
[2]Department of Chemistry, University of Cambridge, Lensfield Road, Cambridge CB2 1EW, UK
[3]University of Cambridge, Theory of Condensed Matter Group, Cavendish Laboratory, 19 J J Thomson Avenue, Cambridge CB3 0HE, UK
[4]Department of Oncology, University of Cambridge, Hutchison/MRC Research Centre, Hills Road, Cambridge CB2 0XZ, UK
[5]Wellcome Trust-Medical Research Council Cambridge Stem Cell Institute, University of Cambridge, Tennis Court Road, Cambridge CB2 1QR, UK
[6]Division of Cellular and Molecular Physiology, Crown Street, University of Liverpool, Liverpool L69 3BX, UK
[7]Sentinel Oncology Ltd., Cambridge Science Park, Milton Road, Cambridge CB4 0EY, UK
[8]PhoreMost Ltd., Babraham Research Campus, Cambridge CB22 3AT, UK
[9]Present address: Discovery Sciences, Innovative Medicines and Early Development Biotech Unit, AstraZeneca, Darwin Building, Cambridge CB4 0WG, UK
[10]Lead Contact
*Correspondence: arv22@mrc-cu.cam.ac.uk
http://dx.doi.org/10.1016/j.chembiol.2017.07.009

SUMMARY

Mutations activating KRAS underlie many forms of cancer, but are refractory to therapeutic targeting. Here, we develop Poloppin, an inhibitor of protein-protein interactions via the Polo-box domain (PBD) of the mitotic Polo-like kinases (PLKs), in monotherapeutic and combination strategies to target mutant KRAS. Poloppin engages its targets in biochemical and cellular assays, triggering mitotic arrest with defective chromosome congression. Poloppin kills cells expressing mutant KRAS, selectively enhancing death in mitosis. PLK1 or PLK4 depletion recapitulates these cellular effects, as does PBD overexpression, corroborating Poloppin's mechanism of action. An optimized analog with favorable pharmacokinetics, Poloppin-II, is effective against KRAS-expressing cancer xenografts. Poloppin resistance develops less readily than to an ATP-competitive PLK1 inhibitor; moreover, cross-sensitivity persists. Poloppin sensitizes mutant KRAS-expressing cells to clinical inhibitors of c-MET, opening opportunities for combination therapy. Our findings exemplify the utility of small molecules modulating the protein-protein interactions of PLKs to therapeutically target mutant KRAS-expressing cancers.

INTRODUCTION

Human Polo-like kinase 1 (PLK1) regulates multiple events that lead to accurate chromosome segregation during cell division (reviewed in Bruinsma et al., 2012). It comprises an N-terminal kinase catalytic domain, and a C-terminal segment with tandem motifs that form the Polo-box domain (PBD). The PBD engages pSer/pThr phosphopeptide substrates to recruit PLK1 to mitotic structures (Elia et al., 2003a), direct PLK1 kinase activity (Kang et al., 2006), and mediate reaction-diffusion mechanisms for centrosome assembly (Mahen et al., 2011). Homologous PBDs, in which key residues implicated in phosphopeptide substrate recognition are conserved, also occur in the related human Polo-like kinases PLK2, PLK3, and PLK4 (reviewed in Archambault and Glover, 2009).

PLK1's essential role in mitosis, and its dysregulation in several different forms of human cancer, has prompted efforts to create small-molecule inhibitors (reviewed in Liu, 2015). Potent ATP-competitive small-molecule inhibitors of PLK1 kinase activity are in clinical development (reviewed in Gjertsen and Schoffski, 2015). Moreover, compounds that inhibit PBD binding to phosphopeptide substrates are reported to induce mitotic arrest and apoptosis in cancer cell lines at micromolar concentrations (Reindl et al., 2008; Scharow et al., 2015; Watanabe et al., 2009; Yuan et al., 2011); but several have recently been identified as non-specific protein alkylators (Archambault and Normandin, 2017).

Mutations activating the small GTP-binding protein KRAS, which occur frequently in human cancers, have proven largely refractory to therapeutic targeting (reviewed in McCormick, 2015). Inhibitors unique to the G12C KRAS mutant have been described (Ostrem et al., 2013), as have inhibitors of an essential interaction between KRAS and the prenyl-binding protein, PDEδ (Chandra et al., 2012; Zimmermann et al., 2013). An RNAi screen for factors whose depletion inhibits the growth of mutant KRAS G12D-expressing cancer cell lines identified PLK1 and components of the APC/C ubiquitin-conjugating holoenzyme

This is an open access article under the CC BY license (http://creativecommons.org/licenses/by/4.0/).

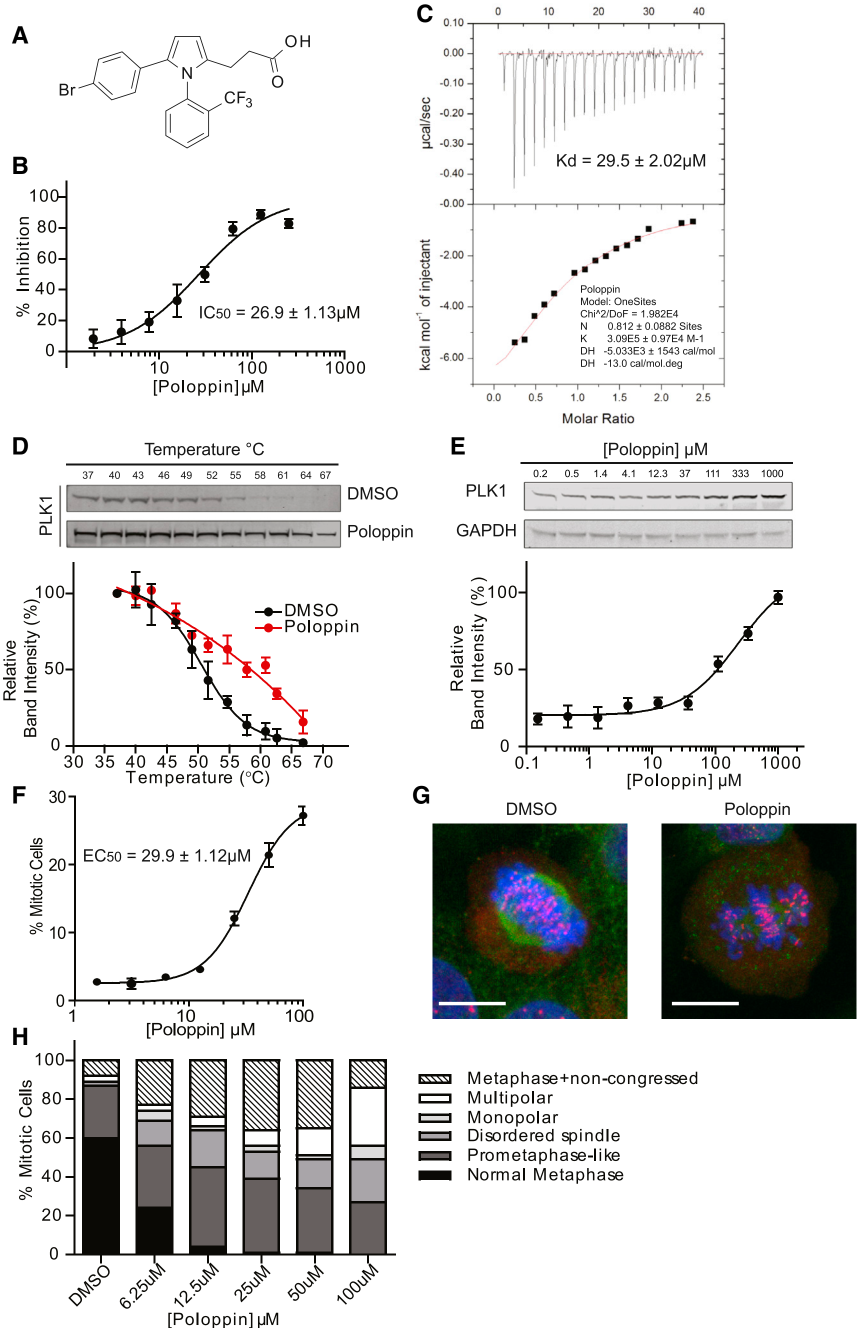

Figure 1. Poloppin Is a Drug-like Inhibitor of Phosphopeptide Binding by PLK1 PBD

(A) Structure of Poloppin.

(B) Poloppin competitively inhibits the binding of a TAMRA-labeled substrate peptide to the PLK1 PBD in an FP assay.

(C) Isothermal titration calorimetry of Poloppin binding to the PBD domain of PLK1.

(D) Poloppin stabilizes PLK1 in a cellular thermal shift assay (CeTSA) in HeLa cell lysates.

(E) $ITDRF_{CETSA}$ at a constant temperature of 67°C in HeLa lysates treated with Poloppin.

(F) Poloppin induces dose-dependent mitotic arrest.

(G) Representative images of U2OS cells treated with Poloppin. Green, tubulin; blue, DNA; red, kinetochores. Scale bars, 15 μm.

(H) Quantification of Poloppin-induced mitotic phenotypes in U2OS cells. The frequency of observed mitotic phenotypes is displayed.

Data represent the mean of three independent experiments ± SEM. See also Figure S1.

(Luo et al., 2009). Genetic modulation of the pathway linking PLK1 to APC/C activation curtailed the proliferation of mutant KRAS G12D-expressing cancer cells, while ATP-competitive PLK1 inhibitors suppressed their growth as xenografts.

Here, we identify compounds that modulate protein-protein interactions of the structurally related PLK kinases via the PBD domain, and demonstrate their utility in strategies to target mutant KRAS. Poloppin (**Polo** **p**rotein-**p**rotein interaction **in**hibitor) kills cells expressing mutant KRAS in two-dimensional or organoid cultures. An optimized analog (Poloppin-II) is effective against KRAS-expressing cancer xenografts after systemic oral administration. Notably, Poloppin sensitivity persists in cancer cells resistant to an ATP-competitive PLK1 inhibitor, and Poloppin sensitizes mutant KRAS-expressing cells to clinically used inhibitors of the MET tyrosine kinase, opening opportunities for combination therapy.

RESULTS

A Drug-like Inhibitor of Phosphopeptide Binding by PLK1 PBD

We screened a diversity library of 120,000 drug-like molecules (Huggins et al., 2011), using a fluorescence polarization (FP) assay (Figures S1A–S1C), to identify compounds that inhibit the binding of the human PLK1 PBD with a tetramethyl-6-carboxy-rhodamine (TAMRA)-labeled PLK1 PBD consensus binding phosphopeptide. Close analogs of the primary hits were selected by Tanimoto similarity using extended-connectivity fingerprints (Xia et al., 2004), before experimental validation. Poloppin (Figure 1A), a 438 Da compound identified in this way, is lipophilic but soluble at up to 1 mM in 5% DMSO, contains no obvious chemical toxicophores or reactive groups, and therefore was regarded as a suitable starting point for development. Poloppin exhibits a half maximal inhibitory concentration (IC_{50}) of 26.9 μM by FP (Figure 1B), while isothermal titration calorimetry reveals that it directly binds to the PLK1 PBD with a K_d of 29.5 μM (Figure 1C).

Poloppin Engages PLK1 to Trigger Mitotic Arrest with Non-congressed Chromosomes

We tested whether Poloppin engages cellular PLK1 using the recently described cellular thermal shift assay (CeTSA). CeTSA

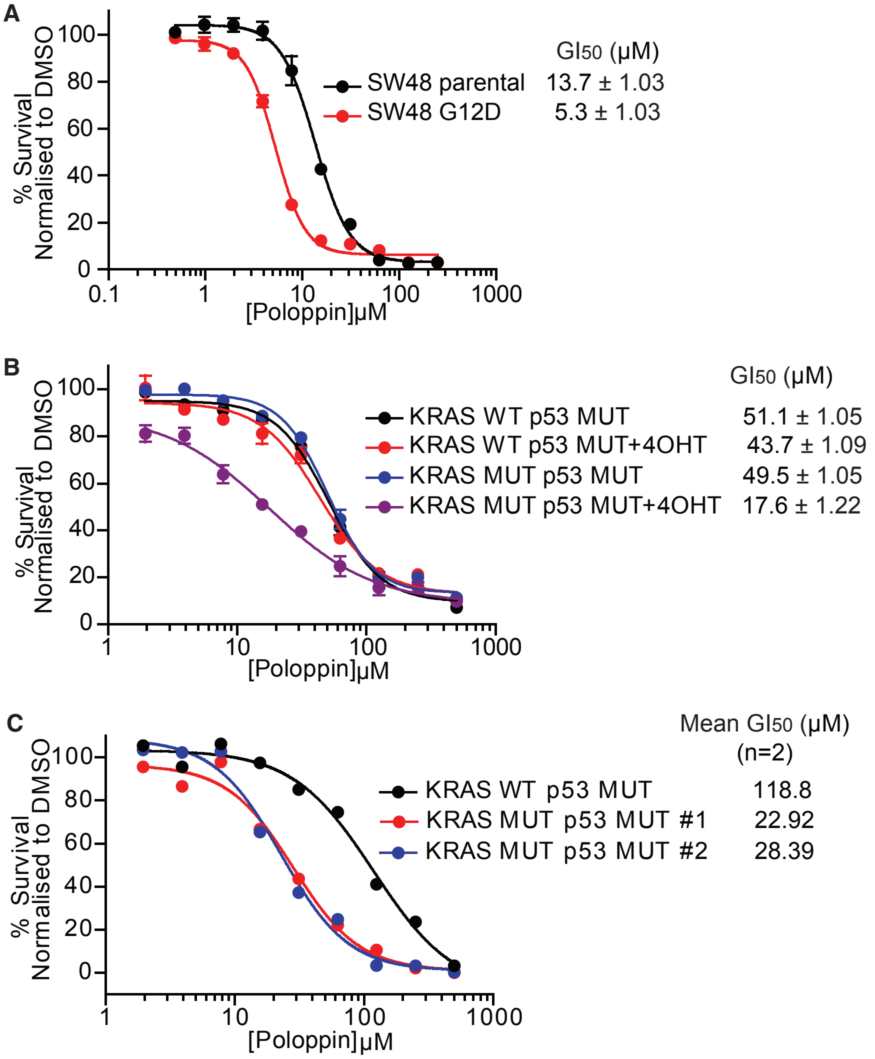

Figure 2. Poloppin Preferentially Kills Cells Expressing Mutant KRAS in Two-Dimensional and Organoid Cultures

(A) Cell viability in SW48 isogenic parental or $KRAS^{G12D}$ cell lines.

(B) Cell viability in MEFs from either a KRAS wild-type, $p53^{R172H}$ (KRAS WT p53 MUT), or a mutant $KRAS^{G12D}$, $p53^{R172H}$ (KRAS MUT p53 MUT) strain.

(C) Viability in murine pancreatic organoids from either a KRAS wild-type, $p53^{R172H}$ (KRAS WT p53 MUT) or mutant $KRAS^{G12D}$, $p53^{R172H}$ (KRAS MUT p53 MUT 1 and 2) strain.

Data represent the mean of three independent experiments ± SEM unless otherwise stated.

detects cellular target engagement via changes in the thermal stability of the drug-bound target, but does not track directly with the potency of compounds in biochemical or phenotypic assays (Jafari et al., 2014; Martinez Molina et al., 2013; Martinez Molina and Nordlund, 2016). PLK1 protein exhibits increased thermal stability following treatment with a fixed dose of 1 mM Poloppin compared with control (Figure 1D). Moreover, exposure of cell lysates at a constant 67°C to increasing doses of Poloppin, using isothermal dose-response fingerprint (ITDRF)-CeTSA, demonstrates that cellular PLK1 is stabilized at >40 μM (Figure 1E). Together, these results provide evidence that Poloppin engages PLK1 in the cellular milieu.

Further evidence comes from the cellular phenotypes induced by Poloppin. Poloppin triggers dose-dependent mitotic arrest in human cell lines (Figure 1F), inducing multiple anomalies in mitosis. At a 12.5 μM concentration, <5% of cells exhibit normal metaphase chromosome alignment, instead arresting with bipolar or disordered spindles and non-congressed chromosomes (Figures 1H and 1G). Poloppin-induced mitotic arrest with non-congressed chromosomes is dissimilar from that induced by ATP-competitive PLK1 inhibitors, which typically suppress bipolar spindle formation (Gilmartin et al., 2009; Hanisch et al., 2006; Lenart et al., 2007).

Collectively, these findings suggest that an early requirement for PLK1 kinase activity during mitotic progression is distinct from the role of substrate engagement by the PBD (Hanisch et al., 2006). Indeed, Poloppin elicits mitotic phenotypes at relatively low concentrations compared with its apparent activity in biochemical assays, consistent with the notion that the compound competitively inhibits PBD binding to functionally relevant substrates present at low cellular abundance, rather than acting as an ATP-competitive catalytic inhibitor.

Poloppin Kills Cells Expressing Mutant KRAS in Two-Dimensional and Organoid Cultures

Mutant KRAS expression sensitizes cells to genetic or pharmacologic modulation of PLK1 (Luo et al., 2009) by ~2-fold over controls. Similarly, SW48 cells expressing KRAS G12D exhibit ~2-fold greater sensitivity to Poloppin than isogenic controls (Figure 2A), as do primary murine embryonic fibroblasts (MEFs) genetically engineered to acutely express mutant KRAS G12D when treated with 4-hydroxy tamoxifen (Figure 2B). Poloppin has a greater (~4- to 5-fold; average, 4.7-fold) inhibitory effect on the growth of pancreatic organoids formed by pancreatic epithelial cells derived from mice genetically engineered to express mutant KRAS G12D in the pancreas (KRAS MUT p53 MUT 1 and 2) compared with KRAS wild-type (KRAS WT p53 MUT) controls (Figure 2C).

Poloppin Exerts its Cellular Effects via the PBD Domains of PLKs

Poloppin does not bind (Figure S1D) to the kinase catalytic domains of either PLK1 or its homologs, PLK2, PLK3, or PLK4, using the DiscoverX KinomeScan assay (Fabian et al., 2005; Karaman et al., 2008). No significant binding was observed to 51 other protein kinases bearing structural or functional similarity to the Polo-like kinases. However, Poloppin inhibits phosphopeptide substrate engagement to the PBD of PLK1

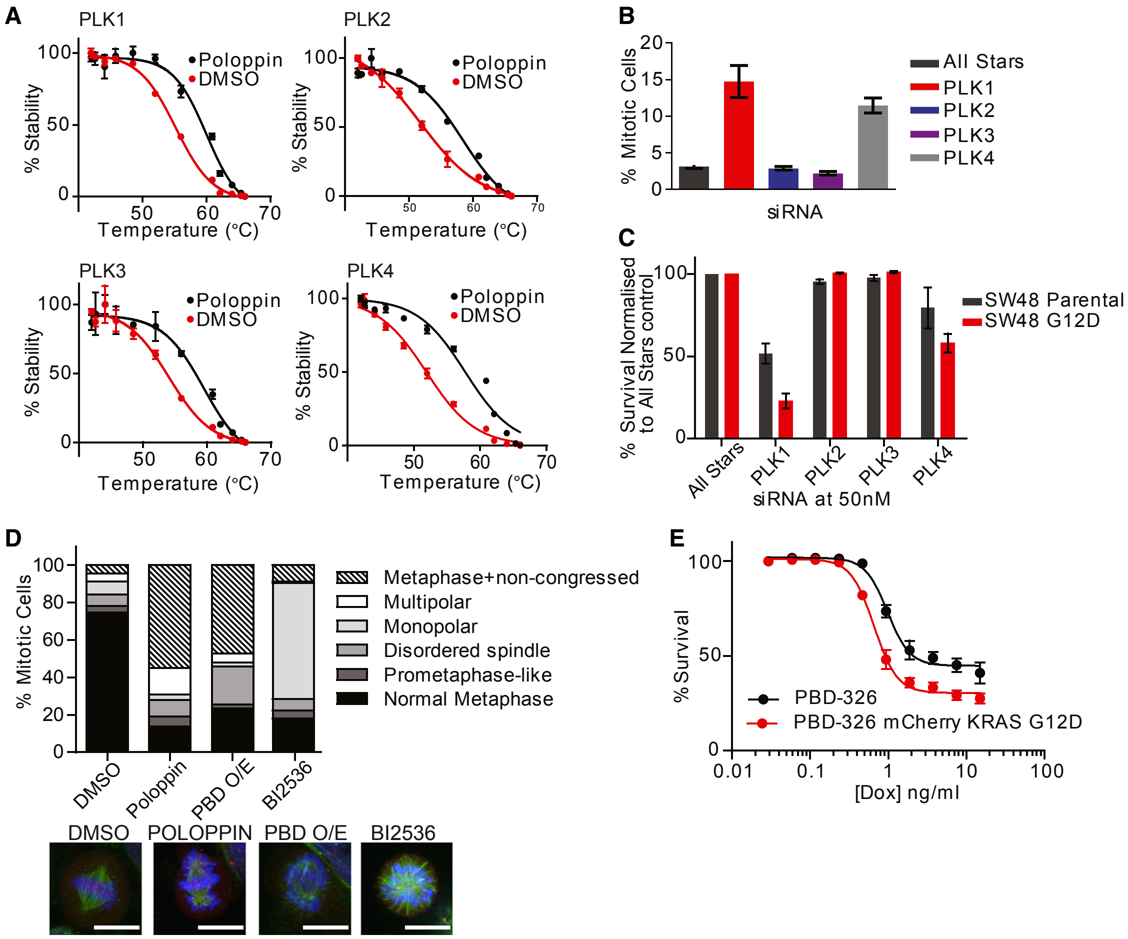

Figure 3. Poloppin Exerts Its Cellular Effects via the PBD Domains of PLKs

(A) Thermal stability assay for PLK1–4 using NanoLuc fusion proteins.

(B) Mitotic index assay in HeLa Flp-In T-REx cells.

(C) Cell viability in the SW48 isogenic parental or $KRAS^{G12D}$ cell lines following depletion of PLKs using indicated siRNAs.

(D) Quantitation of mitotic phenotypes. HeLa cells engineered to overexpress PLK1-PBD upon addition of doxycycline were treated with compounds at EC_{50} concentrations or with doxycycline (PBD O/E) for 16 hr. Mitotic phenotypes were quantified by immunofluorescence imaging. Green, tubulin; blue, DNA; red, kinetochores. Scale bars, 15 μm.

(E) Cell viability of HeLa cells expressing doxycycline-inducible PLK1-PBD, with or without constitutively overexpressed mCherry-KRAS G12D.

Data represent the mean of three independent experiments ± SEM. See also Figure S2.

(Figure 1B; IC_{50} = 29 μM), and with 3-fold less activity (Figure S1E; IC_{50} = 88 μM), to the closely related PBD domain of human PLK2 when tested by FP. Since PLK1–4 all harbor homologous PBD domains (van de Weerdt et al., 2008), we tested whether Poloppin might exert its cellular effects via the inhibition of PLK2, PLK3, or PLK4, besides PLK1.

In cells transiently expressing fusions between PLK1–4 and the reporter Nanoluciferase (NanoLuc) (Hall et al., 2012; Masser et al., 2016), Poloppin at 100 μM increases the thermal stability of all four NanoLuc-PLK fusion proteins (Figure 3A), but not an irrelevant NanoLuc-p38α MAPK fusion protein (Figure S2). These results suggest that Poloppin engages PLK1–4.

We therefore tested whether the selective depletion of PLK1–4 using RNAi could recapitulate Poloppin-induced phenotypes. Depletion of PLK1 or PLK4 but not PLK2 or PLK3 triggers mitotic arrest (Figure 3B). Moreover, PLK1 or PLK4 depletion preferentially decreased the survival of SW48 cells expressing KRAS G12D over that of isogenic control cells (Figure 3C), whereas PLK2 or PLK3 depletion had no such effect. Thus, our findings suggest that PLK1 and PLK4 inhibition underlies the activity of Poloppin in triggering mitotic arrest, and in preferentially killing cancer cells that express mutant oncogenic *KRAS*.

Next, we tested whether inducible overexpression of the PLK1 PBD in cells, which is expected to mimic the effects of Poloppin by competitively inhibiting substrate engagement to the PBD domains, could recapitulate these phenotypic effects. Indeed, cells induced to overexpress the PBD by treatment with doxycycline (Figure 3D) undergo a characteristic metaphase arrest with non-congressed chromosomes, which is typical of the effect induced by Poloppin (Figures 1G and 1H). Inducible PBD expression also preferentially reduces in a dose-dependent manner the viability of cells co-expressing mutant KRAS, over that of the

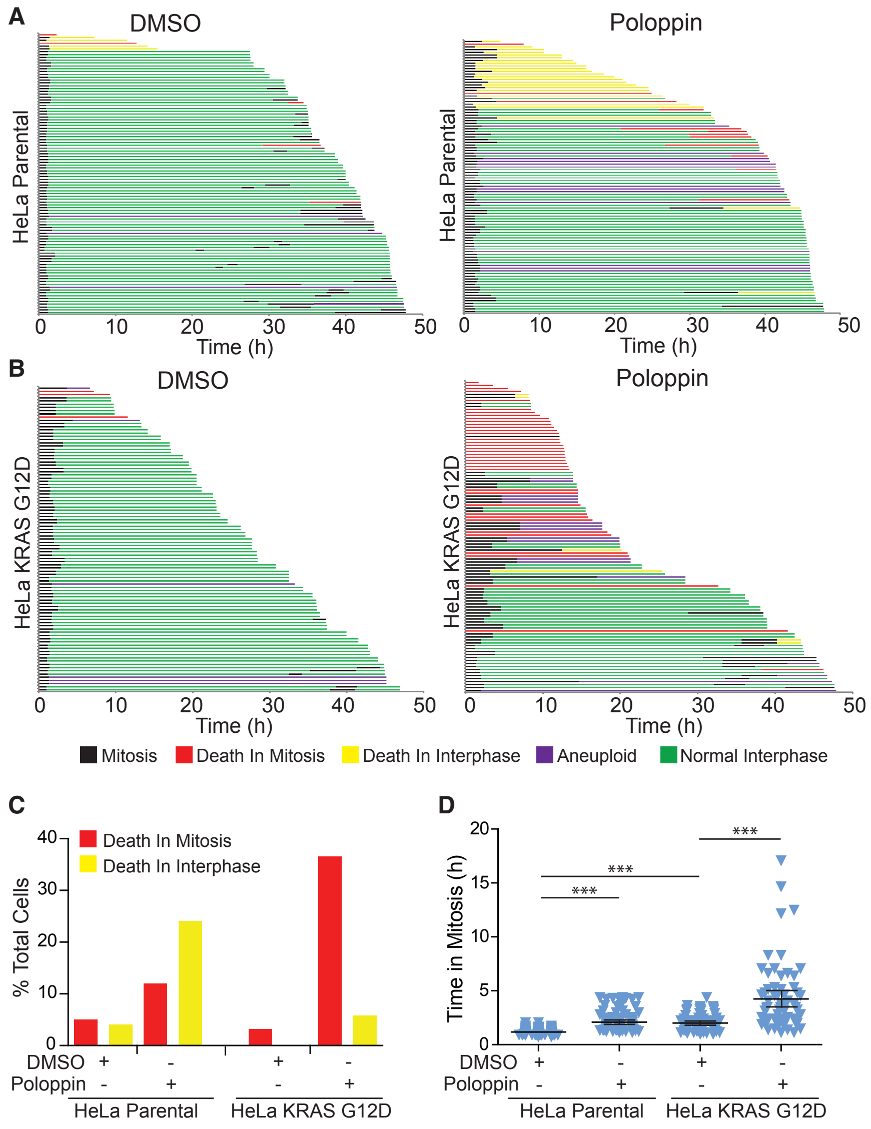

Figure 4. Poloppin Sensitizes Cells Expressing Mutant KRAS to Death in Mitosis

(A and B) Live-cell imaging of (A) parental HeLa cells and (B) HeLa cells expressing $KRAS^{G12D}$ treated with DMSO or with Poloppin at their GI_{50} dose. Each horizontal bar represents the fate of a single cell visualized by serial time-lapse imaging. Colors indicate the different cell fates.

(C) Quantitation of the percentage and type of cell death.

(D) Graph showing time spent in mitosis.

***$p < 0.001$. Error bars represent the 95% confidence interval from the mean.

parental cells (Figure 3E), by ~2-fold, as expected. Thus, collectively, our findings provide genetic evidence to corroborate that the cellular effects of Poloppin are mediated via the PBD domain.

Poloppin Sensitizes Mutant KRAS-Expressing Cells to Death in Mitosis

Using live-cell imaging to track the fate of single wild-type HeLa cells exposed to Poloppin (Figure 4), we find that Poloppin exposure prolongs mitosis from an average of 60 to 100 min (compare black segments in Figures 4A and 4B; tabulated in Figure 4D), and increases the frequency of aneuploid divisions to 38% (purple segments and yellow segments), compared with 4% after DMSO treatment. Typically, Poloppin-treated wild-type cells exit mitosis to form binucleate or anucleate daughters, which undergo death during the subsequent interphase (yellow segments).

By contrast, Poloppin treatment sensitizes mutant KRAS-expressing HeLa cells to death in mitosis. Induction of a chromosomally integrated, doxycycline-inducible KRAS G12D transgene increases the time taken for mitosis from 100 to 200 min (compare black segments in Figure 4A versus 4B; tabulated in Figure 4D), consistent with the notion that mutant KRAS dysregulates mitotic progression as recently reported (Perera and Venkitaraman, 2016). However, when mutant KRAS-expressing HeLa cells are exposed to Poloppin (Figure 4B), there is also a sharp increase in the frequency of death in mitosis (red segments) when compared with DMSO-exposed controls (Figure 4C, 3% versus 36%). Collectively, these results suggest that Poloppin preferentially sensitizes mutant KRAS-expressing cells, but not controls, to death in mitosis.

An Optimized Poloppin Analog Is Effective *In Vivo* against Mutant KRAS-Expressing Xenografts

An optimized synthetic analog, Poloppin-II (Figure 5A), is soluble at up to ~100 μM in 5% DMSO, and exhibits no binding at 5 μM to the kinase catalytic domains of PLK1–4, or to 51 other related kinases using the DiscoverX KinomeScreen assay (Figure S3A). It induces mitotic arrest with non-congressed chromosomes similar to that induced by Poloppin (Figure 5B). Poloppin-II exhibits a half maximal effective concentration of 61 nM in a cellular assay for mitotic arrest compared with 14.6 μM for Poloppin, whereas a structurally related analog of Poloppin-II (PB114) is inactive (Figure 5B). Poloppin-II engages PLK1 and PLK4, as detected using NanoLuc fusion proteins, whereas PB114 is less active (Figure S3C). Poloppin-II sensitizes cells expressing mutant KRAS in two-dimensional or organoid cultures by approximately 5-fold (Figures 5C and 5D).

Despite its potency in cellular assays, Poloppin-II competitively inhibits substrate binding to the PLK1 PBD with an apparent IC_{50} of only 41 μM using an FP assay, less than that of Poloppin, and is also active against PLK2 PDB with an IC_{50} of 105 μM (Figure S3D). Although the hydrophobicity of the compounds has precluded validation of their binding modes using X-ray crystallography, two possible explanations may account for the disconnect between their apparent potencies in

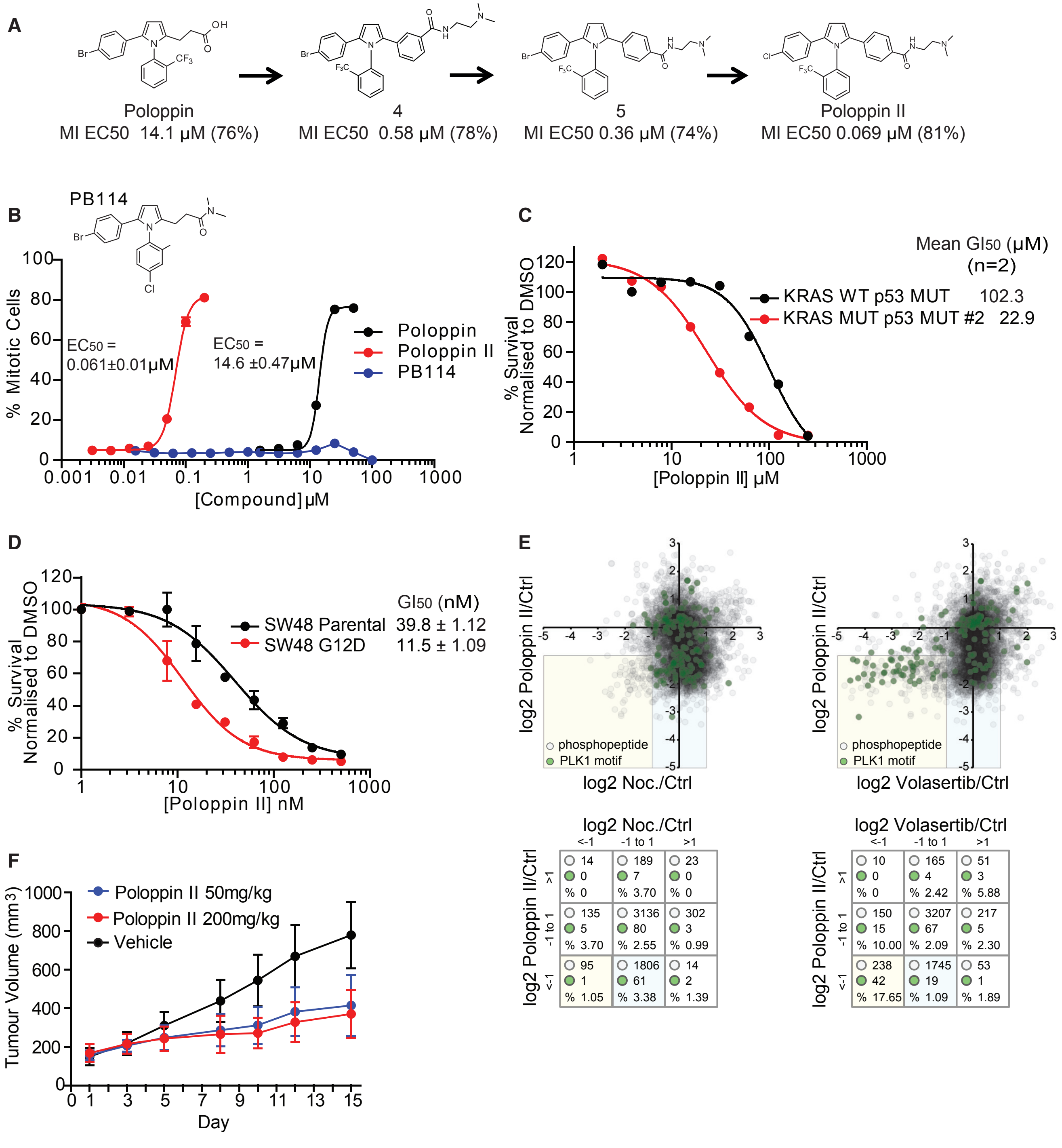

Figure 5. The Optimized Analog Poloppin-II Is Effective by Systemic Oral Administration *In Vivo* Against Mutant KRAS-Expressing Xenografts

(A) Synthetic chemistry route from Poloppin to Poloppin-II. The EC_{50} value of each analog in a cellular assay for mitotic arrest is given below its designation, with the maximum percentage of mitotic cells in brackets.

(B) Mitotic index assay in HeLa cells treated for 16 hr with Poloppin, Poloppin-II, or the structurally related analog, PB114.

(C) Cell viability in KRAS wild-type murine pancreatic organoids (KRAS WT p53 MUT), or organoids expressing KRAS G12D (KRAS MUT p53 MUT).

(D) Cell viability in SW48 parental and KRAS G12D isogenic cell lines at 72 hr. Data represent the mean of three independent experiments ± SEM.

(E) Mass spectrometric analysis of changes in phosphopeptide abundance induced by Poloppin-II versus Nocodazole or the ATP-competitive PLK1 inhibitor, Volasertib. Pairwise comparisons of the relative abundance of phosphopeptides detected in this analysis are plotted logarithmically to the base 2 (top panels). Green dots indicate phosphopeptides that contain the PLK1 phosphorylation consensus motifs. The boxed, yellow-shaded area in the bottom left-hand quadrant marks phosphopeptides that exhibit a $\geq$2-fold reduction in abundance in both conditions. The tables below each dot plot show the total number of

(legend continued on next page)

biochemical versus cellular assays. First, switching from an acid (Poloppin) to an amine (Poloppin-II) may alter cell permeability or retention. Second, recent data (Zhu et al., 2016) suggest that the PBD domain assumes ordered dimeric conformations in the cellular milieu to regulate PLK1 activity, raising the possibility that the relevant target conformer in cells is distinct from the recombinant PBD proteins used in the FP assay. Nevertheless, we cannot exclude entirely the possibility that Poloppin-II acts via targets additional to the PLK PBD.

To further corroborate Poloppin-II's cellular mechanism of action, we used stable isotope labeling using amino acids in culture coupled to mass spectrometry (see STAR Methods) to compare the patterns of changes induced in the human phosphoproteome after mitotic arrest triggered by Poloppin-II with the spindle poison, Nocodazole, or with the ATP-competitive PLK1 inhibitor, Volasertib (Figure 5E). The abundance of 95 phosphopeptides is decreased $\geq$2-fold after both Poloppin-II and Nocodazole exposure (yellow box, left-hand plot), of which only one (1.05%) contains the PLK1 phosphorylation consensus motifs (D/E)-X-(S/T)-(Φ), (Φ)-(D/E)-X-(S/T)-(Φ), and (Φ)-X-(D/E)-X-(S/T)-(Φ), where Φ is a hydrophobic residue (Oppermann et al., 2012). By contrast, 238 phosphopeptides decrease by $\geq$2-fold after both Poloppin-II and Volasertib exposure (yellow box, right-hand plot), of which 42 (17.65%) contain consensus PLK1 motifs. These findings suggest that Volasertib and Poloppin-II, but not Nocodazole, preferentially inhibit the phosphorylation of a common set of cellular proteins containing consensus motifs for PLK1-dependent phosphorylation. Since phosphopeptide engagement via the PBD is a critical step that directs PLK kinase activity to its substrates (Elia et al., 2003a, 2003b), these data strengthen the evidence supporting Poloppin-II's mechanism of action in cells.

Poloppin-II is inactive against the hERG ion channel, and is stable in human (CL_{int} = 10 μL/min/million cells; $T_{1/2}$ = 136 min) and mouse (CL_{int} = 18 μL/min/million cells; $T_{1/2}$ = 77 min) hepatocytes. Upon oral administration in mice (Figure S3E), Poloppin-II maintains an *in vivo* plasma concentration >100 nM for >24 hr following a single dose of 10 mg/kg. Oral bioavailability exceeds 90%, with a half-life of 15 hr, calculated clearance of 17 mL/min/kg and a volume of distribution of 23 L/kg. These favorable attributes prompted us to test the efficacy of Poloppin-II in an *in vivo* xenograft model using the mutant KRAS G13D-expressing colorectal cancer cell line, HCT116 (Figure 5F). Poloppin-II at 50 mg/kg (Figure S3F) was well tolerated during the study, with no adverse health observations or recorded weight loss (Figure S3G). At this dose, Poloppin-II reduced the growth of xenografted tumors in a statistically significant manner (Figures 5F and S3H). Xenografts exposed to Poloppin-II exhibit increased levels of phospho-Ser10 histone H3 expression within 24 hr compared with vehicle-treated controls (Figure S3I), providing a pharmacodynamic marker of xenograft exposure, and linking reduced tumor growth to mitotic arrest.

Poloppin Resistance Develops Less Readily than to an ATP-Competitive PLK1 Inhibitor

Mutant KRAS-expressing HCT116 colorectal carcinoma cells have previously been used to investigate resistance to PLK1 inhibition by the ATP-competitive PLK1 inhibitor, BI2536 (Wacker et al., 2012). When treated with 5 times the half maximal growth inhibition (GI_{50}) concentrations of either Poloppin or BI2536 (Figure 6A), BI2536-resistant, but not Poloppin-resistant, colonies are readily observed (Figure 6B). BI2536-resistant cells harbor the known resistance mutation, PLK1 R136G (Wacker et al., 2012) (Figure S4A). Mutations affecting PLK2 or PLK3 were not detected. Interestingly, BI2536-resistant cells retain sensitivity to Poloppin (Figure 6C).

Poloppin Sensitizes Mutant KRAS-Expressing Cells to Clinically Used Inhibitors of the c-MET Tyrosine Kinase

The proto-oncogene c-MET, a *bona fide* therapeutic target in human malignancies, including pancreatic cancer (reviewed in Cecchi et al., 2012), is upregulated in KRAS-mutant SW48 cells versus isogenic controls (Figure 7A). c-MET depletion using small interfering RNA (siRNA) (Figure S5A) selectively increased sensitization to Poloppin at the GI_{25} dose in mutant KRAS-expressing SW48 cells but not their parental counterparts (Figure 7B; charcoal and red bars). This effect does not occur with Poloppin alone (black and blue bars). In addition, several different cancer cell lines bearing mutant oncogenic KRAS were sensitized by 2- to 4-fold to Poloppin after depletion of c-MET using siRNA (Figure S5B).

We tested several c-MET inhibitors, including Crizotinib (Xalkori), an FDA-approved compound (Rodig and Shapiro, 2010), for late-stage non-small-cell lung cancers that express the abnormal anaplastic lymphoma kinase. SW48 KRAS-G12D cells are approximately 7-fold more sensitive to Crizotinib in the presence of Poloppin compared with SW48 parental cells (Figures 7C and 7D). A similar but greater effect is also observed in murine pancreatic organoids expressing mutant oncogenic Kras compared with controls (Figure 7E). In addition, several different KRAS-mutant cell lines were sensitized to Crizotinib in the presence of Poloppin (Table S1). Similar effects were apparent using two alternative MET inhibitors, Foretinib (Qian et al., 2009) or Tivatinib (Yap et al., 2011), currently in clinical development (Figure S5C). RNAi depletion of PLK1, but not other PLKs, sensitized mutant *KRAS*-expressing cells to Foretinib, whereas depletion of PLK1–4 induced sensitization to Crizotinib, supporting the proposed mechanism of action (Figure S5D).

Poloppin and Crizotinib are synergistic at doses below both their respective GI_{50} values for cell killing in Panc-1 cells, using the BLISS independence method, which assumes that the agents may act via independent mechanisms (Figure 7F). In a similar manner, Poloppin-II also sensitizes Panc-1 cells to Crizotinib at doses below their respective GI_{50} for cell killing, acting in a synergistic manner (Figure 7G). These results indicate that combining the c-MET inhibitor, Crizotinib, with either Poloppin

phosphopeptides, the number of PLK1 motif-containing phosphopeptides, and the percentage of PLK1 motif-containing phosphopeptides in nine different bins defined by (log2) abundance values.

(F) Tumor progression in a xenograft model of HCT116 cells expressing $KRAS^{G13D}$ after systemic treatment via oral administration with Poloppin-II. Error bars indicate mean ± SD.

See also Figure S3.

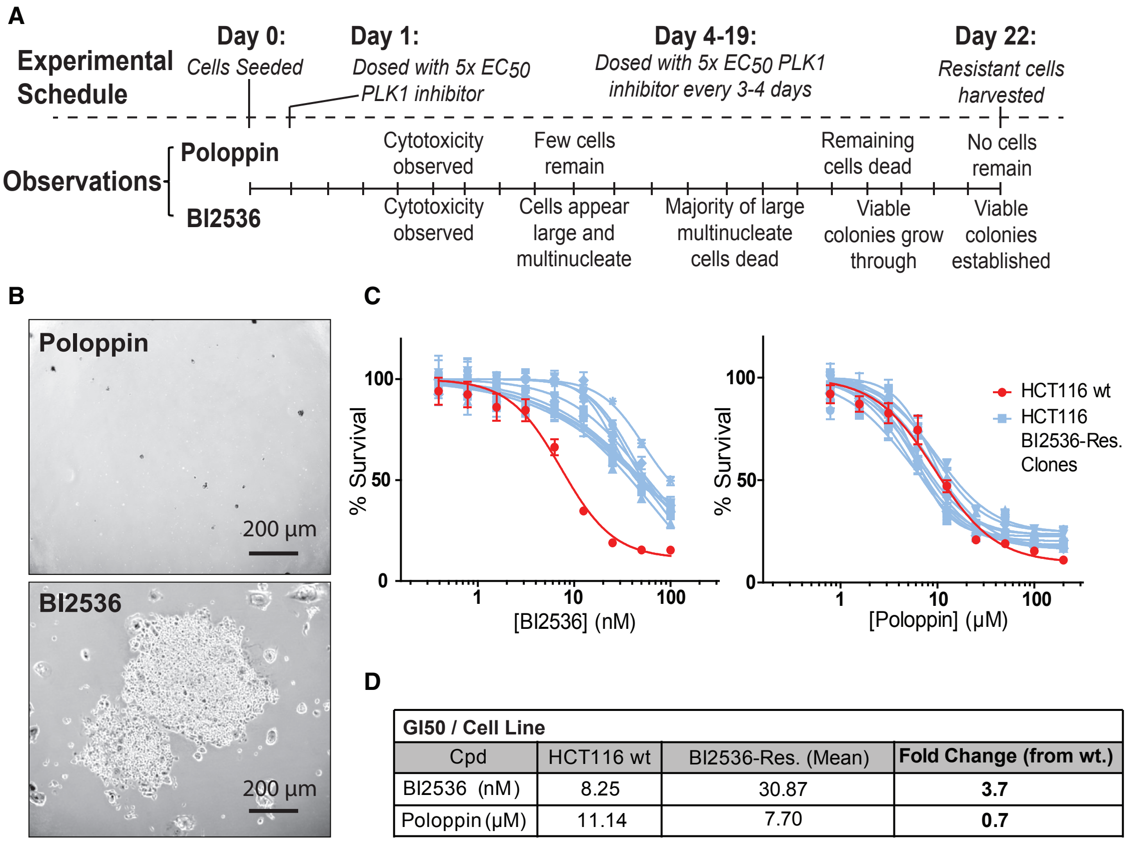

GI50 / Cell Line			
Cpd	HCT116 wt	BI2536-Res. (Mean)	**Fold Change (from wt.)**
BI2536 (nM)	8.25	30.87	**3.7**
Poloppin (µM)	11.14	7.70	**0.7**

Figure 6. Poloppin Resistance Develops Less Readily than to an ATP-Competitive PLK1 Inhibitor

(A) Experimental schedule employed to generate drug-resistant HCT116 KRAS G13D cells; qualitative observations for BI2536 and Poloppin are noted.

(B) Representative bright-field images are shown of cultures after continuous BI2536 or Poloppin treatment.

(C) Viability of drug-sensitive parental HCT116 cells (HCT116 WT) and BI 2536-resistant clones (HCT116 BI 2536-Res. Clones) treated with BI2536 or Poloppin.

(D) The GI_{50} dose for each compound is shown for each cell line.

Data represent the mean of three independent experiments ± SEM. See also Figure S4.

or Poloppin-II arrests the growth of mutant KRAS-bearing cancer cells with greater efficacy than when the compounds are used independently, and suggest that c-MET inhibition acts synergistically with inhibition of the PLK PBD.

DISCUSSION

Here, we report the development of Poloppin and Poloppin-II as chemical leads that inhibit phosphopeptide binding by the PBD motifs conserved in the human Polo-like kinases, PLK1–4. Neither compound shares chemical features with several previously reported PBD inhibitors recently reported to be non-specific protein alkylators (Archambault and Normandin, 2017). Moreover, both compounds lack reactive groups likely to induce irreversible inhibition, and do not detectably interact with a panel of 51 structurally or functionally related protein kinases.

Notably, Poloppin and Poloppin-II trigger mitotic arrest with non-congressed chromosomes consistent with blockade of the essential functions of PLK1 PBD during cell division, and distinct from the monopolar spindle phenotype induced by ATP-competitive PLK1 inhibitors. Both compounds also kill cancer cells expressing mutant oncogenic KRAS. Several lines of evidence speak to the mechanism by which Poloppin and Poloppin-II elicit these cellular effects.

Both compounds inhibit phosphopeptide substrate binding to the homologous PBD domains of PLK1 and PLK2 in biochemical assays, and engage PLK1–4 in the cellular milieu when tested by cellular thermal shift. Depletion using RNAi of PLK1 or PLK4 but not PLK2 or PLK3 can recapitulate mitotic arrest and the killing of mutant KRAS-expressing cells. These findings collectively suggest that the Poloppins selectively engage the PBD1–4 target domains, and, furthermore, that suppression of PLK1 and PLK4 underlies their key cellular effects in triggering mitotic arrest, and in killing cancer cells that express mutant oncogenic KRAS. Overexpression of the PLK1 PBD elicits similar cellular effects, further supporting the proposed mechanism of action.

Whereas Poloppin-II is highly potent in inducing these cellular phenotypes, it is perplexingly less active in biochemical assays, raising the possibility that the compound acts via cellular targets additional to the Polo-like kinases. However, like its parental compound Poloppin, Poloppin-II does not bind detectably to a DiscoverX KinomeScan panel, comprising 51 human protein kinases that exhibit structural or functional similarity with PLK1. Moreover, mass spectrometric analyses indicate that Volasertib

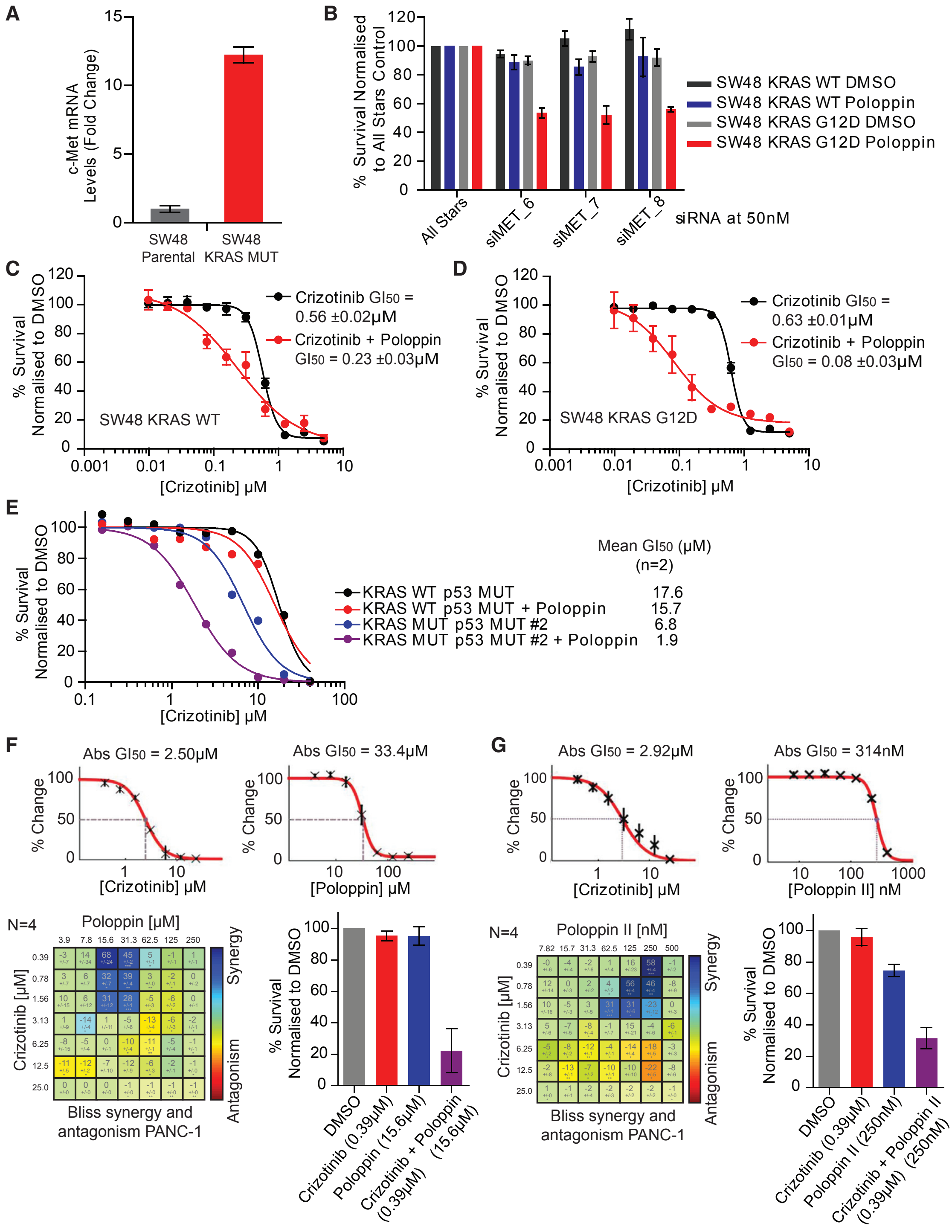

Figure 7. Poloppins Sensitize Mutant KRAS-Expressing Cells to Clinically Used Inhibitors of the c-MET Tyrosine Kinase via a Synergistic Mechanism

(A) c-MET transcript is upregulated ~12-fold in SW48 KRAS G12D cells compared with their isogenic parental counterparts.

(B) Viability measured after c-MET was depleted by siRNA in SW48 KRAS wild-type or KRAS-G12D expressing cells and treated with DMSO or Poloppin at GI_{25}. Depletion was confirmed by western blots (see Figure S5).

(C and D) SW48 KRAS wild-type (C) and SW48 KRAS G12D (D) cells were treated with the c-MET inhibitor, Crizotinib, in combination with either DMSO or Poloppin at GI_{25}. Cell viability was measured in an SRB assay after 72 hr treatment.

(E) Viability of murine pancreatic organoids treated with Crizotinib plus DMSO (black and blue) or Crizotinib plus Poloppin at GI_{25} (gray and red).

(legend continued on next page)

(an ATP-competitive PLK1 inhibitor) and Poloppin-II, but not Nocodazole (a mitotic spindle poison), preferentially decrease the phosphorylation of cellular proteins containing consensus motifs for PLK1-dependent phosphorylation. These observations discount the likelihood that Poloppin-II exerts its observed cellular effects via additional targets.

Mutant KRAS expression is proposed to trigger abnormalities in progression through mitosis ("mitotic stress") that render cancer cells sensitive to PLK1 inhibition (Luo et al., 2009). Consistent with this proposal, induction of mutant KRAS G12D expression in HeLa cells suffices to delay the completion of mitosis (Perera and Venkitaraman, 2016), although it does not by itself significantly increase cell death. Similarly, Poloppin treatment of both wild-type and mutant KRAS G12D-expressing cells also delays mitosis; however, Poloppin treatment preferentially sensitizes mutant KRAS but not wild-type cells to death in mitosis. Thus, our results suggest that the inhibition of substrate recognition by the PBD of PLKs cooperates with mutant oncogenic KRAS expression to trigger death in mitosis.

The *in vivo* bioavailability and favorable pharmacokinetic properties of Poloppin-II, when coupled to its low nanomolar potency in cellular assays, enable for the first time an analysis of tolerability and efficacy by systemic oral administration in an *in vivo* xenograft model, representing an advance over previously reported PBD inhibitors suitable only for intra-tumor injection (Yuan et al., 2011). Poloppin-II has a modest, ~4- to 5-fold, selectivity for the killing of cells expressing mutant KRAS over wild-type cells. Nevertheless, the compound is relatively well tolerated in mice, and exhibits *in vivo* activity against mutant KRAS-expressing xenografts, with concomitant mitotic arrest within the tumor, suggesting that its therapeutic index for the treatment of mutant KRAS-expressing cancers may exceed that observed in cellular assays.

Cancer therapy using ATP-competitive kinase inhibitors frequently triggers drug resistance via point mutations in the active site (Van den Bossche et al., 2016; Wacker et al., 2012). Interestingly, resistance to Poloppin is undetectable in mutant KRAS-expressing HCT116 cells, which, however, readily acquire resistance to an ATP-competitive PLK1 inhibitor, BI2536, through their mutator phenotype. This finding may reflect that Poloppin inhibits both PLK1 and PLK4 to kill mutant KRAS-expressing cells. BI2536-resistant cells remain sensitive to Poloppin, suggesting the potential utility of PBD-inhibitory compounds in overcoming drug resistance to ATP-competitive PLK1 inhibitors.

Notably, Poloppin and Poloppin-II sensitize mutant KRAS-expressing cancer cells to inhibition of the tyrosine kinase receptor c-MET. Since c-MET inhibitors are already in clinical development for the treatment of cancers of the lung, pancreas, or other organs in which KRAS mutations are also frequent, our findings suggest further interesting opportunities for combination therapy.

SIGNIFICANCE

Mutations activating the KRAS oncogene drive human cancers affecting the pancreas, lung, colon, and other tissues. We report here that Poloppin and Poloppin-II, compounds that target the protein-protein interactions of human Polo-like kinases, suppress the growth of KRAS-mutant cancer cells as single agents, or in combination with c-MET inhibitors. The cellular phenotypes evoked by Poloppins speak to a mechanism of action that exploits mitotic vulnerabilities triggered by mutant KRAS expression, via the interruption of phosphopeptide substrate recognition by the Polo-box domains of PLK1 or PLK4. Structure-activity relationships defined by the features of Poloppin-II, a potent analog with favorable pharmacokinetics active *in vivo* against KRAS-mutant xenografts, indicate avenues for further development. Poloppin is less susceptible to *in vitro* drug resistance compared with an ATP-competitive PLK1 inhibitor, and retains activity against cells resistant to the ATP-competitive compound, suggesting methods to combat drug resistance. Poloppin and Poloppin-II act synergistically with Crizotinib, a clinically used inhibitor of the c-MET receptor, against mutant KRAS-expressing cancer cells, revealing an approach for combination therapy. Thus, we implicate the protein-protein interactions of PLK1 and PLK4 in the maintenance of mutant KRAS-expressing cancers, and illustrate the use of compounds that block these interactions in single-agent or combination therapies. Our findings endorse the future potential of strategies to target substrate recognition by human protein kinases for drug development.

STAR★METHODS

Detailed methods are provided in the online version of this paper and include the following:

- KEY RESOURCES TABLE
- CONTACT FOR REAGENT AND RESOURCE SHARING
- EXPERIMENTAL MODEL AND SUBJECT DETAILS
  - Cell Lines
  - Primary Cell Cultures
  - In Vivo Animal Studies
- METHOD DETAILS
  - Engineering of PLK1-PBD and PLK2-PBD Fusions
  - Fluorescence Polarisation Assay
  - Isothermal Titration Calorimetry
  - Mitotic Index Assay
  - Cellular Thermal Shift Assay (CeTSA)
  - NanoLuc® Thermal Shift Assay
  - DiscoverX KINOMEscan Assay
  - Immunofluorescence
  - Western Blotting

(F and G) An analysis of synergy between Crizotinib in combination with Poloppin (F) or Poloppin-II (G) in the Panc-1 cell line expressing mutant KRAS G12D. Line graphs show the percentage change in cell viability after treatment with the individual compounds after 72 hr. The BLISS matrix shows an analysis of a checkerboard titration indicating dose ranges for synergy (indigo) or antagonism (red). Bar graphs show cell viability after 72 hr exposure to optimal synergistic doses of Crizotinib with Poloppin (F) or Crizotinib with Poloppin-II (G), compared with single compound treatment.
Results are typical of at least three independent experiments. Data represent the mean of three independent experiments ± SEM unless otherwise stated. See also Figure S5 and Table S1.

- ○ Viability Assays
- ○ PBD Overexpression in $KRAS^{G12D}$ Cells
- ○ Live Cell Imaging
- ○ Pharmacokinetic Study
- ○ Xenograft Study
- ○ Resistance to PLK1 Inhibitors
- ○ RT-qPCR Assay
- ○ siRNA Sensitization
- QUANTIFICATION AND STATISTICAL ANALYSIS

SUPPLEMENTAL INFORMATION

Supplemental Information includes five figures and one table and can be found with this article online at http://dx.doi.org/10.1016/j.chembiol.2017.07.009.

AUTHOR CONTRIBUTIONS

B.H. and M.R.T. screened a library designed by D.J.H. to identify Poloppin. A.J.N. assisted in screening and characterized Poloppin in biochemical assays. D.W.W., R.B., and R.G.B. conceived and supervised synthetic chemistry for Poloppin-II development. A.J.N. and S.B. characterized the cellular effects of Poloppin and Poloppin-II with A.E., A.C., and E.G.A. S.P.B. and G.J.M. performed NanoLuc thermal shift assays. G.J.M. and C.J.T. supervised xenograft experiments. A.C. and A.E. performed resistance assays. D.P. provided HeLa KRAS G12D cells and assisted in time-lapse imaging. R.A. assisted in organoid derivation. F.E.H. and I.A.P. performed phosphoproteomic analyses. A.J.N., S.B., A.E., B.H., A.C., G.J.M., and A.R.V. collated data, prepared figures, and wrote the manuscript. A.R.V. conceived the project, and supervised it with G.J.M.

ACKNOWLEDGMENTS

All authors, except R.A., D.W.W., R.B., R.G.B., F.E.H., I.A.P., S.P.B., and C.J.T., were supported by Medical Research Council program grants MC_UU_12022/1 and MC_UU_12022/8 to A.R.V. North West Cancer Research supported I.A.P. and F.E.H. Innovate UK funded the development of Poloppin-II by PhoreMost Ltd. (Biomedical Catalyst grant no. 102140). R.A. was supported by MRC research grant MR/K018329/1 and the Rosetrees Trust. We thank members of A.R.V.'s laboratory for helpful comments on the manuscript, Dean Hammond for informatics support, and Meritxell Huch for advice concerning organoid cultures. Work in A.R.V.'s laboratory is funded by the Medical Research Council. David Walker, Richard Boyce and Robert G. Boyle are employees and shareholders of Sentinel Oncology Ltd. Samuel P. Barker, Grahame McKenzie and Chris Torrance are employees and shareholders of PhoreMost Ltd. Ashok R. Venkitaraman is a founder, shareholder and scientific advisor of Sentinel Oncology Ltd. and Phoremost Ltd.

Received: June 28, 2016
Revised: January 16, 2017
Accepted: July 7, 2017
Published: August 10, 2017

REFERENCES

Archambault, V., and Glover, D.M. (2009). Polo-like kinases: conservation and divergence in their functions and regulation. Nat. Rev. Mol. Cell Biol. *10*, 265–275.

Archambault, V., and Normandin, K. (2017). Several inhibitors of the Plk1 Polo-box domain turn out to be non-specific protein alkylators. Cell Cycle *16*, 1220–1224.

Boj, S.F., Hwang, C.I., Baker, L.A., Chio, I.I., Engle, D.D., Corbo, V., Jager, M., Ponz-Sarvise, M., Tiriac, H., Spector, M.S., et al. (2015). Organoid models of human and mouse ductal pancreatic cancer. Cell *160*, 324–338.

Bruinsma, W., Raaijmakers, J.A., and Medema, R.H. (2012). Switching Polo-like kinase-1 on and off in time and space. Trends Biochem. Sci. *37*, 534–542.

Cecchi, F., Rabe, D.C., and Bottaro, D.P. (2012). Targeting the HGF/Met signaling pathway in cancer therapy. Expert Opin. Ther. Targets *16*, 553–572.

Chandra, A., Grecco, H.E., Pisupati, V., Perera, D., Cassidy, L., Skoulidis, F., Ismail, S.A., Hedberg, C., Hanzal-Bayer, M., Venkitaraman, A.R., et al. (2012). The GDI-like solubilizing factor PDEdelta sustains the spatial organization and signalling of Ras family proteins. Nat. Cell Biol. *14*, 148–158.

Di Veroli, G.Y., Fornari, C., Wang, D., Mollard, S., Bramhall, J.L., Richards, F.M., and Jodrell, D.I. (2016). Combenefit: an interactive platform for the analysis and visualization of drug combinations. Bioinformatics *32*, 2866–2868.

Elia, A.E., Cantley, L.C., and Yaffe, M.B. (2003a). Proteomic screen finds pSer/pThr-binding domain localizing Plk1 to mitotic substrates. Science *299*, 1228–1231.

Elia, A.E., Rellos, P., Haire, L.F., Chao, J.W., Ivins, F.J., Hoepker, K., Mohammad, D., Cantley, L.C., Smerdon, S.J., and Yaffe, M.B. (2003b). The molecular basis for phosphodependent substrate targeting and regulation of Plks by the Polo-box domain. Cell *115*, 83–95.

Fabian, M.A., Biggs, W.H., 3rd, Treiber, D.K., Atteridge, C.E., Azimioara, M.D., Benedetti, M.G., Carter, T.A., Ciceri, P., Edeen, P.T., Floyd, M., et al. (2005). A small molecule-kinase interaction map for clinical kinase inhibitors. Nat. Biotechnol. *23*, 329–336.

Garcia-Alvarez, B., Ibanez, S., and Montoya, G. (2006). Crystallization and preliminary X-ray diffraction studies on the human Plk1 Polo-box domain in complex with an unphosphorylated and a phosphorylated target peptide from Cdc25C. Acta Crystallogr. Sect. F Struct. Biol. Cryst. Commun. *62*, 372–375.

Gilmartin, A.G., Bleam, M.R., Richter, M.C., Erskine, S.G., Kruger, R.G., Madden, L., Hassler, D.F., Smith, G.K., Gontarek, R.R., Courtney, M.P., et al. (2009). Distinct concentration-dependent effects of the polo-like kinase 1-specific inhibitor GSK461364A, including differential effect on apoptosis. Cancer Res. *69*, 6969–6977.

Gjertsen, B.T., and Schoffski, P. (2015). Discovery and development of the Polo-like kinase inhibitor volasertib in cancer therapy. Leukemia *29*, 11–19.

Hall, M.P., Unch, J., Binkowski, B.F., Valley, M.P., Butler, B.L., Wood, M.G., Otto, P., Zimmerman, K., Vidugiris, G., Machleidt, T., et al. (2012). Engineered luciferase reporter from a deep sea shrimp utilizing a novel imidazopyrazinone substrate. ACS Chem. Biol. *7*, 1848–1857.

Hammond, D.E., Mageean, C.J., Rusilowicz, E.V., Wickenden, J.A., Clague, M.J., and Prior, I.A. (2015). Differential reprogramming of isogenic colorectal cancer cells by distinct activating KRAS mutations. J. Proteome Res. *14*, 1535–1546.

Hanisch, A., Wehner, A., Nigg, E.A., and Sillje, H.H. (2006). Different Plk1 functions show distinct dependencies on Polo-Box domain-mediated targeting. Mol. Biol. Cell *17*, 448–459.

Hingorani, S.R., Wang, L., Multani, A.S., Combs, C., Deramaudt, T.B., Hruban, R.H., Rustgi, A.K., Chang, S., and Tuveson, D.A. (2005). Trp53R172H and KrasG12D cooperate to promote chromosomal instability and widely metastatic pancreatic ductal adenocarcinoma in mice. Cancer Cell *7*, 469–483.

Huggins, D.J., Venkitaraman, A.R., and Spring, D.R. (2011). Rational methods for the selection of diverse screening compounds. ACS Chem. Biol. *6*, 208–217.

Jafari, R., Almqvist, H., Axelsson, H., Ignatushchenko, M., Lundback, T., Nordlund, P., and Martinez Molina, D. (2014). The cellular thermal shift assay for evaluating drug target interactions in cells. Nat. Protoc. *9*, 2100–2122.

Kang, Y.H., Park, J.E., Yu, L.R., Soung, N.K., Yun, S.M., Bang, J.K., Seong, Y.S., Yu, H., Garfield, S., Veenstra, T.D., et al. (2006). Self-regulated Plk1 recruitment to kinetochores by the Plk1-PBIP1 interaction is critical for proper chromosome segregation. Mol. Cell *24*, 409–422.

Karaman, M.W., Herrgard, S., Treiber, D.K., Gallant, P., Atteridge, C.E., Campbell, B.T., Chan, K.W., Ciceri, P., Davis, M.I., Edeen, P.T., et al. (2008). A quantitative analysis of kinase inhibitor selectivity. Nat. Biotechnol. *26*, 127–132.

Lenart, P., Petronczki, M., Steegmaier, M., Di Fiore, B., Lipp, J.J., Hoffmann, M., Rettig, W.J., Kraut, N., and Peters, J.M. (2007). The small-molecule

inhibitor BI 2536 reveals novel insights into mitotic roles of polo-like kinase 1. Curr. Biol. *17*, 304–315.

Lin, Y., Richards, F.M., Krippendorff, B.F., Bramhall, J.L., Harrington, J.A., Bapiro, T.E., Robertson, A., Zheleva, D., and Jodrell, D.I. (2012). Paclitaxel and CYC3, an aurora kinase A inhibitor, synergise in pancreatic cancer cells but not bone marrow precursor cells. Br. J. Cancer *107*, 1692–1701.

Liu, X. (2015). Targeting polo-like kinases: a promising therapeutic approach for cancer treatment. Transl Oncol. *8*, 185–195.

Luo, J., Emanuele, M.J., Li, D., Creighton, C.J., Schlabach, M.R., Westbrook, T.F., Wong, K.K., and Elledge, S.J. (2009). A genome-wide RNAi screen identifies multiple synthetic lethal interactions with the Ras oncogene. Cell *137*, 835–848.

Mahen, R., Jeyasekharan, A.D., Barry, N.P., and Venkitaraman, A.R. (2011). Continuous polo-like kinase 1 activity regulates diffusion to maintain centrosome self-organization during mitosis. Proc. Natl. Acad. Sci. USA *108*, 9310–9315.

Martinez Molina, D., Jafari, R., Ignatushchenko, M., Seki, T., Larsson, E.A., Dan, C., Sreekumar, L., Cao, Y., and Nordlund, P. (2013). Monitoring drug target engagement in cells and tissues using the cellular thermal shift assay. Science *341*, 84–87.

Martinez Molina, D., and Nordlund, P. (2016). The cellular thermal shift assay: a novel biophysical assay for in situ drug target engagement and mechanistic biomarker studies. Annu. Rev. Pharmacol. Toxicol. *56*, 141–161.

Masser, A.E., Kandasamy, G., Kaimal, J.M., and Andreasson, C. (2016). Luciferase NanoLuc as a reporter for gene expression and protein levels in *Saccharomyces cerevisiae*. Yeast *33*, 191–200.

McCormick, F. (2015). The potential of targeting Ras proteins in lung cancer. Expert Opin. Ther. Targets *19*, 451–454.

Oppermann, F.S., Grundner-Culemann, K., Kumar, C., Gruss, O.J., Jallepalli, P.V., and Daub, H. (2012). Combination of chemical genetics and phosphoproteomics for kinase signaling analysis enables confident identification of cellular downstream targets. Mol. Cell Proteomics *11*, O111.012351.

Ostrem, J.M., Peters, U., Sos, M.L., Wells, J.A., and Shokat, K.M. (2013). K-Ras(G12C) inhibitors allosterically control GTP affinity and effector interactions. Nature *503*, 548–551.

Perera, D., and Venkitaraman, A.R. (2016). Oncogenic KRAS triggers MAPK-dependent errors in mitosis and MYC-dependent sensitivity to anti-mitotic agents. Sci. Rep. *6*, 29741.

Qian, F., Engst, S., Yamaguchi, K., Yu, P., Won, K.A., Mock, L., Lou, T., Tan, J., Li, C., Tam, D., et al. (2009). Inhibition of tumor cell growth, invasion, and metastasis by EXEL-2880 (XL880, GSK1363089), a novel inhibitor of HGF and VEGF receptor tyrosine kinases. Cancer Res. *69*, 8009–8016.

Reindl, W., Yuan, J., Kramer, A., Strebhardt, K., and Berg, T. (2008). Inhibition of polo-like kinase 1 by blocking polo-box domain-dependent protein-protein interactions. Chem. Biol. *15*, 459–466.

Rodig, S.J., and Shapiro, G.I. (2010). Crizotinib, a small-molecule dual inhibitor of the c-Met and ALK receptor tyrosine kinases. Curr. Opin. Investig. Drugs *11*, 1477–1490.

Scharow, A., Raab, M., Saxena, K., Sreeramulu, S., Kudlinzki, D., Gande, S., Dotsch, C., Kurunci-Csacsko, E., Klaeger, S., Kuster, B., et al. (2015). Optimized Plk1 PBD inhibitors based on poloxin induce mitotic arrest and apoptosis in tumor cells. ACS Chem. Biol. *10*, 2570–2579.

Scott, D.E., Coyne, A.G., Venkitaraman, A., Blundell, T.L., Abell, C., and Hyvonen, M. (2015). Small-molecule inhibitors that target protein-protein interactions in the RAD51 family of recombinases. ChemMedChem *10*, 296–303.

Tighe, A., Johnson, V.L., and Taylor, S.S. (2004). Truncating APC mutations have dominant effects on proliferation, spindle checkpoint control, survival and chromosome stability. J. Cell Sci. *117*, 6339–6353.

van den Biggelaar, M., Hernandez-Fernaud, J.R., van den Eshof, B.L., Neilson, L.J., Meijer, A.B., Mertens, K., and Zanivan, S. (2014). Quantitative phosphoproteomics unveils temporal dynamics of thrombin signaling in human endothelial cells. Blood *123*, e22–36.

Van den Bossche, J., Lardon, F., Deschoolmeester, V., De Pauw, I., Vermorken, J.B., Specenier, P., Pauwels, P., Peeters, M., and Wouters, A. (2016). Spotlight on Volasertib: preclinical and clinical evaluation of a promising Plk1 inhibitor. Med. Res. Rev. *36*, 749–786.

van de Weerdt, B.C., Littler, D.R., Klompmaker, R., Huseinovic, A., Fish, A., Perrakis, A., and Medema, R.H. (2008). Polo-box domains confer target specificity to the Polo-like kinase family. Biochim. Biophys. Acta *1783*, 1015–1022.

Vichai, V., and Kirtikara, K. (2006). Sulforhodamine B colorimetric assay for cytotoxicity screening. Nat. Protoc. *1*, 1112–1116.

Wacker, S.A., Houghtaling, B.R., Elemento, O., and Kapoor, T.M. (2012). Using transcriptome sequencing to identify mechanisms of drug action and resistance. Nat. Chem. Biol. *8*, 235–237.

Watanabe, N., Sekine, T., Takagi, M., Iwasaki, J., Imamoto, N., Kawasaki, H., and Osada, H. (2009). Deficiency in chromosome congression by the inhibition of Plk1 polo box domain-dependent recognition. J. Biol. Chem. *284*, 2344–2353.

Xia, X., Maliski, E.G., Gallant, P., and Rogers, D. (2004). Classification of kinase inhibitors using a Bayesian model. J. Med. Chem. *47*, 4463–4470.

Yap, T.A., Olmos, D., Brunetto, A.T., Tunariu, N., Barriuso, J., Riisnaes, R., Pope, L., Clark, J., Futreal, A., Germuska, M., et al. (2011). Phase I trial of a selective c-MET inhibitor ARQ 197 incorporating proof of mechanism pharmacodynamic studies. J. Clin. Oncol. *29*, 1271–1279.

Yuan, J., Sanhaji, M., Kramer, A., Reindl, W., Hofmann, M., Kreis, N.N., Zimmer, B., Berg, T., and Strebhardt, K. (2011). Polo-box domain inhibitor poloxin activates the spindle assembly checkpoint and inhibits tumor growth in vivo. Am. J. Pathol. *179*, 2091–2099.

Zhu, K., Shan, Z., Zhang, L., and Wen, W. (2016). Phospho-pon binding-mediated fine-tuning of Plk1 activity. Structure *24*, 1110–1119.

Zimmermann, G., Papke, B., Ismail, S., Vartak, N., Chandra, A., Hoffmann, M., Hahn, S.A., Triola, G., Wittinghofer, A., Bastiaens, P.I., et al. (2013). Small molecule inhibition of the KRAS-PDEdelta interaction impairs oncogenic KRAS signalling. Nature *497*, 638–642.

STAR★METHODS

KEY RESOURCES TABLE

REAGENT or RESOURCE	SOURCE	IDENTIFIER
Antibodies		
anti- PLK1	Invitrogen	33-1700
anti-phospho-histone H3 (Ser10)	Abcam	ab5176
Hoechst 33342	Invitrogen	H3570
anti-GAPDH	Santa Cruz	sc137179
anti-alpha tubulin	Santa Cruz	sc32293
anti-CREST	Europa	90C-CS1058
anti-c-MET	Cell Signalling Technology	8198
Chemicals, Peptides, and Recombinant Proteins		
Poloppin	this study	
Poloppin II	this study	
PLK1-PBD$_{345-603}$	this study	
PLK2-PBD$_{356-681}$	this study	
TAMRA-MAGPMQTS(pThreonine)PKNGAKK	Designer Bioscience	
BI 2536	SelleckChem	S1109
Nocodazole	Sigma	M1404
Superscript III	Life Technologies	18080044
Rnase H digest	New England Biolabs	M0297S
Amplitaq Gold Polymerase	Applied Biosystems	N8080241
Acetic acid	VWR	20104.334
Sulforhodamine B sodium salt	Sigma	S1402-5G
Trizma base	Sigma	T6066
Trichloroacetic acid solution	Sigma	T0699-100ml
Critical Commercial Assays		
Rneasy Mini Kit	Qiagen	74104
Nextera XT Kit	Cambridge Genomic Services	
CellTitre Glo 3D assay	Promega	
Experimental Models: Cell Lines		
HeLa Parental (Flp-In T-Rex)	Tighe et al., 2004	
HeLa KRAS G12D	Perera and Venkitaraman, 2016	
HeLa PLK1-PBD$_{326-603}$	this study	
HeLa	ATCC	
U2OS	ATCC	
Panc-1	ATCC	
HPAF II	ATCC	
DLD-1	ATCC	
SW1116	ATCC	
HT29	ATCC	
Panc 02.03	ATCC	
Panc 05.04	ATCC	
SW48 Parental (KRAS WT)	Horizon Discovery	
SW48 KRAS G12D	Horizon Discovery	
Experimental Models: Organisms/Strains		
Mouse: LSL-Trp53$^{R172H/+}$	Hingorani et al., 2005; Boj et al., 2015	
Mouse: LSL-Kras$^{G12D/+}$	Hingorani et al., 2005; Boj et al., 2015	

(*Continued on next page*)

Continued

REAGENT or RESOURCE	SOURCE	IDENTIFIER
Mouse: Pdx-1-Cre	Hingorani et al., 2005; Boj et al., 2015	
Athymic nude mice, male	Harlan (UK)	
Oligonucleotides		
Primer: PLK1F 5'-TTGTAACGTTCCCAGCGC		
Primer: PLK1R 5'-GGCACACTGCAGACATGGC		
Primer: PLK2F 5'-TGCTAGTCGGCACCAGAGG		
Primer: PLK2R 5'-TTCGTACCACCACATGTCCA		
Primer: PLK3F 5'-CGCAGCGTAGCAAATCCAG		
Primer: PLK3R 5'-AAAGCTGGTCCCTGATTCCC		
HS_Met_SG QuantiTect Primer Assay	Qiagen	QT00023408
HS_PLK1_SG QuantiTect Primer Assay	Qiagen	QT00049749
HS_PLK2_SG QuantiTect Primer Assay	Qiagen	QT00049406
HS_PLK3_SG QuantiTect Primer Assay	Qiagen	QT00025207
HS_PLK4_SG QuantiTect Primer Assay	Qiagen	QT00029967
HS_Actin_SG QuantiTect Primer Assay	Qiagen	QT01680476
siRNA: c-Met-AACACCCATCCAGAATGTCAT	Qiagen	
siRNA: c-Met-AAGCCAATTTATCAGGAGGTG	Qiagen	
siRNA: c-Met-AAGTATCAGCTTCCCAACTTC	Qiagen	
siRNA: Plk1-CAACGGCAGCGTGCAGATCAA	Qiagen	
siRNA: Plk2-SMARTpool	Dharmacon	
siRNA: Plk3-SMARTpool	Dharmacon	
siRNA: Plk4-AAGGACTTGGTCTTACAACTA	Qiagen	

CONTACT FOR REAGENT AND RESOURCE SHARING

Further information and requests for resources and reagents should be directed to the Lead Contact, Ashok R. Venkitaraman (arv22@mrc-cu.cam.ac.uk).

EXPERIMENTAL MODEL AND SUBJECT DETAILS

Cell Lines

Doxycycline (DOX)-inducible HeLa KRAS G12D and PLK1-PBD$_{326-603}$ cells were generated using the Flp-In T-REx system (Life Technologies) (Tighe et al., 2004). The human KrasG12D or PLK1-PBD$_{326-603}$ transgene was subcloned into a modified pcDNA5/FRT/TO vector containing a single amino-terminal Myc tag, followed by co-transfection with a plasmid encoding Flp recombinase (pOG44) into HeLa FRT/TO cells (a kind gift from Stephen Taylor, University of Manchester). Stable integrants were then selected with 200 ug/ml hygromycin (Roche) and 4 ug/ml blasticidin (Invivogen). Transgene expression was induced by treatment with 100 ng/ml doxycycline (Sigma). HeLa, U2OS, Panc-1, HPAF II, DLD-1, SW1116, HT29, Panc 02.03 and Panc 05.04, cells were obtained from the American Type Culture Collection (ATCC). HeLa, U2OS and Panc-1 were cultured in DMEM containing 10% (v/v) fetal bovine serum (FBS). Panc 02.03 and Panc 05.04 were cultured in RPMI medium containing 15% FBS and 10 units/ml Insulin. DLD-1, SW1116, HT29 were cultured in RPMI medium containing 10% FBS. The SW48 isogenic cell lines (KRAS wild-type and KRAS^{G12D}) were purchased from Horizon Discovery and cultured in RPMI medium containing 10% FBS. All cells were maintained at 37°C with 5% CO_2.

Primary Cell Cultures

KrasWT mouse embryonic fibroblasts (MEFs) were derived from conditional *LSL-Trp53*$^{R172H/+}$ embryos. *LSL-Trp53*$^{R172H/+}$, *LSL-Kras*$^{G12D/+}$ (Hingorani et al., 2005) strains were interbred to obtain *LSL-Kras*$^{G12D/+}$, *LSL-Trp53*$^{R172H/+}$ Kras^{G12D} embryos for MEF preparation. Briefly, MEFs were derived from E13.5 embryos after decapitation and evisceration. Finely minced tissue was passed through a 16-G needle two or three times before dissociated cells were cultured in DMEM with 10% FBS. Cre-mediated recombination to activate the Kras^{G12D} allele was induced via addition of 500nM 4-OH Tamoxifen to the culture media overnight.

Organoid cultures were prepared from the pancreas of *LSL-Trp53*$^{R172H/+}$, *Pdx-1-Cre* (for Kras WT organoids) or *LSL-Trp53*$^{R172H/+}$, *LSL-Kras*$^{G12D/+}$, *Pdx-1-Cre* (for Kras^{G12D} organoids KPC3760 and KPC26684) strains (Hingorani et al., 2005). Pancreatic ducts were manually picked after tissue mincing and overnight enzymatic digestion with 0.012% (w/v) collagenase XI (Sigma) and 0.012% (w/v) dispase (GIBCO) in DMEM media containing 1% FBS (GIBCO), before seeding in growth-factor-reduced (GFR) Matrigel (BD) (Boj et al., 2015).

In Vivo Animal Studies

Male athymic mice were purchased from Harlan (UK) and acclimatised for 7 days prior to study commencement. Animals were housed in IVC cages (5 per cage) with individual mice identified by tail marking. All animals were allowed free access to a standard certified commercial diet and sanitised water during the study. The holding room was maintained under standard conditions: 20-24°C, 40-70% humidity and a 12h light/dark cycle. All experiments were performed under approved ethical and procedural guidelines.

METHOD DETAILS

Engineering of PLK1-PBD and PLK2-PBD Fusions

A region of the human PLK1 cDNA sequence (residues 345–603) or human PLK2 cDNA sequence (residues 356-681) was amplified by PCR and cloned into a pGEX-6P1 vector (Invitrogen, Carlsbad, CA) to generate recombinant protein in C41 strain *E. coli* (Garcia-Alvarez et al., 2006). Bacteria were disrupted using an Emulsiflex c5 homogenizer (Avestin) and lysates were passed onto a glutathione S-transferase (GST)-Sepharose column in the presence of 50mM Hepes pH 7.5, 200mM NaCl, 1 mM EDTA, 1mM EGTA and 1mM DTT. Bound fractions were cleaved on column at 4°C overnight with PreScission protease (GE healthcare). Purified fractions were polished by gel chromatography using a Sephadex G25 (GE healthcare) column, collected and concentrated. Fractions were tested by Western blotting using PLK1 antibody (Invitrogen 33-1700) to validate protein purification.

Fluorescence Polarisation Assay

Final concentrations of assay components used in binding assays were as follows: TAMRA-labelled consensus PLK1 PBD binding phosphopeptide, 5-TAMRA-MAGPMQS(pThreonine)PLMGAKK-acid 10nM; PLK1 PBD (aa345-603) 84nM (2.5ng/μl). Assays were carried out in PBS (pH 7.4) plus 0.03% tween. DMSO controls were run alongside all experimental compounds and % inhibition normalised to these controls. Compounds were titrated 2 fold from a top concentration of 250μM giving a maximum final concentration of DMSO in the assay of 0.25%; however the assay can tolerate DMSO up to 5%. Compound and PBD protein were incubated together at 22°C for 1 hour prior to addition of the TAMRA labelled binding phosphopeptide. The total assay volume per well was 45μl. All experiments were performed in NBS black 384-well microtiter plates (Corning). Fluorescence Polarisation (FP) was read using a BMG PheraStar plate reader with a 540/590/590nm FP module and unbound 10nm TAMRA-labelled peptide set to a FP value of 35mP.

For FP assays using PLK2 PBD (aa356-681), the same procedure was applied except that TAMRA-MAGPMQTS(pThreonine) PKNGAKK was used as the consensus binding phosphopeptide.

For PLK1 PBD FP high-throughput screen the assay components were as above, compounds were screened at an approximate final concentration of 125μM and final DMSO concentration of 1%. Appropriate volumes of TAMRA-labelled phosphopeptide and Plk1 PBD at 3X final concentration were made up in PBS + 0.1% tween and PBS + 1mM DTT respectively and placed in reservoirs on the Beckman Coulter Biomek FX^p Automated Workstation deck in the positions indicated by the scheduling software. Compound dilution plates, black 384 well NBS assay plates and tip racks were loaded into the Cytomat as indicated by the scheduling software. The appropriate Fluorescence Polarisation program was initiated on the BMG PheraStar plate reader and was manually set using an assay plate and 10nm TAMRA-labelled peptide to a FP value of 35mP. All labware was checked to ensure that it is correctly positioned before the scheduling software was enabled and the HTS run was initialised. Test compounds were diluted to 3X final concentration in the dilution plates. 15μl of diluted compound was added into each well of the assay plate followed by 15μl of PBD protein and 15μl of TAMRA-phosphopeptide. Plates were incubated at room temperature for between 20 and 40 min on the robot's deck or in the Cytomat before the Fluorescence Polarisation was read using the BMG PheraStar plate reader. Test compounds were assayed in triplicate. Mean % inhibition normalised to DMSO control (1%) was calculated for each compound.

Isothermal Titration Calorimetry

In all titrations, PBDaa345-603 protein was used at 25 μM and buffered in HEPES (50 mM, pH 7.4), NaCl (200 mM), and DTT (1mM). Protein was diluted into buffer with DMSO at 5% (v/v). Poloppin was prepared by diluting from DMSO stock into the same buffer containing DMSO at 5% (v/v). Great care was taken to match the concentration of DMSO in the ligand and protein samples as closely as possible. In a typical experiment, protein (25 μM) was loaded in the sample cell, and a total of 20 injections of 8μL were made at 2 min intervals from a 200 μL syringe rotating at 1000 rpm and loaded with ligand solution (0.5 mM). In all titrations, an initial injection of 2 μL ligand was made, and these data were discarded during data analysis. The thermodynamic parameters were obtained by fitting the data to a single-site binding model with a stoichiometry of 1. ITC experiments were performed at 20 °C with a MicroCal ITC200 instrument, and all data were analysed with the software implemented in Origin (version 7) (Scott et al., 2015).

Mitotic Index Assay

Cells were plated at 10 000/well in 96-well plates and incubated overnight. The following day compound stocks in DMSO were diluted in medium then added to cells with a maximum final DMSO concentration on cells of 0.2%. Cells were incubated with compound for 24 h then fixed in 3.7% formaldeyde. Cells were permeabilised with 0.1% Triton X-100 then incubated with anti-phospho-histone H3 (Ser10) antibody (Abcam ab5176). The cells were washed with PBS then incubated with AlexaFluor 488 labelled goat anti-rabbit IgG (Invitrogen A11034) in the presence of 4ug/ml Hoechst 33342 (Invitrogen H3570). Cells were washed in PBS then imaged on an

Arrayscan VTi HCS instrument (Thermo Fisher) using the Target Activation V4 Bioapplication. A user-defined threshold was applied to identify mitotic cells based on the intensity of phospho-histone H3 staining.

Cellular Thermal Shift Assay (CeTSA)

Two million HeLa cells were assessed for viability using Trypan blue and aliquoted into PCR tubes for lysate preparation and CeTSA. Aggregation temperature (Tagg) was determined for PLK1 PBD with a Biorad Tetrad gradient maker, using a final concentration of 1mM or 0.5mM for Poloppin or Poloppin-II, respectively, in 5% DMSO (Jafari et al., 2014). Samples were assessed using PLK1 antibodies (Invitrogen 33-1700) and GAPDH (Santa Cruz sc137179) and Li-Cor antibody (926-32252). Quantification was done with Odyssey Li-Cor technology.

NanoLuc® Thermal Shift Assay

HeLa cells were transfected with plasmids encoding NanoLuc fused to the N-termini of full-length human PLK1, PLK2, PLK3, PLK4 or p38α MAPK. Twenty-four h after transfection, the thermal stability of the fusion proteins was tested using a modified version of the assay protocol supplied by Promega. Briefly, cells were harvested by tyrpsinisation and centrifugation, counted, and resuspended to 200,000 cells/ml in 3mls of serum-free OptiMEM media. Cells were then permeabilised using with 50ug/ml digitonin in DMSO for 5 min and incubated with test compounds at 100uM, plus protease inhibitor cocktail (Promega G6521), in a final concentration of 5% DMSO for 1 h at 37°C. After incubation, 100 μl/well of cells were aliquoted into a 96-well, thin-walled PCR plate and then heated for 3 minutes using a pre-warmed MJ Tetrad DNA Engine set to run a temperature gradient of 42 to 66°C. The plates were then cooled to room temperature for 10 min, and then 80μl of each sample was transferred to a white-walled 96-well Nunc plate before NanoBRET™ NanoGlo Substrate diluted in Opti-MEM was added. NanoLuc enzyme activity was read out within 10 min using a BMG Clariostar plate reader.

DiscoverX KINOMEscan Assay

A panel of 55 proteins (comprising 51 human kinases structurally or functionally related to the PLKs, plus the kinase catalytic domains of PLK1, PLK2, PLK3 and PLK4) were selected for testing by DiscoverX using the KINOMEscan assay platform. Compounds were supplied as 40x DMSO stocks and were tested at a final concentration of 50μM for Poloppin and 5μM for Poloppin-II. Binding interactions were reported as percentage of control where lower numbers indicate stronger binding. A cut-off of <35% was applied to report significant binding.

Immunofluorescence

Cells were plated on glass coverslips and incubated overnight. Cells were treated with compound diluted in medium for 24hrs. Medium was then removed and cells fixed in 20mM PIPES pH8, 20mM HEPES pH8, 5mM EGTA, 2mM MgCl, 0.2% Triton X-100 and 3.7% formaldehyde. Cells were washed in PBS containing 0.1% Tween-20 then incubated with anti-alpha tubulin antibody (Santa Cruz sc-32293) and anti-CREST antibody (Europa 90C-CS1058). Cells were washed then incubated with Alexa Fluor 568 labelled goat anti-mouse IgG (Invitrogen A11031) or Alexa Fluor 633 labelled goat anti-mouse IgG (Invitrogen A21052) and Alexa Fluor 488 goat anti-human IgG (Invitrogen A11013). Cells were washed and then mounted on glass slides in Vectashield mounting medium containing DAPI.

Western Blotting

Total protein was isolated by directly lysing the cells in non-denaturing lysis buffer (20 mM Tris HCl pH 8, 137 mM NaCl, 10% glycerol, 1% Nonidet P-40 (NP-40), 2 mM EDTA). Protein lysates were resolved on SDS-PAGE, transferred onto a Immobilon-P, PVDF membrane (Millipore), and probed with: Plk1 antibody (Invitrogen 33-1700), c-MET antibody (Cell Signalling Technology 8198). Secondary HRP-conjugated antibodies were used and the signal was detected using an Amersham enhanced chemiluminescence system (ECL, GE Healthcare).

Viability Assays

SW48 Parental, SW48 G12D, Panc-1, HPAF-II, Panc 02.03 and Panc 05.04 cell lines were seeded overnight at 5000 cells per well in a 96 well plate in recommended media (ATCC). Compounds were added the following day and viability was measured at 72h using the sulforhodamine B (SRB) colorimetric assay as follows. Cells were fixed by replacing media with 1% v/v Trichloroacetic Acid (TCA) (Sigma-Aldrich) at 4°C and incubated for 1h also at 4°C. Wells were washed thoroughly with copious amounts of deionised water before staining with Sulforhodamine B (SRB) (Sigma-Aldrich) solution (0.057% w/v in 1% v/v Acetic Acid (VWR)) for 30 min. Stain was aspirated and wells washed with several changes of 1% v/v Acetic Acid until unbound stain was removed, before air-drying. Bound stain was solubilized with 100μl / well 10mM Tris (pH 8.0) (Trizma Base (121.1) Sigma-Aldrich) with agitation for 10 min. Fluorescence intensity was recorded at Ex540nm / Em590nm using a BMG Pherastar Plus plate reader (Vichai and Kirtikara, 2006).

Pancreatic organoids were extracted and cultured as detailed above (Boj et al., 2015). In brief, pancreatic organoids were seeded at 600 cells per well in a 96 well plate and allowed to grow for 7 days with media replenishment on the 3rd day. Full-grown organoids were then treated with compounds for 72h and viability determined using CellTitre Glo 3D assay (Promega). For viability assays with c-MET siRNA, cells were reverse transfected in a 6 cm dish using Dharmafect 1 (GE Dharmacon) per manufacturer's instructions. On the following day, cells were trypsinised and plated onto 96 well plates or 6 well plates. The 96 well plates were treated with a dose

range of compounds or DMSO, and viability was determined via SRB colorimetric assay. Cells in the 6 well plates were collected at the end of experiment to corroborate knockdown of c-MET by Western blotting.

PBD Overexpression in KRAS^{G12D} Cells

HeLa cells engineered to inducibly overexpress Plk1-PBD (HeLaPBD-326) were transfected with mCherry tagged KRAS G12D (HeLaPBD-326 mCherry KRAS G12D). After selection in Puromycin (0.3ug/ml) for one month, the mCherry positive population was sorted by FACS and alongside the parental cells subjected to a viability assay. HeLaPBD-326 and HeLaPBD-326 mCherry KRAS G12D cells were seeded at 2.5x10^3 cells/well in 96-well plates. The following day cells were treated with a titration of doxycycline. At the end of 72h incubation period SRB colorimetric assay was performed to assess viability.

Live Cell Imaging

Five x10^4 cells were seeded on each well of 4-well coverglass Lab-Tek chambers in a volume of 800 μl. HeLa-G12D cells were induced with Doxycycline (Dox) 24h before seeding and kept in Dox for the rest of the experiment. The day after seeding, DMSO or Poloppin (at the respective GI_{50} for cell killing: 10μM (HeLa) or 5μM (HeLa-KRAS G12D) was added to the wells and live cell imaging was performed using a fully motorised Leica DMI6000 B inverted microscope equipped with Evolve® 512 EMCCD Camera with an exposure time of 98ms. Bright field images were taken with a HCX PL APO 40x/0.85 CORR CS DRY objective every 5 min for duration of 48h.

Pharmacokinetic Study

Mice were dosed orally with 10mg/kg Poloppin-II, and blood samples were taken from 3 mice per time point, and frozen until all samples were available. Plasma was prepared from each sample and Poloppin-II levels analysed by a pre-established methodology (UHPLC-TOF MS using electrospray ionisation).

Xenograft Study

Tumours were established by sub-cutaneous implantation of 2 x 10^6 HCT116 cells suspended in 50% Matrigel into the flanks of a cohort of mice. When a sufficient number of mice had developed tumours of approximately 100mm3, they were randomly assigned to three groups: Vehicle, 50mg/kg Poloppin-II or 200mg/kg Poloppin-II, with 10 mice per group. Mice were treated orally according to their treatment group once every three days. Tumour volume was measured three times per week with calipers. Body weight was measured three times per week. In addition to mice assigned to tumour volume efficacy arm of the study, 16 mice with established HCT116 xenografts were assigned to either Vehicle or 200mg/kg oral dosing groups, with 8 mice per group. 24 hours after a single dose, mice were euthanised, tumours removed, snap frozen and 30-50microgram of lysates were analysed by Western blotting on 4-12% Bis-Tris acrylamide gels, and transferred using the Invitrogen iBlot dry system. Phospho-Histone H3 was detected using Abcam antibody ab5176, beta-actin with Sigma antibody a5316. Secondary detection was achieved with the appropriate HRP-conjugated antibodies from Cell Signalling Technology. HRP was visualised with ECL-Plus (GE Healthcare).

SILAC and mass spectrometry Samples were processed for phosphoproteomic analysis as follows. In order to generate light, medium and heavy stable isotope-labelled HeLa S3 cells, arginine and lysine free DMEM medium (Dundee Cell Products) was supplemented with 10% dialysed FBS (GIBCO), 200 mg/L L-proline and either L-lysine (Lys0) together with L-arginine (Arg0), L-lysine-$^2H_4$ (Lys4) with L-arginine-U-$^{13}C_6$ (Arg6) or L-lysine-U-$^{13}C_6$-$^{15}N_2$ (Lys8) with L-arginine-U-$^{13}C_6$-$^{15}N_4$ (Arg10) at final concentrations of 28 mg/L for the arginine and 146 mg/L for the lysine until fully metabolically labelled. For SILAC experiments, 2 x 15 cm dishes per condition were treated with PLK inhibitors or DMSO and mitotic cells isolated by shake-off. To increase the yield for DMSO treated control cells, media was replaced after initial shake-off and plates returned to the incubator. A second round of shake off was performed after a further 1 hour incubation. Cells were washed in cold PBS, lysed in lysis buffer (100 mM Tris pH 7.6, 2% SDS, 50 mM NaF, 1 x PhosStop [Roche], 1 x mammalian protease inhibitor [Sigma]), quantified and 1.67 mg of each lysate mixed 1:1:1 (Hammond et al., 2015; van den Biggelaar et al., 2014).

Filter-aided sample preparation (FASP) was used to trypsin digest and isolate peptides (Hammond et al., 2015; van den Biggelaar et al., 2014). For proteome analysis, 5% of the material was removed after FASP. Phosphopeptides were fractionated from the remaining 95% using strong cation exchange (Resource S, GE Healthcare), and enriched using TiO_2 (van den Biggelaar et al., 2014). All samples were cleaned up using C18 stage tips before analysis by LC-MS. Samples were analysed by the University of Warwick Proteomics Research Technology Platform. Samples were fractionated by reversed phase chromatography using an Ultimate 3000 RSLCnano system (Dionex) coupled to a Thermo Orbitrap Fusion (Thermo Scientific). Samples were loaded onto an Acclaim PepMap μ-precolumn cartridge 300 μm i.d. x 5 mm, 5 μm, 100 Å for 8 min at 10 μL/min in 2% acetonitrile, 0.1% trifluoroacetic acid. For proteome the peptides were then eluted onto an Acclaim PepMap RSLC 75 μm i.d. x 50 cm, 2 μm, 100 Å (Thermo Scientific) at 250 nL/min,using a 110 minute gradient of 8-25% acetonitrile in 0.1% formic acid followed by a 22 minute gradient from 25-35% acetonitrile in 0.1% formic acid. For phosphopeptides sample was eluted from the precolumn at 300 nL/min onto an Acclaim PepMap RSLC 75 μm x 25 cm, 2 μm, 100 Å (Thermo Scientific) using a 37 min gradient from 8-25% acetonitrile in 0.1% formic acid, then an 8 min gradient from 25-35% acetonitrile in 0.1% formic acid. Eluting peptides were converted to gas-phase ions by means of electrospray ionization and analysed on a Thermo Orbitrap Fusion (Thermo Scientific). Survey scans of peptide precursors from 375 to 1500 m/z were performed at 240K resolution (at 200 m/z) with a 2x10^5 or 4x10^5 ion count target for proteomes or phosphoproteomes, respectively. The maximum injection time was set to 150 ms. Tandem MS was performed by isolation at 1.2 Th using the quadrupole,

HCD fragmentation with normalized collision energy of 30, and rapid scan MS analysis in the ion trap. The MS^2 ion count target was set to $3x10^3$ or 10^4 for proteomes or phosphoproteomes, respectively. The maximum injection time was 200 ms. Precursors with charge state 2–6 were selected and sampled for MS^2. Dynamic exclusion duration was set to 60 s for proteomes or 50 s for phosphoproteomes, with a 10 ppm tolerance around the selected precursor and its isotopes. Monoisotopic precursor selection was turned on. The instrument was run in top speed mode.

Resistance to PLK1 Inhibitors

HCT116 wild-type cells (Horizon Discovery Ltd.) cultured in McCoy's 5A (Life Technologies #26600) medium supplemented with 10% Foetal Calf Serum (Life Technologies #10270) were treated on three occasions with titrations of the PLK1 ATP-competitive inhibitor BI 2536 (Selleckchem #S1109) or Poloppin in order to determine the 2.5x GI_{50}, 3x GI_{50} and 5x GI_{50} values for each compound required to initiate the development of resistance. Cells were subsequently treated with 2.5x GI_{50}, 3x GI_{50} and 5x GI_{50} concentrations of either compound, changing media and compound every three to four days until resistant colonies emerged or no viable cells remained. Single cell clones were isolated from cells resistant to 5x GI_{50} BI 2536. Equivalent experiments using 3x GI_{50} or 2.5x GI_{50} doses of Poloppin or BI2536 yielded identical results (data not shown). BI 2536-resistant clones (HCT116 BI 2536-Res. Clones) were treated with BI2536 or Poloppin, alongside drug-sensitive parental HCT116 cells (HCT116 wt), and viability was assessed after 72 h. Viability is represented as percentage survival normalised to DMSO control. For PLK1 mutation analysis resistant clones were enriched for mitotic cells by treatment for 16h with Nocodazole (Sigma M1404) prior to harvesting by scraping and RNA extraction using Qiagen RNeasy Mini Kits (Qiagen: 74104). cDNA was synthesized using Life Technologies First Strand Synthesis protocols with Superscript III (Life Technologies 18080044), RNase H digested (New England Biolabs M0297S) and subject to PCR for the PLK1 following amplicon. Primers were designed to accommodate additional UTR cDNA in order for Nextera XT kits (Cambridge Genomic Services, University Of Cambridge) to append tagging information without subsequent loss of sequence integrity, as follows: PLK1F 5'-TTGTAACGTTCCCAGCGC, PLK1R 5'-GGCACACTGCAGACATGGC, PLK2F 5'-TGCTAGTCGGCACCAGAGG, PLK2R 5'-TTCGTACCACCACATGTCCA, PLK3F 5'-CGCAGCGTAGCAAATCCAG, and PLK3R 5'-AAAGCTGGTCCCTGATTCCC PCR reactions were carried out using Amplitaq Gold polymerase (Applied Biosystems N8080241) with a 25-cycle programme. PCR products were gel-purified (Qiagen 28704) before being mixed in equimolar quantities in the case of each cell line and submitted for sequencing. Prepared samples were submitted for next generation sequencing using MiSeq Nano run with Nextera XT library preparation. Sequencing and subsequent bioinformatics were performed by Cambridge Genomic Services (University Of Cambridge).

RT-qPCR Assay

Total RNA from cultured cells was extracted using TRIzol reagent following the manufacturer instructions (Qiagen). Total RNA samples were submitted to Cambridge Genomic Services (CGS) for whole-transcript profiling on the Affymetrix Gene ST plate arrays and subsequent bioinformatics analysis. For relative quantification of gene expression levels and microarray validation experiments, equal amounts of cDNA were synthesized using SuperScript® III Reverse Transcriptase kit (Invitrogen). qPCR was performed using QuantiTect SYBR® Green PCR Kit and cycling conditions used were 94°C for 15 sec, 55°C for 30 sec, and 70°C for 30 sec for 40 cycles. The specificity of the reaction was verified by melt curve analysis. Experiments were performed using StepOne Real-Time PCR Systems (Applied Biosystems). Primers used in this assay are: c-Met (QT00023408), PLK1 (QT00049749), PLK2 (QT00049406), PLK3 (QT00025207), PLK4 (QT00029967) and beta-actin (QT01680476) from Qiagen.

siRNA Sensitization

Cells were reverse-transfected with 50nM (SW48 parental, SW48 KRAS G12D and Panc-1) or 25nM (HeLa Flp-In T-REx parental cells) siRNA using Dharmafect (GE Dharmacon) as manufacturer's recommendations. Where transfected cells were subsequently treated with compounds, the cells were allowed to settle overnight and then treated with compound at the indicated concentration the following day. Viability was assessed after 72 hrs, with the SRB colorimetric assay. Knockdown efficiencies were determined by Western blot or RT-qPCR. Oligos against the following target sequences were used. c-MET (Qiagen): AACACCCATCCAGAAT GTCAT, AAGCCAATTTATCAGGAGGTG, AAGTATCAGCTTCCCAACTTC. PLK1 (Qiagen): CAACGGCAGCGTGCAGATCAA. PLK2 (SMARTpool; Dharmacon): ACACAGAAGGAGAACGAUA, CAUCAAUGAGGAUAGGAUA, CUGCCUAGUGUUACUGAUA, ACAAA GUCUACGCCGCAAA. PLK3 (SMARTpool; Dharmacon): GCGAGAAGAUCCUAAAUGA, GCAAGUGGGUUGACUACUC, GCACAU CCGUUGGCCAUCA, GACCUCAAGUUGGGAAAUU. PLK4 (Qiagen): AAGGACTTGGTCTTACAACTA.

QUANTIFICATION AND STATISTICAL ANALYSIS

For live cell imaging analysis image sequences were viewed using ImageJ software and cell fate was analysed manually. Time spent in mitosis for different groups was compared with a Kruskal-Wallis test and Dunn's multiple comparison correction.

For phosphoproteomics analysis raw MS peak lists were searched against the Uniprot database (downloaded July 2016) using the Andromeda search engine and processed with the MaxQuant software suite (version 1.5.5.1).

Nanoluciferase enzyme activity of PLK1- or PLK4-Nanoluc fusion protein was analysed using GraphPad Prism. Data were normalised as follows: (treatment A RLU value at X°C temp/treatment A RLU value at 42°C temp) x 100. Using the Boltzmann sigmoidal equation, a curve was then fitted to the normalised data. From this non-linear fit, a V_{50} value was generated, which in this context

represents the aggregation onset temperature (Tagg), at which 50% of the PLK1- or PLK4-Nanoluc fusion protein has aggregated.

Bliss model independence synergy was performed as follows: 96-well plates were treated with a dilution series of each drug in an 8 × 8 checkerboard pattern of combinations (Lin et al., 2012). After SRB staining to obtain the growth inhibition data, we used Combenefit software to identify synergistic drug combinations, focusing on BLISS independence (Di Veroli et al., 2016). The single-agent inhibition values were used to calculate a drug combination surface under the assumption of an additive effect. Regions of synergy were then detected by comparing obtained data from a combination with the calculated additive effect (derived by subtracting the calculated additive inhibition values from the measured inhibition to obtain the final difference values). In the final synergy surface, positive values hence indicate synergy regions, whereas negative difference values identify antagonistic effects.

Cell Chemical Biology, Volume *24*

Supplemental Information

Modulating Protein-Protein Interactions of the Mitotic Polo-like Kinases to Target Mutant KRAS

Ana J. Narvaez, Suzan Ber, Alex Crooks, Amy Emery, Bryn Hardwick, Estrella Guarino Almeida, David J. Huggins, David Perera, Meredith Roberts-Thomson, Roberta Azzarelli, Fiona E. Hood, Ian A. Prior, David W. Walker, Richard Boyce, Robert G. Boyle, Samuel P. Barker, Christopher J. Torrance, Grahame J. McKenzie, and Ashok R. Venkitaraman

SUPPLEMENTAL INFORMATION

Figure S1. Related to Figure 1. Poloppin is a drug-like inhibitor of phosphopeptide binding by PLK1 PBD. (A) Schematic representation of the fluorescence polarisation (FP) assay for inhibitors of substrate recognition by the PLK1 PBD. (B) Determination of the Z Factor. Fluorescence polarisation values for the TAMRA-labelled PLK1 PBD-binding Consensus phosphopeptide bound to PLK1 PBD ((wells 1–576) mean FP value = 128.5mP (SD = 5.2)) compared to unbound ((wells 577–1152) mean FP value = 32.4mP (SD = 2.0)) are shown. Z Factor was calculated using the equation Z Factor = 1 − (3xSD bound + 3xSD unbound)/(mP bound − mP unbound), where SD is the standard deviation from the mean, and mP is the mean fluorescence polarisation. The Z Factor value in this representative experiment was 0.774. (C) Validation of FP assay using phosphopeptide substrates. The ability of unlabelled peptide sequences representing either the phosphorylated (marked P-) or non-phosphorylated forms of known PLK1 PBD substrates (e.g. CDC25C, PBIP1) to compete for binding of a TAMRA-labelled consensus phosphopeptide (TAMRA-MAGPMQS(**pThr)**PLMGAKK) was compared. Unlabelled forms of the consensus peptide itself were also used as positive (P-Consensus) or negative (Consensus) controls. (D) DiscoverX KINOMEscan assay results for Poloppin (50μM). Binding interactions to a panel of 55 kinases were reported as percentage of control where a hit is identified as <35%. (E) Activity of Poloppin against PLK2 PBD in the FP assay. Poloppin was titrated against the TAMRA-labelled PLK2 PBD consensus phosphopeptide (TAMRA-MAGPMQTS(**pThr**)PKNGAKK) bound to PLK2 PBD. The compound shows approximately 3-fold selectivity for PLK1-PBD over PLK2-PBD. Data represent the mean of three independent experiments ± SEM.

FIGURE S1

A

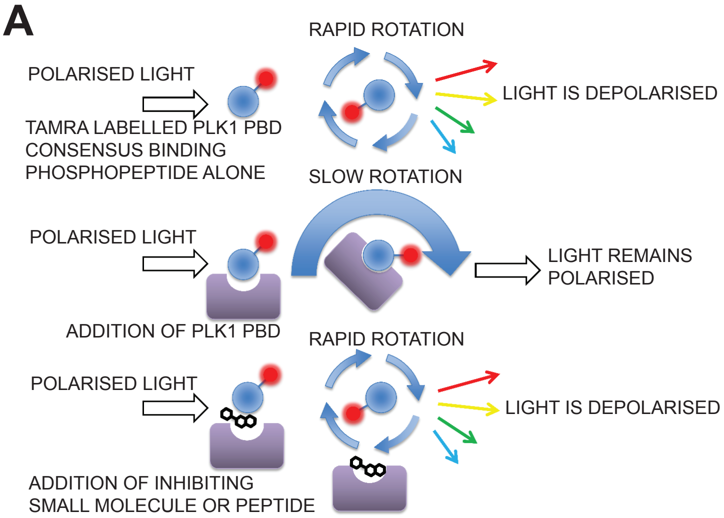

B

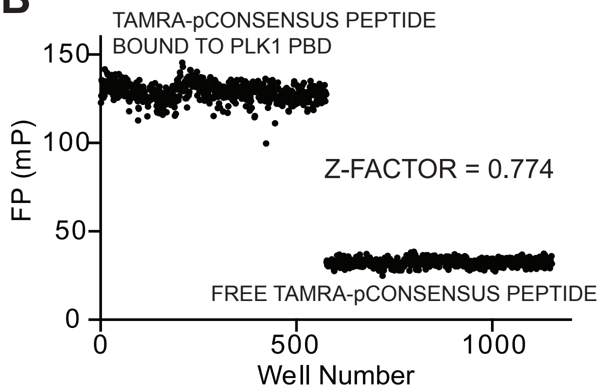

C

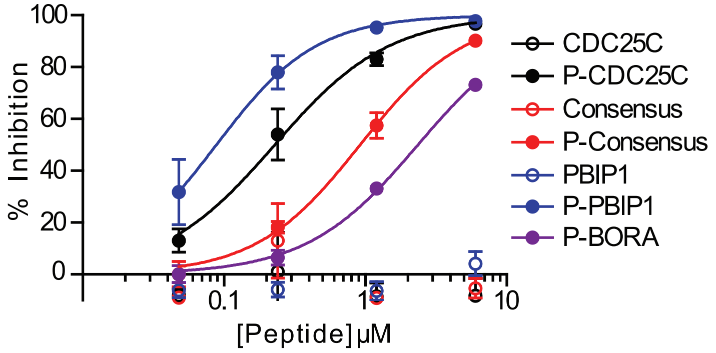

D

Cutoff= 35% Control

Gene Symbol	% Control	Gene Symbol	% Control
AAK1	92	NEK9	95
AURKA	81	PKMYT1	98
AURKB	87	PLK1	100
AURKC	81	PLK2	78
BMP2K	68	PLK3	83
BUB1	99	PLK4	88
CAMKK1	77	EIF2AK2	69
CAMKK2	82	DSTYK	49
CDK2	84	ROCK2	100
CSNK2A1	87	SBK1	61
CSNK2A2	84	SgK110	94
EIF2AK1	97	STK16	81
ERN1	76	STK35	100
GAK	100	STK36	91
EIF2AK4	100	TBK1	75
GSG2	100	TLK1	100
CHUK	100	TLK2	90
IKBKB	100	TTK	85
IKBKE	55	ULK1	84
NEK1	57	ULK2	100
NEK10	98	ULK3	94
NEK11	100	WEE1	99
NEK2	95	WEE2	86
NEK3	72	WNK1	100
NEK4	100	WNK2	100
NEK5	83	WNK3	100
NEK6	96	WNK4	100
NEK7	68		

E

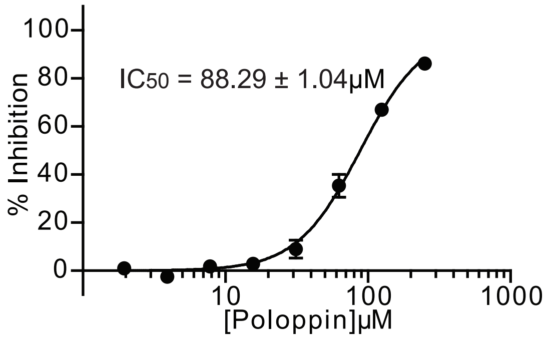

Figure S2. Related to Figure 3. Poloppin exerts its cellular effects via the PBD domains of PLKs.
NanoLuc thermal shift assay for NanoLuc-p38αMAPK fusion protein. Thermal stability curves are shown in the presence of DMSO, the p38α inhibitor AMG548 (at 100nM) and Poloppin or Poloppin-II (at 100μM)

FIGURE S2

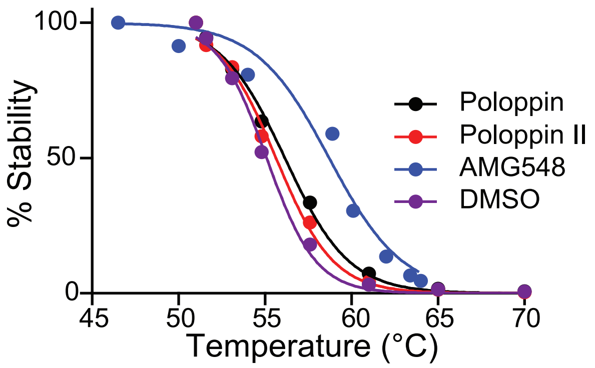

100
50
0
% Stability
45
50
55
60
65
70
Temperature (°C)
Poloppin
Poloppin II
AMG548
DMSO

Figure S3. Related to Figure 5. The optimised analog Poloppin-II is effective by systemic oral administration *in vivo* against mutant KRAS-expressing xenografts. (A) DiscoverX KINOMEscan assay results for Poloppin-II (5μM). Binding interactions to a panel of 55 kinases were reported as percentage of control where a hit is identified as <35%. (B) Quantitation of mitotic phenotypes in cells treated with an EC_{50} concentration of Poloppin-II. Cells were treated as in Figure 3D. (C) Nanoluciferase CeTSA for PLK1 and PLK4. Cells were treated with 100μM Poloppin-II or PB114 over the indicated temperature range. (D) Activity of Poloppin-II against PLK1-PBD and PLK2-PBD in the FP assay. (E) Pharmacokinetic analysis of Poloppin-II, following plasma levels after a single dose of 10mg/kg administered orally. Plasma was sampled at 10 time points (n=3 mice per time point, terminal sampling) over 24 h. Poloppin-II exhibits an oral bioavailability in excess of 90%, a per oral half-life of 15 h, and a volume of distribution of 23 L/kg. (F) Dosing schedule for Poloppin-II. Mice were dosed every three days either with 50mg/kg and 200mg/kg of Poloppin-II, or vehicle as control. Treatment with 200mg/kg Poloppin-II was suspended after seven days. (G) Body weight changes for mice at different doses. Change in body weight during dosing time represented as bodyweight change normalized to pre-treatment levels. (H) Statistical analysis of Poloppin-II treated animals (2 way ANOVA). Percentage of treatment to control (T/C) show shrinkage in tumour volume of 53.3% or 47.7% after 15 days of treatment with 50mg/kg and 200mg/kg respectively. (I) Intra-tumoral effect of Poloppin-II assessed in 8 individual mice by Western blot analysis for phospho-histone H3 phosphorylation on Ser10 in tumor lysates 24 h after treatment at the indicated dose. Where IC_{50}, EC_{50} or GI_{50} values are shown, values are the mean of three independent experiments ± SEM unless otherwise stated.

FIGURE S3

A

Cutoff Value = 35% Control

Gene Symbol	% Control	Gene Symbol	% Control
AAK1	100	NEK9	92
AURKA	83	PKMYT1	100
AURKB	95	PLK1	100
AURKC	85	PLK2	85
BMP2K	85	PLK3	74
BUB1	90	PLK4	90
CAMKK1	91	EIF2AK2	90
CAMKK2	97	DSTYK	75
CDK2	89	ROCK2	100
CSNK2A1	96	SBK1	61
CSNK2A2	92	SgK110	100
EIF2AK1	98	STK16	92
ERN1	74	STK35	71
GAK	100	STK36	91
EIF2AK4	97	TBK1	80
GSG2	77	TLK1	99
CHUK	100	TLK2	94
IKBKB	100	TTK	83
IKBKE	75	ULK1	84
NEK1	100	ULK2	100
NEK10	94	ULK3	88
NEK11	100	WEE1	98
NEK2	94	WEE2	92
NEK3	74	WNK1	97
NEK4	100	WNK2	100
NEK5	99	WNK3	100
NEK6	99	WNK4	91
NEK7	79		

B

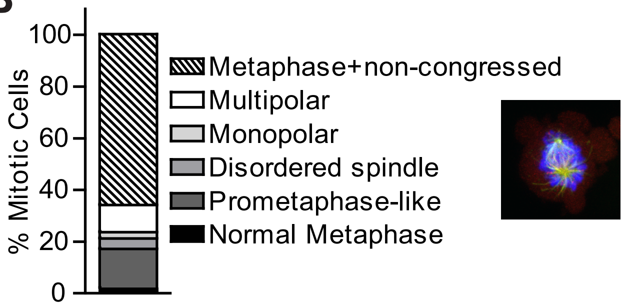

C

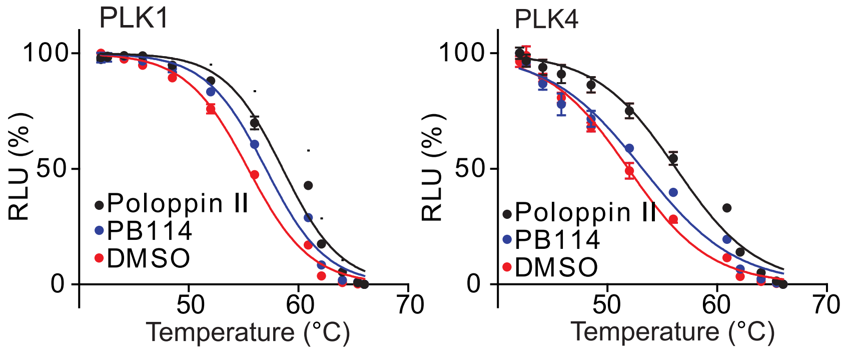

D

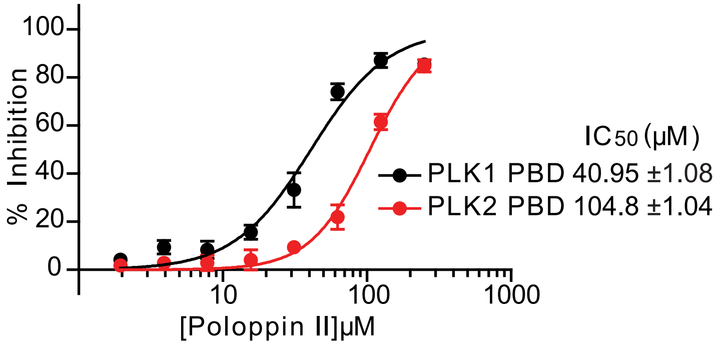

E

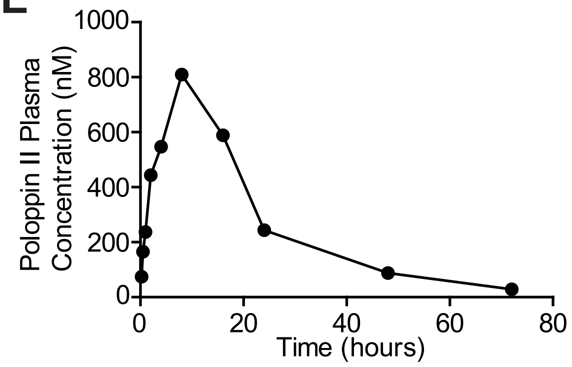

F

Treatment group	Day 1	Day 4	Day 7	Day 10	Day 13
Vehicle	✓	✓	✓	✓	✓
Poloppin II 50mg/kg	✓	✓	✓	✓	✓
Poloppin II 200mg/kg	✓	✓	✓		

G

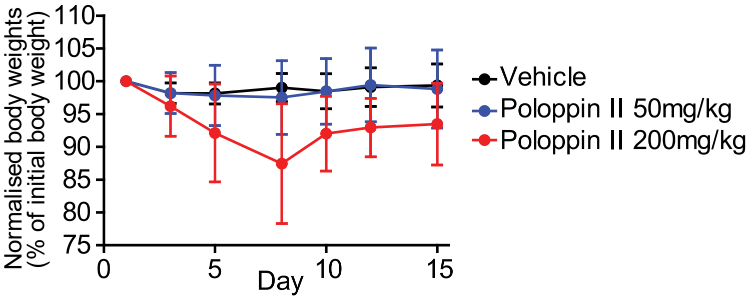

H

Treatment	Group Mean at Day 15	SD	Versus Vehicle (p-value)	Sig	%T/C value
Vehicle	778	172	-	-	-
Poloppin II 50mg/kg	415	159	<0.0001	****	53.3
Poloppin II 200mg/kg	371	125	<0.0001	****	47.7

I

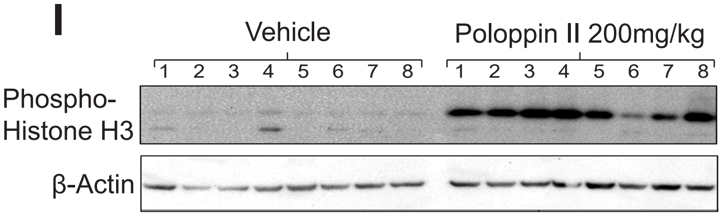

Figure S4. Related to Figure 6. Poloppin resistance develops less readily than to an ATP-competitive PLK1 inhibitor. cDNA extracted from drug-sensitive HCT116 wild-type or HCT116 BI 2536-resistant clones was amplified for PLK1, PLK2 and PLK3 sequences before next-generation sequencing (Illumina), before comparison against reference sequences. The D16G mutation found in wild-type HCT116 cells appears to be a germline mutation, whereas the R136G mutation in BI 2536-resistant clones conforms with a previously observed 'gatekeeper mutation' at the ATP-binding site that confers PLK1-inhibitor resistance (Wacker et al., 2012).

FIGURE S4

Summary Of Mutations In PLK1i-Resistant Cell Lines Analysed By NGS

	**Significant Base Variants**									**Corresponding Coding Muta ons**							
	PLK1		PLK2				PLK3		PLK1		PLK2				PLK3	
HCT116wt	A158G		T640C	T1168C	G1201A	T1849C	A1373G	G1595C	D16G		-	-	-	-	-	-
BI 2536-Resistant #1	A158G	A517G	T640C	T1168C	G1201A	T1849C	A1373G	G1595C	D16G	R136G	-	-	-	-	-	-
BI 2536-Resistant #2	A158G	A517G	T640C	T1168C	G1201A	T1849C	A1373G	G1595C	D16G	R136G	-	-	-	-	-	-
BI 2536-Resistant #3	A158G	A517G	T640C	T1168C	G1201A	T1849C	A1373G	G1595C	D16G	R136G	-	-	-	-	-	-
BI 2536-Resistant #4	A158G	A517G	T640C	T1168C	G1201A	T1849C	A1373G	G1595C	D16G	R136G	-	-	-	-	-	-
BI 2536-Resistant #5	A158G	A517G	T640C	T1168C	G1201A	T1849C	A1373G	G1595C	D16G	R136G	-	-	-	-	-	-
BI 2536-Resistant #6	A158G	A517G	T640C	T1168C	G1201A	T1849C	A1373G	G1595C	D16G	R136G	-	-	-	-	-	-
BI 2536-Resistant #7	A158G	A517G	T640C	T1168C	G1201A	T1849C	A1373G	G1595C	D16G	R136G	-	-	-	-	-	-
BI 2536-Resistant #8	A158G	A517G	T640C	T1168C	G1201A	T1849C	A1373G	G1595C	D16G	R136G	-	-	-	-	-	-
BI 2536-Resistant #9	A158G	A517G	T640C	T1168C	G1201A	T1849C	A1373G	G1595C	D16G	R136G	-	-	-	-	-	-

Figure S5. Related to Figure 7. Poloppins sensitize mutant KRAS-expressing cells to clinically used inhibitors of the c-MET tyrosine kinase via a synergistic mechanism. (A) Efficiency of c-MET depletion by siRNA after 96 h assessed by Western blot in SW48 Parental and SW48 KRAS G12D cell lines. (B) Increased cell death induced by Poloppin in human pancreatic cancer cell lines expressing KRAS G12D (Panc-1, HPAF-II, Panc 02.03 and Panc 05.04) after depletion of c-MET and 72 h compound exposure. Non-targeting All Stars siRNA control (black) is compared to c-MET depletion (red). Efficiency of c-MET depletion using siRNA is assessed by Western blot after 96 h downregulation. (C) Cell viability using combinations of different c-MET inhibitors (Crizotinib, Tivatinib and Foretinib) with Poloppin at GI_{25} in Panc-1 cancer cells that express mutant KRAS G12D. Viability was measured by SRB assay after 72 h exposure. (D) Cell viability in Panc-1 cells following depletion of PLKs by siRNA and 72 h exposure to c-MET inhibitors, Crizotinib and Tivatinib. Data represent the mean of three independent experiments ± SEM.

FIGURE S5

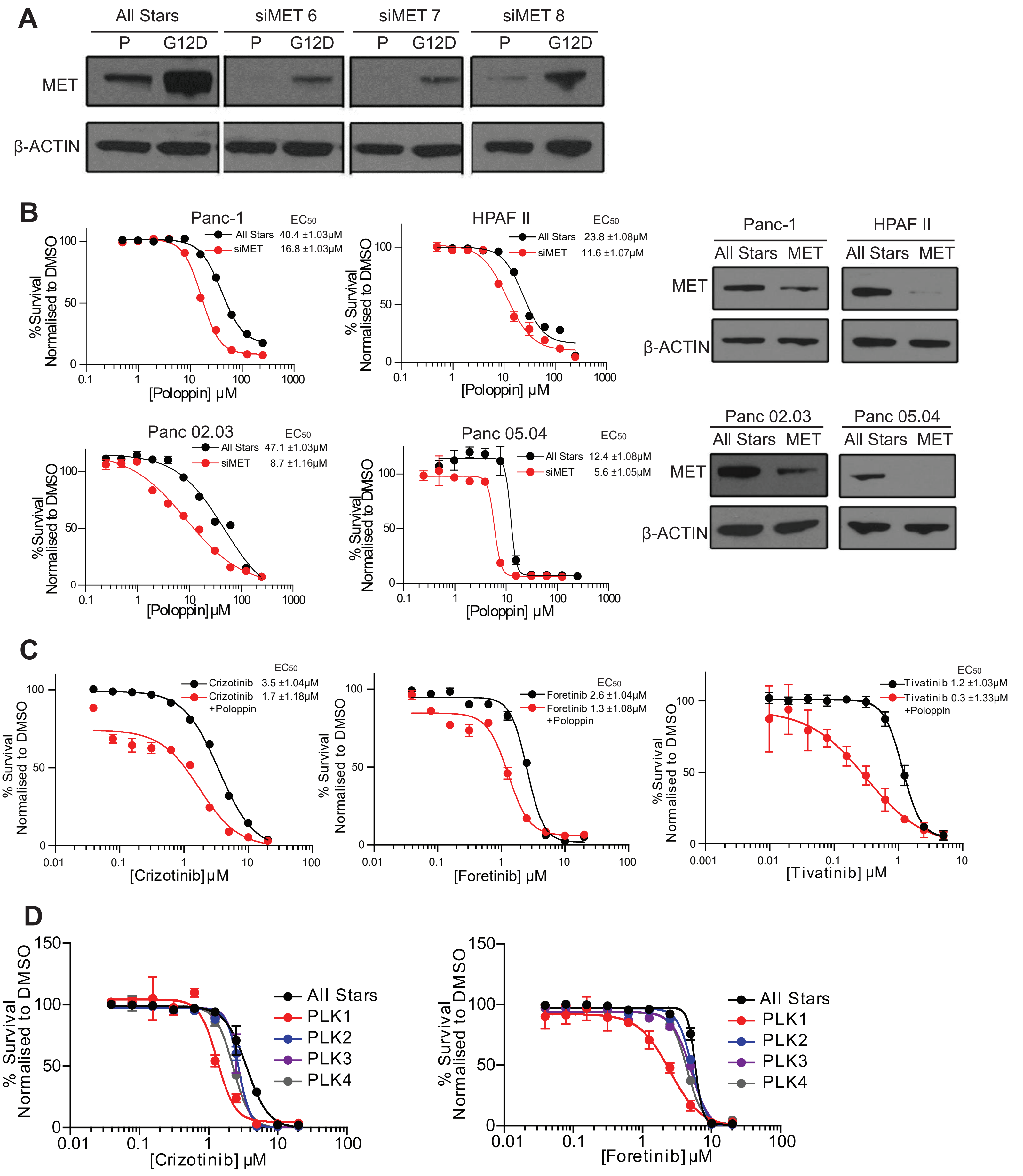
A
All Stars
siMET 6
siMET 7
siMET 8
P
G12D
MET
β-ACTIN
B
Panc-1
EC50
All Stars 40.4 ±1.03μM
siMET 16.8 ±1.03μM
HPAF II
EC50
All Stars 23.8 ±1.08μM
siMET 11.6 ±1.07μM
Panc 02.03
EC50
All Stars 47.1 ±1.03μM
siMET 8.7 ±1.16μM
Panc 05.04
EC50
All Stars 12.4 ±1.08μM
siMET 5.6 ±1.05μM
% Survival Normalised to DMSO
[Poloppin] μM
All Stars
MET
C
EC50
Crizotinib 3.5 ±1.04μM
Crizotinib 1.7 ±1.18μM +Poloppin
[Crizotinib] μM
Foretinib 2.6 ±1.04μM
Foretinib 1.3 ±1.08μM +Poloppin
[Foretinib] μM
Tivatinib 1.2 ±1.03μM
Tivatinib 0.3 ±1.33μM +Poloppin
[Tivatinib] μM
D
All Stars
PLK1
PLK2
PLK3
PLK4
[Crizotinib] μM
[Foretinib] μM

Table S1. Related to Figure 7. Poloppins sensitize mutant KRAS expressing cells to clinically used inhibitors of the c-MET tyrosine kinase via a synergistic mechanism.

Poloppin sensitizes mutant KRAS-expressing human cancer cell lines to c-MET inhibition with Crizotinib

Cell lines	Crizotinib GI_{50} (μM)	Crizotinib GI_{50} (μM) with Poloppin @ GI_{25}
Panc-1	7.24	0.28
HPAFII	N.D.	0.37
Panc 02.03	0.96	0.081
Panc 04.05	0.091	0.037
DLD-1	1.62	0.86
SW116	4.73	2.26
HT29	3.85	2.17

N.D., not determined
